# Five new sesquiterpenoids from agarwood of *Aquilaria sinensis*

**DOI:** 10.3762/bjoc.19.75

**Published:** 2023-06-30

**Authors:** Hong Zhou, Xu-Yang Li, Hong-Bin Fang, He-Zhong Jiang, Yong-Xian Cheng

**Affiliations:** 1 School of Life Science and Engineering, Southwest Jiaotong University, Chengdu, 610031, PR China; 2 Institute for Inheritance-Based Innovation of Chinese Medicine, Marshall Laboratory of Biomedical Engineering, School of Pharmacy, Shenzhen University Medical School, Shenzhen University, Shenzhen, Guangdong, 518055, China

**Keywords:** agarwood, *Aquilaria sinensis*, SAR studies, sesquiterpenoids

## Abstract

Five new eudesmane-type sesquiterpenoids (aquisinenoids F–J (**1**–**5**)) and five known compounds (**6**–**10**) were isolated from the agarwood of *Aquilaria sinensis*. Their structures, including absolute configurations, were identified by comprehensive spectroscopic analyses and computational methods. Inspired by our previous study on the same kinds of skeletons, we speculated that the new compounds have anticancer and anti-inflammatory activities. The results did not show any activity, but they revealed the structure–activity relationships (SAR).

## Introduction

**Figure 1:**
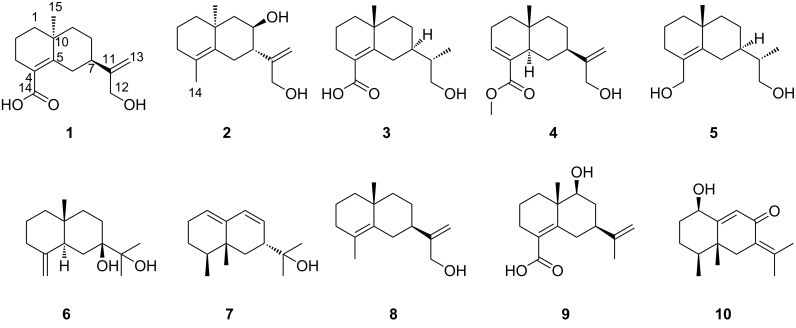
Structures of compounds **1**–**10**.

Agarwood is the resinous wood of the *Aquilaria* species of the Thymelaeaceae family [1]. It is a precious traditional Chinese medicinal material and a kind of natural fragrance that is widely distributed in China, India, the Middle East, and Southeast Asia [2]. Agarwood has been considered to play an important role in both traditional Chinese medicine and Ayurvedic medicine in the treatment of stomach disorders, coughs, asthma, sedation, analgesia, and antiemetic [3–4]. Previous studies have shown that 2-(2-phenylethyl)chromones and sesquiterpenes are the characteristic and main bioactive components of agarwood [5–6]. Various bioactivities, including neuroactive [4], gastrointestinal modulation [7], cytotoxicity [8], antibacterial [9], antifungal, acetylcholinesterase inhibition [8], anti-inflammatory [10], antiasthmatic [11], antidiabetic [12], and antioxidant [13] activities, have been reported for agarwood extracts [14–15]. Our group recently reported five structurally intriguing and biologically active sesquiterpene dimers [16], which attracted our interest to gain deep insight into novel molecules with effective bioactivities from agarwood. Therefore, the continued study of *Aquilaria sinensis* has led to the isolation of ten sesquiterpenoids, including five new eudesmane-type sesquiterpenoids (Figure 1). Herein, we describe the isolation, structural elucidation, and bioactivity evaluation of the new compounds.

## Results and Discussion

The dried and powdered agarwood sample was extracted by percolating with 95% EtOH to afford a crude extract, which was suspended in water followed by partitioning with EtOAc to afford an EtOAc-soluble extract. Furthermore, several methods were used to purify the extract, such as MCI gel CHP 20, silica gel column, vacuum liquid chromatography, and semi-preparative HPLC purification, to obtain pure compounds. A total of ten compounds, including five new eudesmane-type sesquiterpenoids (**1**–**5**), and five known compounds were identified. The known compounds are readily identified as eudesm-4(15)-ene-7β,11-diol (**6**) [17], *rel*-(2*R*,8*S*,8a*R*)-2-(1,2,6,7,8,8a-hexahydro-8,8a-dimethyl-2-naphthyl)propan-2-ol (**7**) [18], γ-costol (**8**) [19], (+)-9-hydroxyselina-4,11-dien-14-oic acid (**9**) [20] and 1β-hydroxyeremophila-7(11),9-dien-8-one (**10**) [21] by comparison of their spectroscopic data with those reported in the literature (Figure 1). The new derivatives were characterized as explained below.

**Figure 2:**
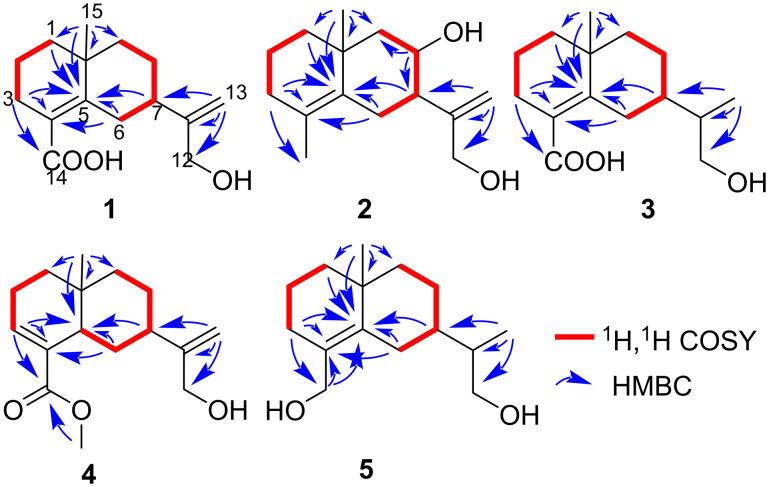
Key ^1^H,^1^H-COSY and HMBC correlations for **1**–**5**.

**Figure 3:**
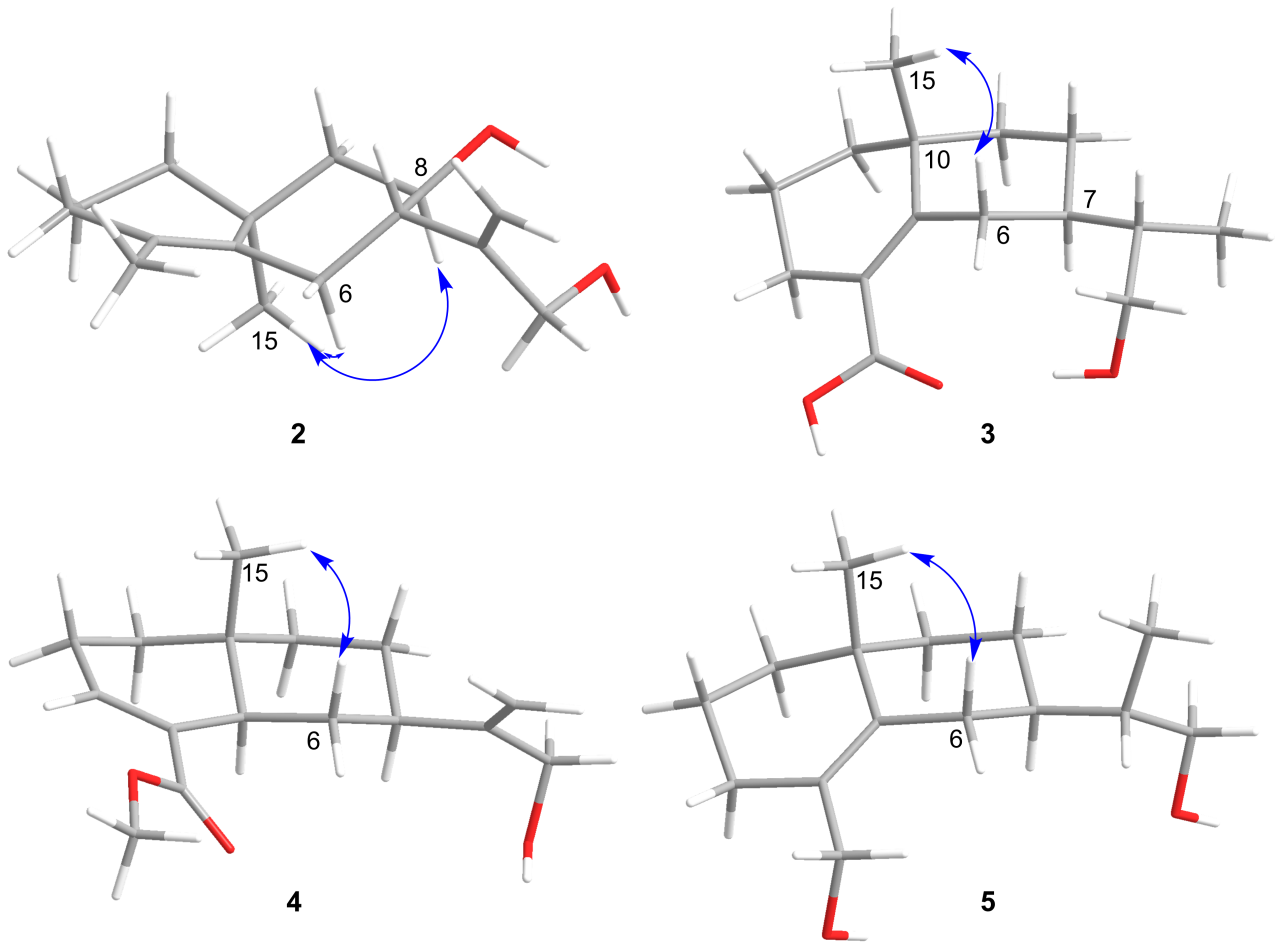
Key ROESY correlations for **2**–**5**.

**Figure 4:**
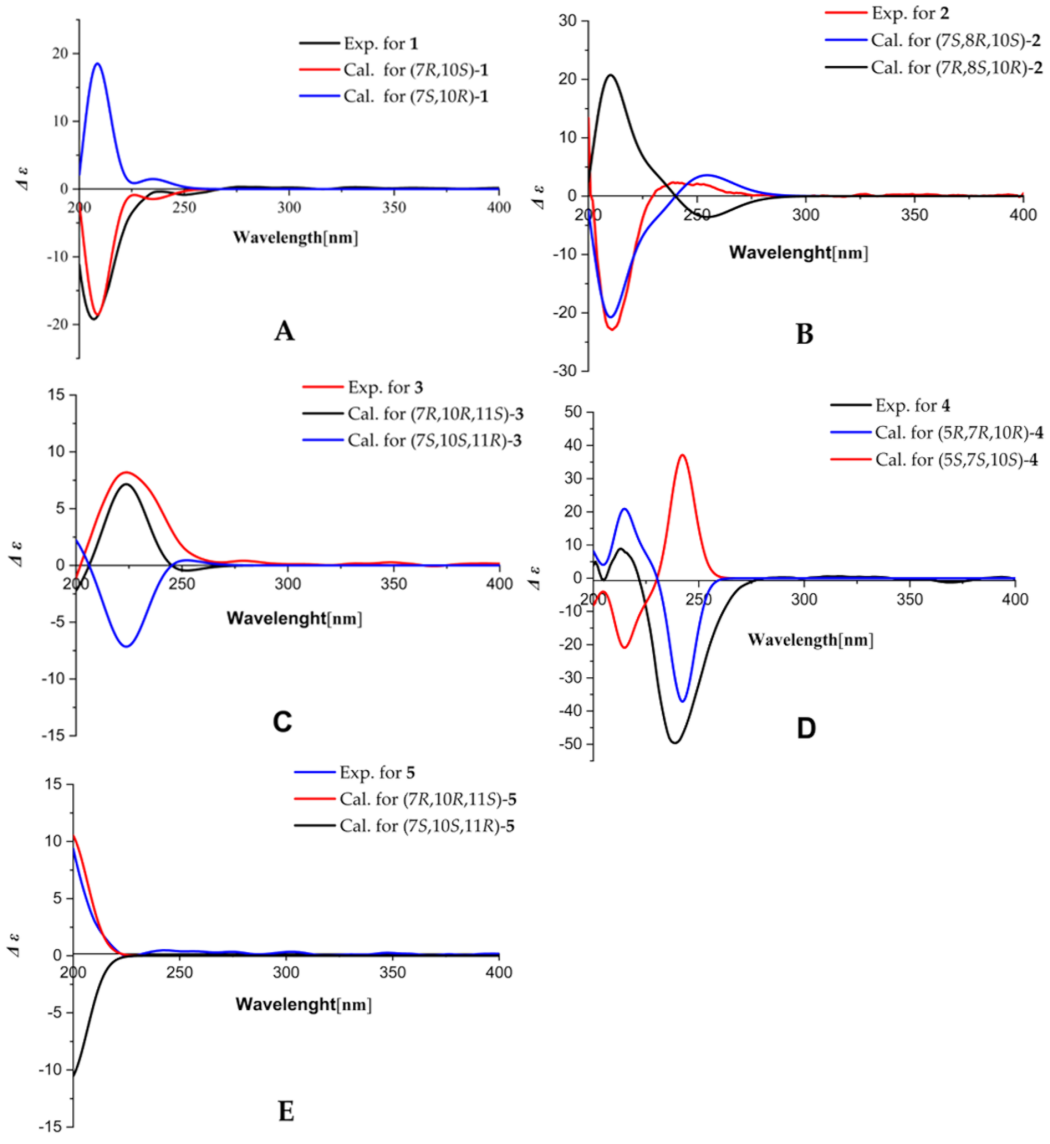
The calculated and experimental ECD spectra of **1**–**5**.

Compound **1** was obtained as pale yellow gum, and its molecular formula was inferred from the positive HRESIMS at *m*/*z* 273.1464 [M + Na]^+^ (calcd for C_15_H_22_O_3_Na, 273.1461), ^13^C NMR, and DEPT spectra, indicating 5 degrees of unsaturation. The ^1^H NMR spectrum of **1** (Table 1) shows one methyl group at δ_H_ 1.16 (s, 3H), two olefinic protons [δ_H_ 5.05 (d, *J* = 1.4 Hz, 1H), δ_H_ 4.05 (d, *J* = 1.4 Hz, 1H)], and an oxygenated methylene at δ_H_ 4.06 (s, 2H). The ^13^C NMR and DEPT (Table 1) spectra indicate 15 carbons, including one methyl, eight methylenes (one sp^2^), one methine, and four nonprotonated carbons (including three sp^2^ and one sp^3^). The planar structure of **1** was mainly constructed by 2D NMR analysis. First, the ^1^H,^1^H-COSY spectrum (Figure 2) displays the correlations of H-1/H-2/H-3 and H-6/H-7/H-8/H-9, suggesting the existence of two spin systems. The HMBC correlations (Figure 2) of H-1/C-5 (δ_C_ 148.3), H-3/C-4 (δ_C_ 126.8), C-5 and H_3_-15/C-1 (δ_C_ 40.6), C-5, and C-10 (δ_C_ 36.2) indicate the presence of a six-membered ring. Additional HMBC correlations of H-6/C-4, C-5, H-7/C-5, and H_3_-15/C-9 (δ_C_ 43.0) allowed us to assign another six-membered ring, as shown in Figure 2. Furthermore, the positions of a methyl group at C-10 were clarified by the HMBC correlations of H_3_-15/C-1, C-5, C-9, and C-10, respectively. In addition, the HMBC correlations of H-3/C-14 (δ_C_ 174.9) and H-6/C-4 indicates the presence of a carboxyl group at C-4. Finally, the HMBC correlations of H-13/C-7 (δ_C_ 43.9), C-11 (δ_C_ 154.7), and C-12 (δ_C_ 65.0) demonstrate that C-7 is connected with acryl alcohol. Thus, the planar structure of **1** was assigned (Figure 1). However, the ROSEY data cannot provide the correlation of H_3_-15/H_2_-6 (Figure 3), which results in ambiguity in the relative configuration assignment of **1**. Thus, NMR chemical shift calculations and ECD calculations were used to confirm the relative and absolute configuration of **1**. More specifically, NMR calculations were carried out at the PCM/mPW1PW91/6-311+G(d,p) [16] level for (7R*,10S*)-**1** (**1a**) and (7S*,10S*)-**1** (**1b**), which are possible diastereomers of **1**. The results reveal that **1a** has the highest probability score. Next, ECD calculations on (7*R*,10*S*)-**1** and (7*S*,10*R*)-**1** were conducted according to the results obtained from NMR calculations. The CD spectrum matched well with the calculated ECD spectrum of **1a** (Figure 4), revealing the absolute configuration of **1** to be 7*R*,10*S*, and it was named aquisinenoid F.

**Table 1:** ^1^H (500 MHz) and ^13^C (125 MHz) NMR data of **1** and **2** in MeOD_._

	**1**			**2**	

no.	δ_C_	δ_H_, mult (*J* in Hz)	no.	δ_C_	δ_H_, mult (*J* in Hz)

1	40.6, CH_2_	Ha: 1.59 (m)	1	41.3, CH_2_	Ha: 1.53 (m)
		Hb: 1.39 (m)	2		Hb: 1.35 (m)
2	19.4, CH_2_	1.67 (m)		19.9, CH_2_	Ha: 1.64 (m)
3	29.0, CH_2_	2.26 (m)			Hb: 1.53 (m)
4	126.8, C		3	33.9, CH_2_	Ha: 2.00 (overlap)
5	148.3, C				Hb: 1.91 (m)
6	34.4, CH_2_	Ha: 2.96 (m)	4	126.6, C	
		Hb: 2.03 (m)	5	134.4, C	
7	43.9, C	2.02 (m)	6	32.3, CH_2_	Ha: 2.58 (dd 14.3, 3.8)
8	28.9, CH_2_	Ha: 1.69 (m)			Hb: 2.00 (overlap)
		Hb: 1.67 (m)	7	52.3, CH	1.80 (m)
9	43.0, CH_2_	Ha: 1.66 (m)	8	71.0, CH	3.88 (ddd 11.3, 10.3, 4.3)
		Hb: 1.41 (ddd, 16.1, 7.5, 4.2)	9	51.8, CH_2_	Ha: 1.80 (m)
10	36.2, C				Hb: 1.18 (m)
11	154.7, C		10	36.8, C	
12	65.0, CH_2_	4.06 (s)	11	152.6, C	
13	108.1, CH_2_	Ha: 5.05 (d 1.4)	12	65.9, CH_2_	4.10 (dt 4.4, 1.3)
		Hb: 4.05 (d 1.4)	13	110.6, CH_2_	Ha: 5.17 (d 1.5)
14	174.9, C				Hb: 5.02 (d 1.5)
15	25.2, CH_3_	1.16 (s)	14	19.5, CH_3_	1.61 (s)
			15	25.9, CH_3_	1.11 (s)

Compound **2** was isolated as pale yellow gum, and was assigned the molecular formula C_15_H_24_O_2_ as inferred from the HRESIMS *m*/*z* 259.1671 [M + Na]^+^ (calcd 259.1669). The ^13^C NMR and DEPT spectra (Table 1) of **2** indicate 4 degrees of unsaturation. Compound **2** is similar in structure to **1** by analysis of their NMR data. There are two differences between **2** and **1**. One is that at C-4 in **2** a methyl group is attached instead of a carboxyl group as in **1**, which is supported by the HMBC correlations of H-3/C-4 (δ_C_ 126.6), C-5 (δ_C_ 134.4), and C-14 (δ_C_ 19.5). The other difference is the existence of 8-OH in **2**, which is confirmed by downfield chemical shifts at δ_H_ 3.88 and δ_C_ 71.0 and the key HMBC correlations of H-8/C-7 and C-9. Hence, the planer structure of **2** was obtained (Figure 1). The relative configuration of **2** was established by careful interpretation of the ROESY correlations (Figure 3). The ROESY correlations of H-8/H_3_-15 and Ha-6/H_3_-15 and the coupling constant of H-7 (*J*_H-7, Ha-6_ = 3.8 Hz) suggest the relative configurations (7S*,8R*,10S*)-**2** (**2a**) or (7R*,8S*,10R*)-**2** (**2b**). To assign the absolute configuration of **2**, ECD calculations were performed for (7*S*,8*R*,10*S*)-**2** and (7*R*,8*S*,10*R*)-**2**. The results show that the calculated ECD spectrum of **2a** is in accordance with the experimental spectrum (Figure 4). The absolute configuration of **2** was eventually clarified to be 7*S*,8*R*,10*S*, and it was named aquisinenoid G.

Compound **3** was isolated as pale yellow gum, and its molecular formula was determined to be C_15_H_24_O_3_ based on its HRESIMS *m*/*z* 275.1622 [M + Na]^+^ (calcd for C_15_H_24_O_3_Na, 275.1618), ^13^C NMR and DEPT spectra (Table 2), indicating 4 degrees of unsaturation. Detailed NMR interpretation implies that the data of **3** are similar to those of **1**. The only difference is that the C-11 double bond in **1** was saturated in **3**, which was verified by the HMBC correlations (Figure 2) of H-13/C-7 (δ_C_ 41.9), C-11 (δ_C_ 42.6), and C-12 (δ_C_ 66.3). Thus, the planar structure of **3** was assigned (Figure 1). Further analysis of the coupling constants of H-7 (*J*_H-7, Ha-6_ = 3.3 Hz and *J*_H-7, Hb-6_ = 2.8 Hz) and the ROESY correlation (Figure 3) of H_3_-15/Hb-6 allowed us to conclude that H_3_-15 and H-7 are in the opposite orientation. Thus, the relative configurations of chiral centers in **3** apart from C-11 were assigned. To determine the configuration of C-11 we performed NMR calculations. The results disclose that **3** has likely the configuration of (7R*,10R*,11S*)-**3** based on the DP4+ probability analysis and the correlation coefficient. To clarify the absolute configuration of **3**, ECD calculations were carried out. The spectrum of (7*R*,10*R*,11*S)-***3** (Figure 4) agreed with the experimental spectrum, suggesting the 7*R*,10*R*,11*S* configuration of **3**. As a result, the structure of **3** was determined as shown (Figure 1) and named aquisinenoid H.

**Table 2:** ^1^H (500 MHz) and ^13^C (150 MHz) NMR data of **3** and **4** in MeOD.

	**3**			**4**	

no.	δ_C_	δ_H_, mult (*J* in Hz)	no.	δ_C_	δ_H_, mult (*J* in Hz)

1	40.8, CH_2_	Ha: 1.54 (m)	1	37.8, CH_2_	Ha: 1.44 (dd 13.3, 6.2)
		Hb: 1.35 (m)			Hb: 1.37 (m)
2	19.6, CH_2_	1.64 (m)	2	24.2, CH_2_	Ha: 2.27 (m)
3	29.1, CH_2_	2.23 (m)			Hb: 2.18 (m)
4	127.7, C		3	138.6, CH	6.58 (m)
5	146.5, C		4	135.1, C	
6	31.2, CH_2_	Ha: 2.80 (dd 13.8, 3.3)	5	45.1, CH	2.22 (m)
		Hb: 1.78 (tt 13.8, 2.8)	6	29.7, CH_2_	Ha: 2.17 (m)
7	41.9, CH	1.44 (m)			Hb: 1.12 (q 12.3)
8	27.4, CH_2_	Ha: 1.53 (m)	7	43.1, CH	2.10 (m)
		Hb:1.62 (m)	8	28.5, CH_2_	1.65 (m)
9	43.1, CH_2_	Ha: 1.61 (m)	9	41.1, CH_2_	Ha: 1.51 (dt 12.9, 3.9)
		Hb: 1.37 (m)			Hb: 1.31 (m)
10	36.1, C		10	33.5, C	
11	42.6, CH	1.51 (m)	11	155.3, C	
12	66.3, CH_2_	3.57 (dd 10.8, 5.5)	12	65.2, CH_2_	4.06 (s)
		3.38 (dd 10.8, 4.2)	13	108.2, CH_2_	Ha: 5.03 (q 1.6)
13	13.8, CH_3_	0.92 (d 6.9)			Hb: 4.85 (q 1.3)
14	176.3, C		14	170.3, C	
15	25.1, CH_3_	1.11 (s)	15	51.8, CH_3_	3.68 (s)
			16	16.0, CH_3_	0.87 (s)

Compound **4**, obtained as a white powder, possesses a molecular formula of C_16_H_24_O_3_ (5 degrees of unsaturation) derived from its HRESIMS (*m/z* 287.1614, calcd 287.1618 [M + Na]^+^). Comparing the NMR data of **1** with those of **4** indicates that the Δ^4,5^ double bond migrates to Δ^3,4^ and the carboxylic acid group becomes a methyl ester derivative, which were confirmed by ^1^H,^1^H-COSY correlations (Figure 2) of H-5/H-6/H-7 and the HMBC correlations (Figure 2) of H-3/C-4 (δ_C_ 135.1), C-14 (δ_C_ 170.3), and H_3_-15/C-14, as well as characteristic chemical shifts at C-3 (δ_C_ 138.6). Thus, the planar structure of **4** was assigned. The opposite orientations of H-5 and Hb-6, as well as Hb-6 and H-7, were proven by the coupling constants of Ha-6 (*J*_H-5, Hb-6_ = 12.3 Hz and *J*_H-7, Hb-6_ = 12.3 Hz), and the combined ROESY (Figure 3) correlation of H_3_-16/Hb-6 indicated that its relative configuration was (5R*,7R*,10R*)-**4**. To elucidate its absolute configuration, the CD spectrum was determined and compared with the calculated spectrum. The results show that the calculated ECD spectrum of (5R*,7R*,10R*)-**4** (Figure 4) matches well with the experimental spectrum, suggesting that the absolute configuration of **4** is 5*R*,7*R*,10*R*. As a result, the absolute configuration of **4** was finally confirmed, and it was named aquisinenoid I.

Compound **5**, obtained as colorless gum, had a molecular formula of C_15_H_26_O_2_ (3 degrees of unsaturation) derived from its HRESIMS (*m*/*z* 261.1825, calculated 261.1825 [M + Na]^+^). The 1D NMR spectra of **5** exhibit a pattern analogous to that of **3** (Table 3). The difference is that the carboxylic acid at C-4 in **3** undergoes reduction to form a hydroxymethyl group at C-4 in **5**. This alteration is supported by the analysis of the HMBC correlations (Figure 2) of H-3/C-4 (δ_C_ 128.6), C-14 (δ_C_ 63.5) and H-14/C-4, C-5 (δ_C_ 141.9). Thus, the planar structure of **5** was assigned. Analysis of the coupling constants of H-7 (*J*_H-7, Ha-6_ = 2.6 Hz) and the ROESY (Figure 3) correlations of H_3_-15/Hb-6 show that H_3_-15 and H-7 are in the opposite orientation. NMR calculations were performed to clarify the relative configuration at C-11. Finally, the relative configuration of **5** was assigned as (7R*,10R*,11S*)-**5** by conducting NMR calculations at the PCM/mPW1PW91/6-311+G(d,p) level, calculated for (7S*,10S*,11S*)-**5** (**5a**) and (7R*,10R*,11S*)-**5** (**5b**) using the correlation coefficient and DP4+ probability analysis. The absolute configuration of **5** was subsequently assigned by direct ECD calculation of (7*R*,10*R*,11*S)*-**5**. The results show that the spectrum of **5b** agrees well with the experimental spectrum of **5** (Figure 4), showing the absolute configuration of **5** to be 7*R*,10*R*,11*S*. Thus, the structure of **5**, named aquisinenoid J, was finally identified.

**Table 3:** ^1^H (500 MHz) and ^13^C (150 MHz) NMR data of **5** in CDCl_3_.

**5**					

no.	δ_C_	δ_H_, mult (*J* in Hz)	no.	δ_C_	δ_H_, mult (*J* in Hz)

1	40.1, CH_2_	Ha: 1.52 (m)	9	42.3, CH_2_	Ha: 1.56 (m)
		Hb: 1.31 (m)			Hb: 1.34 (m)
2	19.3, CH_2_	1.60 (m)	10	25.0, C	
3	30.2, CH_2_	Ha: 2.13 (m)	11	40.3, CH	1.63 (m)
		Hb: 2.09 (m)	12	66.2, CH_2_	Ha: 3.61 (dd 10.9, 6.9)
4	128.6, C				Hb: 3.53 (dd 10.9, 6.9)
5	141.9, C		13	12.9, CH_3_	0.89 (d 6.9)
6	27.1, CH_2_	Ha: 2.53 (dt 13.6, 2.6)	14	63.5, CH_2_	Ha: 4.12 (d 11.8)
		Hb: 1.67 (m)			Hb: 3.99 (d 11.8)
7	41.3, CH	1.47 (m)	15	25.0, CH_3_	1.05 (s)
8	26.9, CH_2_	1.44 (m)			

According to our previous studies, the components from *A. sinensis* possess various attractive bioactivities, such as anti-inflammatory, anticancer, antirenal fibrosis, and acetylcholinesterase inhibitory effects, which motivate us to assume that compounds with similar skeletons may have the same bioactivities. Therefore, the new compounds were evaluated for their anti-inflammatory and anticancer potential using the same method as described previously [16,22–23], and the cell viability was determined by CCK-8 assays (Figures A and B, Supporting Information File 1). Unfortunately, we could not distinguish any one of these activities for all the new derivatives. Furthermore, we recall the skeletons in our current study and our previously reported ones [16,22–26], which revealed the SAR. Moreover, eudesmane-type sesquiterpenes constructed with aldehyde groups are more active even in the form of dimers. In the present study, it was described that skeletons with 1°-alcohols and/or acid groups suppress activity, which was consistent with the *A. sinensis* literatures that 1°-alcohols and/or acid groups suppress activity [25,27], and concluded that eudesmane-type sesquiterpenes constructed with aldehyde groups are more active than alcohols or acids.

## Conclusion

In summary, five new eudesmane-type sesquiterpenes compounds (**1**–**5**) and five known compounds (**6**–**10**) were isolated from agarwood of *A. sinensis*. The discovery of these new compounds enriches the structural diversity and complexity of sesquiterpenes derived from agarwood. Unfortunately, none of the new compounds exhibits biological activity against LPS-induced inflammation in Raw264.7 cells and human breast cancer cells. However, we have drawn good conclusions for SAR studies based on the current study and our previous study. These compounds will be isolated by other researchers in the future, who could consider our conclusions and choose other aspects of biological activity to study.

## Experimental

### General procedures

NMR spectra were recorded on a Bruker AV-500 or AV-600 spectrometer with TMS as an internal standard. Silica gel (200–300 mesh; Qingdao Marine Chemical Inc., Qingdao, China), RP-18 silica gel (40–60 µm; Daiso Co., Tokyo, Japan), and MCI gel CHP 20P (75–150 µm, Mitsubishi Chemical Industries, Tokyo, Japan) were used for column chromatography. Optical rotations were measured on an Anton Paar MCP-100 digital polarimeter. UV and CD spectra were obtained on a Jasco J−815 circular dichroism spectrometer (JASCO, Tokyo, Japan). Semi-preparative HPLC was carried out using an Agilent 1260 chromatograph with a 250 mm × 10 mm column, i.d., 5 μm, SEP Basic 120 C18. HRESIMS were measured on a SCIEX X500R QTOF MS spectrometer (Shimadzu Corporation, Tokyo, Japan).

### Plant material

The resinous wood of *Aquilaria sinensis* was purchased from Hainan Xiangshu Agarwood Industry Groud Co., Ltd., July 2018. The material was identified by the Gansu Institute for Drug Control, and a voucher specimen (CHYX0642) was deposited at School of Pharmaceutical Sciences, Shenzhen University, P.R. China.

### Extraction and isolation

The dried and powdered agarwood sample (15.0 kg) was extracted by percolating with 95% EtOH to afford a crude extract, which was suspended in water followed by partition with EtOAc to afford an EtOAc-soluble extract (1.7 kg). The EtOAc extract was separated by a MCI gel CHP 20P column eluted with gradient aqueous MeOH (50–100%) to provide nine portions (Fr.1–Fr.9). Fr.6 (144.0 g) was separated into fourteen fractions (Fr.6.1–Fr.6.14) by a silica gel column with petroleum ether/EtOAc (50:1–0:1). Fr.6.3 (1.3 g) was further divided into four parts (Fr.6.3.1–Fr.6.3.4) by a vacuum liquid chromatography (VLC) on a silica gel column with petroleum ether/acetone (50:1–3:7) as solvents. Fr.6.3.1 (808.5 mg) was subjected to preparative thin-layer chromatography (PTLC) (dichloromethane) to give Fr.6.3.1.1–Fr.6.3.1.7, of which Fr.6.3.1.4 (255.9 mg) was purified by preparative HPLC on YMC-Pack-ODS-A with aqueous MeCN (63%), and then purified by semi-preparative HPLC on YMC-Pack-ODS-A with aqueous MeOH (82%) to afford **7** (36.1 mg, *t*_R_ = 21.3 min; flow rate: 3 mL/min). Fr.6.5 (9.3 g) was further fractionated into ten parts (Fr.6.5.1–Fr.6.5.10) by a silica gel column washed with petroleum ether/EtOAC (50:1–1:1). Among them, Fr.6.5.7 (355.7 mg) was subjected to PTLC (petroleum ether/acetone 5:1) to give Fr.6.5.7.1–Fr.6.5.7.7. Fr.6.5.7.1 (34.3 mg) was purified by semi-preparative HPLC on SEP Basic 120 C18 (aqueous MeOH, 65%) to give compound **9** (4.9 mg, *t*_R_ = 9.4 min, flow rate: 3 mL/min). Fr.6.5.8 (787.3 mg) was separated by VLC on silica gel eluted with petroleum ether/acetone (25:1–1:1) to provide six portions (Fr.6.5.8.1–Fr.6.5.8.6). Fr.6.5.8.4 (91.0 mg) was further purified by semi-preparative HPLC on SEP Basic 120 C18 with aqueous MeCN (46%) to afford **10** (4.5 mg, *t*_R_ = 24.9 min; flow rate: 3 mL/min). Fr.6.6 (9.1 g) was further divided into fifteen parts (Fr.6.6.1–Fr.6.6.15) by a YMC-ODS column (MeOH, 45–80%). Fr.6.6.10 (747.8 g) was separated a by silica gel column washed with petroleum ether/acetone (1:9–6:4) to yield thirteen portions (Fr.6.6.10.1–Fr.6.6.10.13). Fr.6.6.11 (333.0 mg) was separated by preparative TLC with petroleum ether/acetone (3:1) to obtain five fractions (Fr.6.6.11.1–Fr.6.6.11.5). Compound **6** (7.9 mg, *t*_R_ =17.4 min, flow rate: 3 mL/min) was obtained by semi-preparative HPLC on SEP Basic 120 C18 (MeCN, 50%) from Fr.6.6.11.2 (60.5 mg), and compound **4** (11.5 mg, *t*_R_ = 20.6 min, flow rate: 3 mL/min) was obtained by semi-preparative HPLC on SEP Basic 120 C18 (aqueous MeOH, 73%) from Fr.6.6.11.3 (60.4 mg). Fr.6.7 (7.1 g) was further divided into ten parts (Fr.6.7.1–Fr.6.7.10) by a YMC-ODS column (MeOH, 45–70%). Fr.6.7.9 (4.9 g) was separated into eight fractions (Fr.6.7.9.1–Fr.6.7.9.8) by silica gel eluted with petroleum ether/acetone (6:94–55:45). Fr.6.7.9.5 (2.3 g) was further fractionated into seventeen parts (Fr.6.7.9.5.1–Fr.6.7.9.5.17) by a silica gel column washed with petroleum ether/acetone (6:94–55:45). Fr.6.7.9.5.14 (296.5 mg) was separated by PTLC (petroleum ether/isopropyl alcohol 10:1) to afford eight fractions (Fr.6.7.9.5.14.1–Fr.6.7.9.5.14.8). Fr.6.7.9.5.14.3 (25.4 mg) was further purified by semi-preparative HPLC on YMC-PACK-ODS-A (aqueous MeCN, 65%) to yield **1** (12.7 mg, *t*_R_ = 28.1 min, flow rate: 3 mL/min). Fr.6.7.9.5.14.5 (53.5 mg) was further purified by semi-preparative HPLC on SEP Basic 120 C18 (aqueous MeCN, 65%) to yield **3** (1.8 mg, *t*_R_ = 18.0 min, flow rate: 3 mL/min). Fr.6.9 (7.7 g) was further divided into thirteen parts (Fr.6.9.1–Fr.6.9.13) by a YMC-ODS column (MeOH, 45–85%). Fr.6.9.7 (2.8 g) was separated into seven fractions (Fr.6.9.7.1–Fr.6.9.7.7) by using a silica gel column with petroleum ether/acetone (5:95–1:1). Fr.6.9.7.5 (559.6 mg) was separated by preparative TLC (petroleum ether/acetone 3:1) to give Fr.6.9.7.5.1–Fr.6.9.7.5.5, of which Fr.6.9.7.5.5 (35.1 mg) was purified by semi-preparative HPLC on SEP Basic 120 C18 (aqueous MeOH, 65%) to afford compound **2** (7.6 mg, *t*_R_ = 17.3 min, flow rate: 3 mL/min). Fr.6.11(2.8 g) was gel filtrated over Sephadex LH-20 (MeOH) to afford eight parts (Fr.6.11.1–Fr.6.11.8). Fr.6.11.2 (792.9 mg) was separated by VLC on silica gel eluted with petroleum ether/acetone (9:91–60:40) to provide seven portions (Fr.6.11.2.1–Fr.6.11.2.7). Fr.6.11.2.3 (46.2 mg) was further purified by semi-preparative HPLC (aqueous MeCN, 42%) to yield **8** (2.6 mg, *t*_R_ = 25.5 min, flow rate: 3 mL/min). Fr.6.11.1 (660.0 mg) was separated by VLC on silica gel eluted with petroleum ether/acetone (10:90–55:45) to provide nine portions (Fr.6.11.1.1–Fr.6.11.1.9). Fr.6.11.1.5 (142.2 mg) was separated by preparative TLC (petroleum ether/acetone–3:1) to give Fr.6.11.1.5.1–Fr.6.11.1.5.4. The last part (63.6 mg) was submitted to semi-preparative HPLC on YMC-Pack-ODS-A (aqueous MeCN, 43%) to produce compound **5** (11.6 mg, *t*_R_ = 25.6 min, flow rate: 3 mL/min).

### Compound characterization data

Compound **1**: Pale yellow gum. [α]_D_^20^ +33.33 (*c* 0.3, MeOH); CD (MeOH) Δε_201_ −2.01, Δε_208_ −3.21; UV (MeOH) λ_max_ (log ε) 200 (2.89) nm, 222 (2.73) nm; HRESIMS (*m*/*z*): [M + Na]^+^ calcd for C_15_H_22_O_3_Na, 273.1461; found, 273.1464; ^1^H and ^13^C NMR data, see Table 1.

Compound **2**: Pale yellow gum. [α]_D_^20^ +18.25 (*c* 0.4, MeOH); CD (MeOH) Δε_200_ +2.99, Δε_211_ −5.12, Δε_239_ +0.53; UV(MeOH) λ_max_ (log ε) 200 (3.02) nm, 246 (2.11) nm; HRESIMS (*m*/*z*): [M + Na]^+^ calcd for C_15_H_24_O_2_Na, 259.1669; found, 259.1671; ^1^H and ^13^C NMR data, see Table 1.

Compound **3**: Pale yellow gum. [α]_D_^20^ +26.00 (*c* 0.5, MeOH); CD (MeOH) Δε_202_ −0.16, Δε_224_ +1.31; UV(MeOH) λ_max_ (log ε) 223 (2.34) nm; HRESIMS (*m*/*z*): [M + Na]^+^ calcd for C_15_H_24_O_3_Na, 275.1618; found, 275.1622; ^1^H and ^13^C NMR data, see Table 2.

Compound **4**: White powder. [α]_D_^20^ +20.99 (*c* 0.3, MeOH); CD (MeOH) Δε_203_ −0.10, Δε_211_ −1.43, Δε_238_ −7.89; UV (MeOH) λ_max_ (log ε) 218 (2.85) nm; HRESIMS (*m*/*z*): [M + Na]^+^ calcd for C_16_H_24_O_3_Na, 287.1618; found, 287.1614; ^1^H and ^13^C NMR data, see Table 2.

Compound **5**: Colorless gum. [α]_D_^20^ +74.00 (*c* 0.5, MeOH); CD (MeOH) Δε_201_ +2.69, Δε_227_ −0.05; UV (MeOH) λ_max_ (log ε) 200 (2.82) nm; HRESIMS (*m*/*z*): [M + Na]^+^ calcd for C_15_H_26_O_2_Na, 261.1825; found, 261.1825; ^1^H and ^13^C NMR data, see Table 3.

### Calculations of NMR spectra

Using density functional theory (DFT) and B3LYP/6-31G(d,p) [28] levels in the Gaussian 09 software package [29], the obtained minimum energy conformation of the force field was optimized. DFT was used to calculate the gauge-independent atomic orbital [30] for ^1^H and ^13^C NMR chemical shifts using the PCM solvent model in Gaussian 09 software [29]. The NMR chemical shift was corrected by the isotope shift of TMS [31]. The calculated ^13^C NMR chemical shift was analyzed by regression with the experimental one. Boltzmann weighing of the predicted chemical shift of each isomer and the DP4+ parameters were calculated using the Excel file provided by Ariel M. Sarotti [32].

### Calculations of ECD spectra

Using the Spartan'14 software package (Wavefunction Inc., Irvine, CA, USA) and the Gaussian 09 software package, the conformational search was performed using the molecular Merck force field (MMFF) [33] with CONFLEX 7.0 software [23]. Generally, the next calculation is performed by selecting an energy difference of less than 10 kcal/mol. ECD calculations were further conducted at the B3LYP/6-31G(d,p) level with PCM in MeOH [16]. SpecDis 1.62 [34] was used to compare the calculated curves and experimental CD spectra.

## Supporting Information

File 1 MS, UV, and NMR spectra of compounds **1**–**5**, NMR and ECD calculations, and bioactivity assay data.
