# Supplementary material for: Five new sesquiterpenoids from agarwood of Aquilaria sinensis
Source: Beilstein J Org Chem. 2023 Jun 30;19:998–1007. doi: 10.3762/bjoc.19.75 (PMC10315886; doi:10.3762/bjoc.19.75)
Supplement: File 1 — MS, UV, and NMR spectra of compounds 1–5, NMR and ECD calculations, and bioactivity assay data. [file Beilstein_J_Org_Chem-19-998-s001.pdf]

BEILSTEIN JOURNAL OF ORGANIC CHEMISTRY

Supporting Information

for

Five new sesquiterpenoids from agarwood of *Aquilaria sinensis*

Hong Zhou, Xu-Yang Li, Hong-Bin Fang, He-Zhong Jiang and Yong-Xian Cheng

Beilstein J. Org. Chem. **2023,** *19,* 998–1007. doi:10.3762/bjoc.19.75

MS, UV, and NMR spectra of compounds 1–5, NMR and ECD calculations, and bioactivity assay data

License and Terms: This is a supporting information file under the terms of the Creative Commons Attribution License (https://creativecommons.org/licenses/by/4.0). Please note that the reuse, redistribution and reproduction in particular requires that the author(s) and source are credited and that individual graphics may be subject to special legal provisions.
The license is subject to the *Beilstein Journal of Organic Chemistry* terms and conditions: (https://www.beilstein-journals.org/bjoc/terms)

Contents

Figure S1: The lowest energy conformers of **1** (the relative populations are in parentheses).

Figure S2: The lowest energy conformers of **2** (the relative populations are in parentheses).

Figure S3: The lowest energy conformers of **3** (the relative populations are in parentheses).

Figure S4: The lowest energy conformers of **4** (the relative populations are in parentheses).

Figure S5: The lowest energy conformers of **5** (the relative populations are in parentheses).

Table 1: Extracted heats and weighting factors of the optimized conformers of **1**–**5** at B3LYP/6-31G(d,p) level.

Table 2: The Cartesian coordinates of the lowest energy conformers for **1–5**.

Figure S6: DP4$^+$ results of candidate(7*R*,10*R*)-**1** (Isomer 1) and (7*R*,11*S*)-**1** (Isomer 2).

Figure S7: DP4$^+$ results of candidate (7*R*,10*R*, 11*R)*-**3** (Isomer 1) and (7*R*,10*R*,11*S*)-**3** (Isomer 2).

Figure S8: DP4$^+$ results of candidate(7*S*,10*S*,11*S)*-**5** (Isomer 1) and (7*R*,10*R*,11*S*)-**5** (Isomer 2).

Figure S9: ^{1}H NMR (500 MHz) spectrum of **1** in CD_3OD.

Figure S10: ^{13}C NMR and DEPT (125 MHz) spectra of **1** in CD_3OD.

Figure S11: ^{1}H-^{1}H COSY (500 MHz) spectrum of **1** in CD_3OD.

Figure S12: HSQC (500 MHz) spectrum of **1** in CD_3OD.

Figure S13: HSBC (500 MHz) spectrum of **1** in CD_3OD.

Figure S14: ROSEY (500 MHz) spectrum of **1** in CD_3OD.

Figure S15: HRESIMS of **1**.

Figure S16: ^{1}H NMR (500 MHz) spectrum of **2** in CD_3OD.

Figure S17: ^{13}C NMR (125MHz) spectrum of **2** in CD_3OD.

Figure S18: ^{1}H-^{1}H COSY (500 MHz) spectrum of **2** in CD_3OD

Figure S19: HSQC (500 MHz) spectrum of **2** in CD_3OD.

Figure S20: HSBC (500 MHz) spectrum of **2** in CD_3OD.

Figure S21: ROSEY (500 MHz) spectrum of **2** in CD_3OD.

Figure S22: HRESIMS of **2**.

Figure S23: ^{1}H NMR (500 MHz) spectrum of **3** in CD_3OD.

Figure S24: ^{13}C NMR and DEPT (150 MHz) spectra of **3** in CD_3OD.

Figure S25: ^{1}H-^{1}H COSY (600 MHz) spectrum of **3** in CD_3OD.

Figure S26: HSQC (600 MHz) spectrum of **3** in CD_3OD.

Figure S27: HSBC (600 MHz) spectrum of **3** in CD_3OD.

Figure S28: ROSEY (600 MHz) spectrum of **3** in CD_3OD.

Figure S29: HRESIMS of **3**.

Figure S30: ^{1}H NMR (500 MHz) spectrum of **4** in CD_3OD.

Figure S31: ^{13}C NMR and DEPT (150 MHz) spectra of **4** in CD_3OD.

Figure S32: ^{1}H-^{1}H COSY (600 MHz) spectrum of **4** in CD_3OD.

Figure S33: HSQC (600 MHz) spectrum of **4** in CD_3OD.

Figure S34: HSBC (600 MHz) spectrum of **4** in CD_3OD.

Figure S35: ROSEY (600 MHz) spectrum of **4** in CD_3OD.

Figure S36: HRESIMS of **4**

Figure S37: ^{1}H NMR (500 MHz) spectrum of **5** in $CDCl_3$.

Figure S38: ^{13}C NMR and DEPT (150 MHz) spectra of **5** in $CDCl_3$。

Figure S39: ^{1}H-^{1}H COSY (600 MHz) spectrum of **5** in $CDCl_3$.

Figure S40: HSQC (600 MHz) spectrum of **5** in $CDCl_3$.

Figure S41: HSBC (600 MHz) spectrum of **5** in $CDCl_3$

Figure S42: ROSEY (600 MHz) spectrum of **5** in $CDCl_3$.

Figure S43: HRESIMS of **5**.

Figure S44: UV-vis spectra of **1**–**5**

Fig A: human breast cancer cell line (MCF-7) were treated with 40 μM with conpounds **1**–**5** for 24 h, and cell viability was determined by CCK-8 assay.

Fig B: RAW 264.7 were treated with 20 μM with conpounds **1**–**5** for 24 h, and cell viability was determined by CCK-8 assay.

Fig C: Compounds suppress LPS-induced INOS and COX-2 expression in RAW 264.7 cells.

Selected conformation of **1** and their percentage

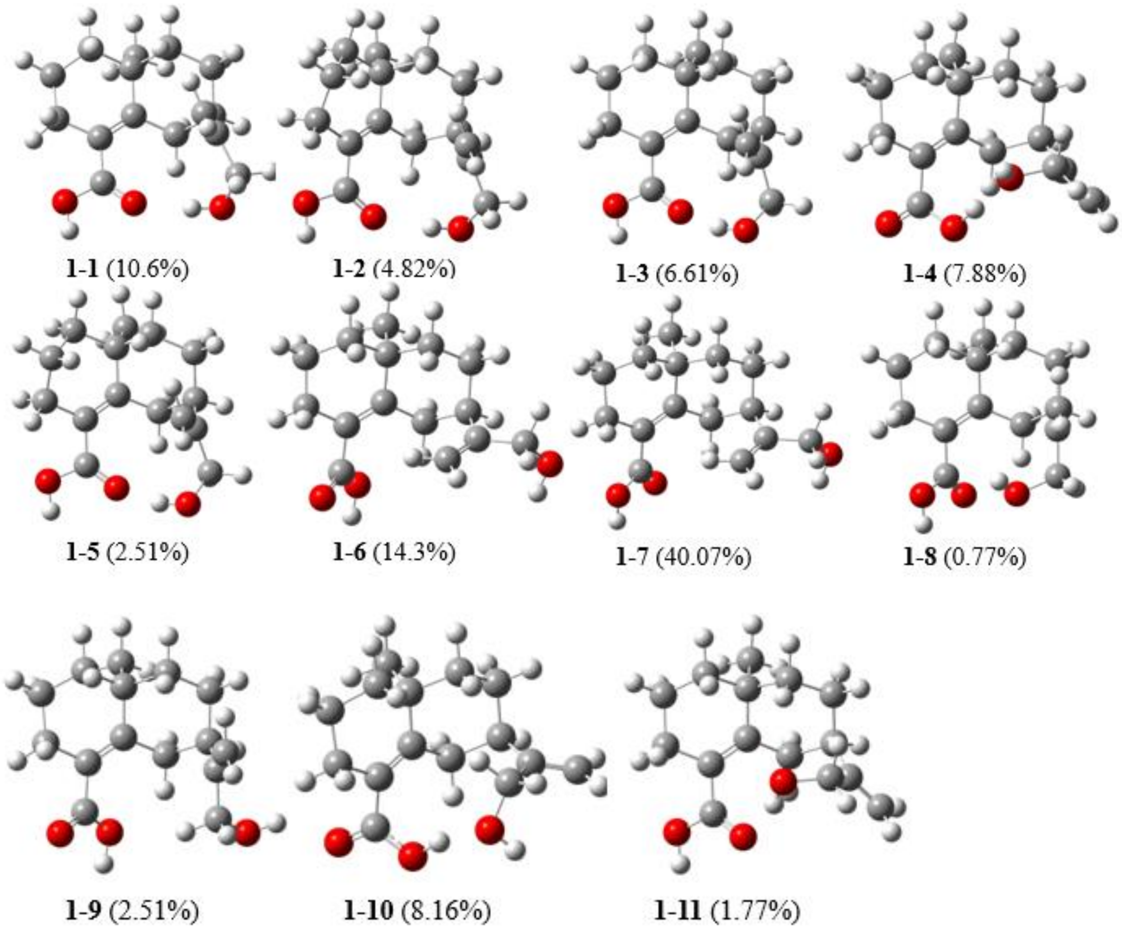

Figure S1: The lowest energy conformers of **1** (the relative populations are in parentheses).

Selected conformation of **2** and their percentage

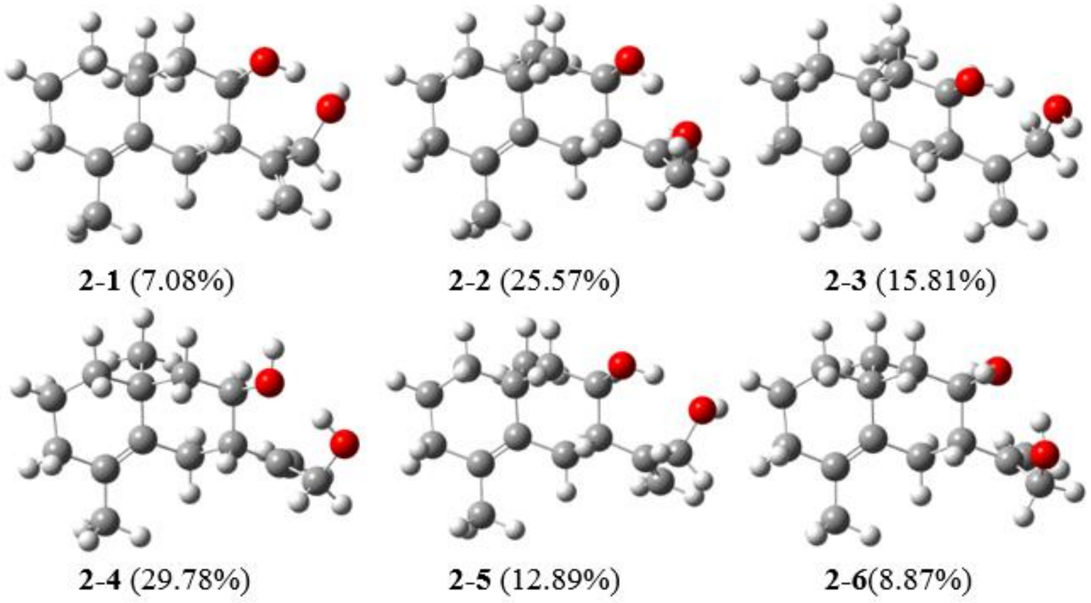

Figure S2: The lowest energy conformers of **2** (the relative populations are in parentheses).

Selected conformation of **3** and their percentage

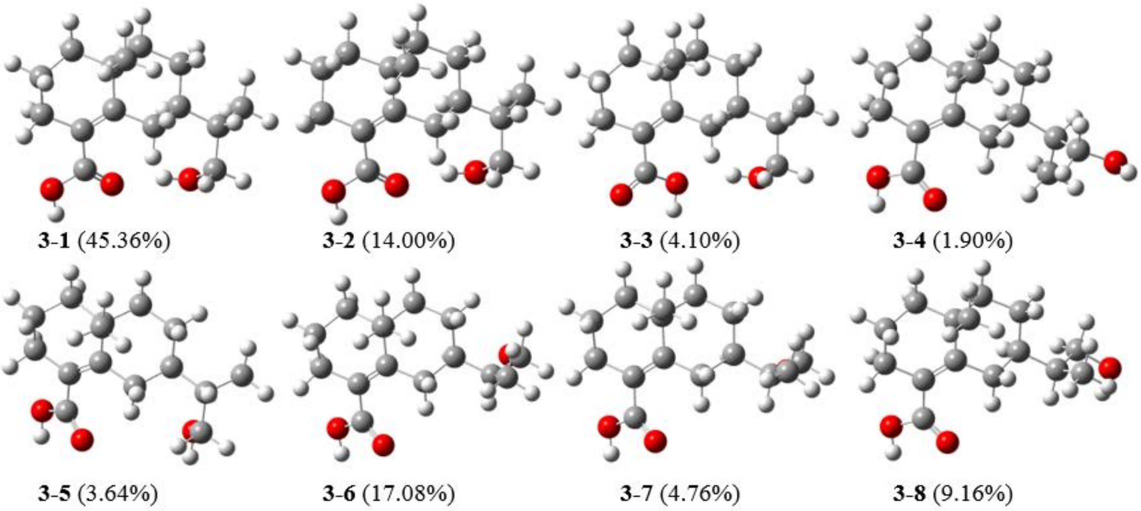

Figure S3: The lowest energy conformers of **3** (the relative populations are in parentheses).

Selected conformation of **4** and their percentage

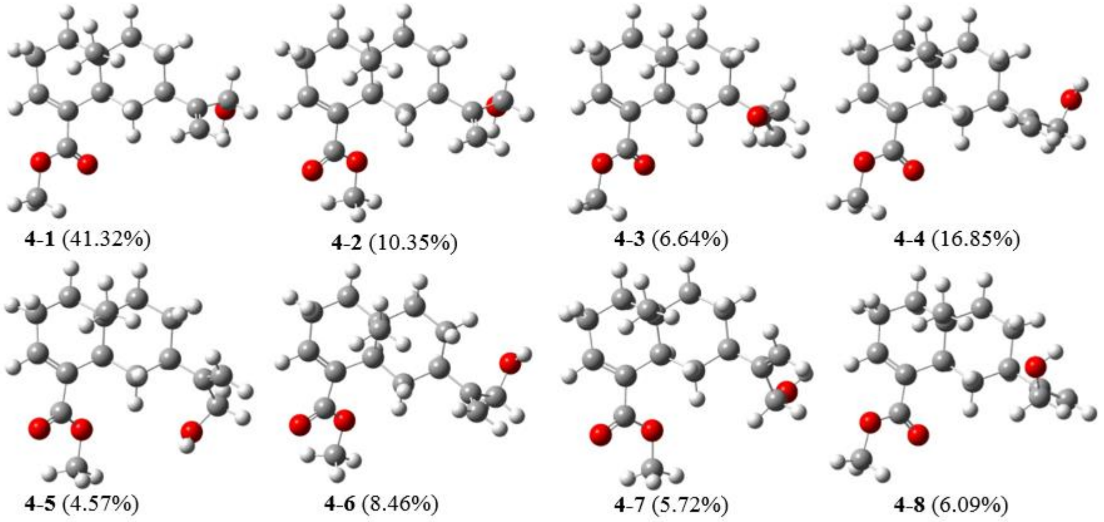

Figure S4: The lowest energy conformers of **4** (the relative populations are in parentheses).

Selected conformation of **5** and their percentage

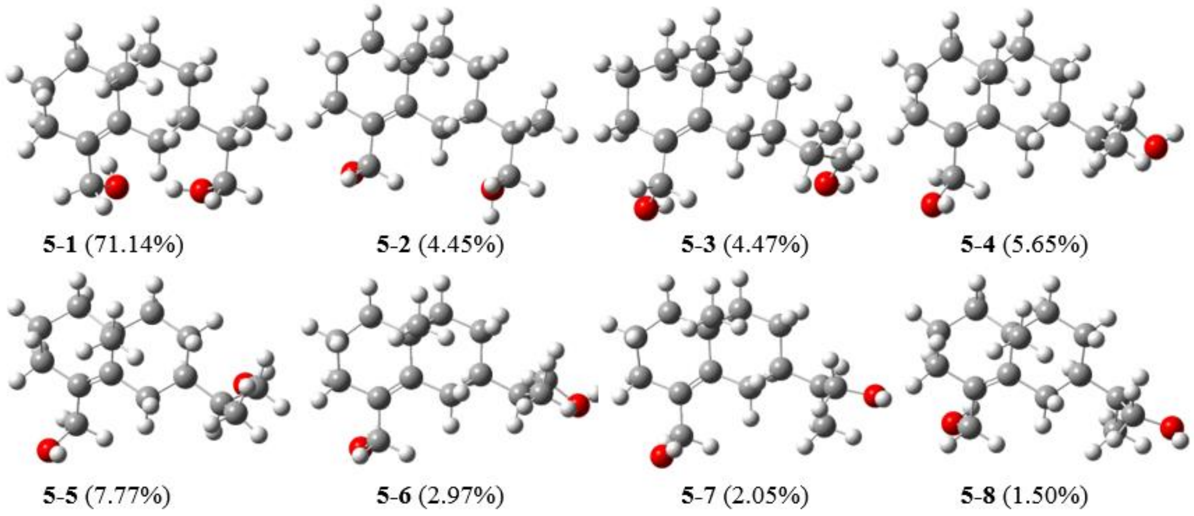

Figure S5: The lowest energy conformers of **5** (the relative populations are in parentheses).

Table S1: Extracted heats and weighting factors of the optimized conformers of **1–5** at B3LYP/6-31G(d,p) level.

		B3LYP/6-31G(d,p)	
	Conformer	Extracted heats	Boltzmann-calculated contribution (%)
1	1	-810.42627	10.6
	2	-810.425526	4.82
	3	-810.425823	6.61
	4	-810.42599	7.88
	5	-810.424909	2.51
	6	-810.426552	14.3
	7	-810.427525	40.07
	8	-810.423792	0.77
	9	-810.424911	2.51
	10	-810.426023	8.16
	11	-810.424577	1.77
2	1	-736.181953	7.08
	2	-736.183165	25.57
	3	-736.182711	15.81
	4	-736.183309	29.78
	5	-736.182518	12.89
	6	-736.182165	8.87
3	1	-811.452558	45.36
	2	-811.451448	14.00
	3	-811.450288	4.10
	4	-811.449562	1.90
	5	-811.450176	3.64
	6	-811.451636	17.08
	7	-811.45043	4.76
	8	-811.451047	9.16
4	1	-849.51583	41.32
	2	-849.514523	10.35
	3	-849.514104	6.64
	4	-849.514983	16.85
	5	-849.513751	4.57
	6	-849.514333	8.46
	7	-849.513963	5.72
	8	-849.514023	6.09
5	1	-737.384987	71.14
	2	-737.38237	4.45
	3	-737.382375	4.47
	4	-737.382596	5.65

	5	-737.382896	7.77
	6	-737.381988	2.97
	7	-737.381638	2.05
	8	-737.381342	1.5

Table S2. The Cartesian coordinates of the lowest energy conformers for **1–5**

**1-1**	X axis(Å)	Y axis(Å)	Z axis(Å)	**1-2**	X axis(Å)	Y axis(Å)	Z axis(Å)
C	-1.3963	-1.4589	-0.5995	C	-1.1888	-1.6092	-0.4689
C	-0.5173	-2.701	-0.3208	C	-0.1583	-2.6951	-0.0635
C	0.8191	-2.425	0.3812	C	1.1886	-2.1799	0.4746
C	1.6341	-1.2305	-0.1806	C	1.8169	-1.0318	-0.3549
C	0.705	-0.0307	-0.4031	C	0.7293	0.0357	-0.5441
C	-0.5323	-0.3481	-1.2251	C	-0.5008	-0.4797	-1.2678
C	2.7577	-0.9124	0.8489	C	3.0766	-0.4883	0.3789
C	3.3461	0.4785	0.6543	C	2.749	0.5582	1.4342
C	2.2608	1.538	0.8198	C	2.0205	1.7328	0.7914
C	0.9895	1.1966	0.0916	C	0.8471	1.2852	-0.0365
C	-2.2859	-0.9581	0.546	C	-2.0918	-1.0843	0.6558
C	-2.0438	-1.1379	1.8568	C	-1.7544	-1.0252	1.9567
C	-3.5769	-0.281	0.1309	C	-3.4896	-0.6732	0.2416
C	2.2979	-1.634	-1.5175	C	2.319	-1.5842	-1.7108
O	-3.3213	0.9454	-0.5273	O	-3.4539	0.4631	-0.6015
C	0.0252	2.3085	-0.0357	C	-0.2281	2.2944	-0.1656
O	-1.1235	2.3199	0.3702	O	-1.4098	2.1522	0.0906
O	0.5678	3.3554	-0.6765	O	0.2672	3.4634	-0.6052
H	-2.0994	-1.789	-1.3792	H	-1.8701	-2.1193	-1.1664
H	-1.0827	-3.4472	0.2512	H	-0.5988	-3.3884	0.6639
H	-0.295	-3.1777	-1.2852	H	0.0493	-3.3048	-0.9531
H	0.627	-2.2654	1.4465	H	1.0628	-1.8569	1.5124
H	1.4278	-3.3385	0.3396	H	1.8853	-3.0277	0.5214
H	-1.1257	0.5407	-1.445	H	-1.2006	0.3176	-1.5211
H	-0.204	-0.6831	-2.2177	H	-0.201	-0.8688	-2.2482
H	2.3638	-0.9699	1.8729	H	3.6345	-1.3128	0.8406
H	3.5563	-1.6624	0.7898	H	3.7624	-0.0278	-0.3462
H	3.8067	0.5629	-0.337	H	3.6722	0.9127	1.9066
H	4.1426	0.6532	1.3868	H	2.1337	0.1197	2.2289
H	2.6661	2.4962	0.4748	H	2.7127	2.2983	0.1552
H	2.0279	1.6563	1.8857	H	1.6904	2.4082	1.5902
H	-2.7339	-0.7711	2.6123	H	-2.4525	-0.6563	2.7036

H	-1.1652	-1.6378	2.2367	H	-0.7849	-1.3211	2.331
H	-4.1441	-0.9276	-0.5467	H	-3.9836	-1.4899	-0.2948
H	-4.2246	-0.0698	0.9887	H	-4.1186	-0.4287	1.1045
H	3.0373	-2.4288	-1.3648	H	3.1584	-2.2741	-1.5623
H	1.5745	-2.0148	-2.2455	H	1.5534	-2.1385	-2.2611
H	2.8135	-0.7872	-1.9838	H	2.6671	-0.7727	-2.3608
H	-2.6516	1.4237	0.0085	H	-2.8303	1.1008	-0.1901
H	-0.1615	4.0069	-0.7261	H	-0.5188	4.0439	-0.669
**1-3**	X axis(Å)	Y axis(Å)	Z axis(Å)	**1-4**	X axis(Å)	Y axis(Å)	Z axis(Å)
C	-1.307	-1.5306	-0.7969	C	-1.8802	-0.7691	-0.9117
C	-0.4232	-2.7431	-0.4303	C	-1.5885	-2.1926	-0.4017
C	0.7957	-2.4054	0.4362	C	-0.345	-2.3143	0.4828
C	1.6531	-1.2176	-0.0725	C	0.9279	-1.6412	-0.0901
C	0.7399	-0.0351	-0.4252	C	0.6002	-0.202	-0.5014
C	-0.4157	-0.3951	-1.3458	C	-0.6068	-0.0584	-1.4116
C	2.6583	-0.8609	1.0604	C	2.0156	-1.6936	1.0218
C	3.2659	0.5227	0.8806	C	3.1573	-0.719	0.7631
C	2.1709	1.5842	0.8849	C	2.6299	0.7107	0.7019
C	0.977	1.2128	0.045	C	1.361	0.8412	-0.0943
C	-2.3004	-1.0368	0.2589	C	-2.7488	0.1006	0.0033
C	-2.3176	-1.4097	1.552	C	-3.7884	0.7807	-0.5196
C	-3.39	-0.1143	-0.2511	C	-2.4864	0.1759	1.4803
C	2.4572	-1.6461	-1.3218	C	1.4409	-2.4311	-1.3159
O	-3.5003	1.0296	0.5809	O	-1.271	0.8781	1.7043
C	0.0354	2.3212	-0.2204	C	1.0228	2.2453	-0.4303
O	-1.1633	2.3197	-0.0042	O	1.75	2.9765	-1.0804
O	0.6601	3.3839	-0.7507	O	-0.1348	2.6399	0.1357
H	-1.9356	-1.8762	-1.6316	H	-2.4887	-0.9283	-1.8166
H	-1.0209	-3.5194	0.063	H	-2.4584	-2.5939	0.1329
H	-0.0654	-3.1999	-1.3625	H	-1.4529	-2.8477	-1.2722
H	0.4533	-2.1918	1.4543	H	-0.5713	-1.888	1.4663
H	1.4217	-3.3035	0.523	H	-0.1499	-3.3796	0.6648
H	-1.0211	0.4739	-1.6147	H	-0.8391	0.9859	-1.6445
H	0.007	-0.7222	-2.305	H	-0.3333	-0.4955	-2.3817
H	2.1555	-0.8841	2.037	H	1.5738	-1.4465	1.9971
H	3.4579	-1.6104	1.1138	H	2.4151	-2.7109	1.1187
H	3.8342	0.5723	-0.0554	H	3.6696	-0.9705	-0.1727
H	3.9765	0.7268	1.6898	H	3.9053	-0.7993	1.5602
H	2.6152	2.5267	0.5447	H	3.4162	1.3511	0.2846
H	1.8292	1.7482	1.9149	H	2.4353	1.0675	1.7214
H	-3.0784	-1.02	2.2255	H	-4.4323	1.4059	0.0926
H	-1.61	-2.1001	1.9888	H	-4.0173	0.7513	-1.5813

H	-3.2104	0.2252	-1.2751	H	-3.2815	0.719	2.0035
H	-4.3513	-0.6396	-0.2508	H	-2.4313	-0.8189	1.9292
H	3.1709	-2.4409	-1.0755	H	1.7292	-3.4501	-1.0327
H	1.8171	-2.0338	-2.1204	H	0.6859	-2.5219	-2.1031
H	3.0257	-0.8094	-1.7426	H	2.3167	-1.9523	-1.7674
H	-2.6437	1.5028	0.5013	H	-1.1415	0.8692	2.6716
H	-0.0547	4.0401	-0.8827	H	-0.5778	1.9137	0.6434
**1-5**	X axis(Å)	Y axis(Å)	Z axis(Å)	**1-6**	X axis(Å)	Y axis(Å)	Z axis(Å)
C	-0.9711	-1.7554	-0.6651	C	1.8218	-0.2982	0.6513
C	0.1144	-2.739	-0.1634	C	1.7048	-1.8248	0.5104
C	1.32	-2.0875	0.5347	C	0.5265	-2.2341	-0.3711
C	1.9327	-0.8921	-0.237	C	-0.8393	-1.6852	0.1148
C	0.7818	0.0755	-0.5559	C	-0.7607	-0.1738	0.3689
C	-0.3204	-0.5524	-1.3912	C	0.4738	0.2974	1.1296
C	3.0551	-0.242	0.6225	C	-1.8853	-2.0361	-0.9809
C	2.5279	0.7797	1.6183	C	-3.1912	-1.2718	-0.81
C	1.7844	1.8848	0.878	C	-2.9399	0.2306	-0.854
C	0.7455	1.3365	-0.0634	C	-1.7367	0.6625	-0.0608
C	-2.0181	-1.3068	0.3592	C	2.4584	0.3476	-0.5882
C	-1.899	-1.4174	1.6952	C	1.7965	1.0658	-1.5135
C	-3.2987	-0.7484	-0.2301	C	3.9522	0.1599	-0.7268
C	2.6247	-1.3978	-1.526	C	-1.2481	-2.386	1.4325
O	-3.6867	0.4338	0.4529	O	4.6076	0.8593	0.3203
C	-0.381	2.2603	-0.3265	C	-1.7193	2.1169	0.2031
O	-1.5705	2.0187	-0.2364	O	-1.82	2.9908	-0.6379
O	0.0694	3.475	-0.6822	O	-1.6702	2.3912	1.5166
H	-1.5396	-2.3184	-1.4208	H	2.523	-0.1069	1.4764
H	-0.3265	-3.4948	0.4983	H	2.6242	-2.257	0.0999
H	0.4865	-3.3006	-1.0304	H	1.593	-2.2698	1.5064
H	1.0232	-1.7614	1.537	H	0.7246	-1.891	-1.3952
H	2.0844	-2.86	0.6924	H	0.4887	-3.3302	-0.4223
H	-1.0771	0.1732	-1.6988	H	0.5703	1.3883	1.1278
H	0.1052	-0.897	-2.3414	H	0.3316	0.017	2.1822
H	3.624	-1.0144	1.1553	H	-1.4816	-1.8009	-1.9755
H	3.7776	0.2693	-0.0292	H	-2.0897	-3.1142	-0.9814
H	3.3605	1.2124	2.1847	H	-3.6731	-1.5436	0.136
H	1.8647	0.2974	2.3463	H	-3.8924	-1.549	-1.6057
H	2.4953	2.4974	0.3097	H	-3.8395	0.7423	-0.49
H	1.3175	2.5395	1.6242	H	-2.7968	0.5413	-1.8969
H	-2.7031	-1.085	2.3486	H	2.3092	1.5056	-2.3649
H	-1.0305	-1.8319	2.1875	H	0.7289	1.2429	-1.4726
H	-3.1976	-0.5036	-1.2914	H	4.2315	-0.8956	-0.6721

H	-4.1031	-1.4862	-0.1409	H	4.3319	0.546	-1.679
H	3.4894	-2.0263	-1.2814	H	-1.331	-3.4697	1.2907
H	1.9697	-2	-2.1617	H	-0.5252	-2.219	2.2373
H	2.985	-0.5585	-2.1328	H	-2.2147	-2.0262	1.8016
H	-2.9446	1.0643	0.3251	H	4.2495	1.7635	0.3124
H	-0.7455	3.9979	-0.8287	H	-1.705	3.3686	1.5533
**1-7**	X axis(Å)	Y axis(Å)	Z axis(Å)	**1-8**	X axis(Å)	Y axis(Å)	Z axis(Å)
C	1.8293	-0.3432	0.6339	C	1.3444	-1.528	0.7593
C	1.7029	-1.8617	0.429	C	0.4149	-2.7337	0.4954
C	0.5018	-2.2304	-0.44	C	-0.7998	-2.4008	-0.3778
C	-0.8488	-1.6948	0.0999	C	-1.6244	-1.1807	0.1031
C	-0.7564	-0.1923	0.4002	C	-0.6923	-0.0052	0.4193
C	0.4937	0.2415	1.159	C	0.5003	-0.3499	1.2984
C	-1.926	-2.0068	-0.9765	C	-2.6369	-0.8313	-1.0268
C	-3.2229	-1.2446	-0.7411	C	-3.2341	0.5618	-0.8672
C	-2.9657	0.2575	-0.7494	C	-2.133	1.6168	-0.8847
C	-1.7338	0.6644	0.0153	C	-0.9475	1.2393	-0.0451
C	2.4506	0.3496	-0.5878	C	2.3112	-1.1381	-0.3707
C	1.7737	1.0863	-1.4872	C	2.3313	-1.6843	-1.6009
C	3.9457	0.1828	-0.7403	C	3.395	-0.1443	-0.0071
C	-1.2249	-2.4384	1.4043	C	-2.4203	-1.5556	1.3754
O	4.6021	0.8514	0.326	O	2.9251	1.1842	-0.1567
C	-1.688	2.1001	0.3737	C	-0.0648	2.367	0.2974
O	-1.4899	2.5756	1.4755	O	0.009	2.9284	1.3729
O	-1.9082	2.8733	-0.7027	O	0.6949	2.7019	-0.7588
H	2.5446	-0.1913	1.4552	H	1.9998	-1.8459	1.5844
H	2.6106	-2.2774	-0.0229	H	0.9749	-3.5641	0.0484
H	1.6144	-2.3497	1.407	H	0.0577	-3.1139	1.4613
H	0.677	-1.8477	-1.4542	H	-0.4597	-2.2236	-1.4039
H	0.4568	-3.3233	-0.5333	H	-1.4455	-3.2873	-0.4321
H	0.6001	1.33	1.1999	H	1.1429	0.5127	1.4912
H	0.3671	-0.0794	2.2019	H	0.116	-0.6255	2.2896
H	-1.55	-1.7401	-1.974	H	-2.1409	-0.8753	-2.0063
H	-2.1346	-3.0837	-1.0066	H	-3.4431	-1.5748	-1.0609
H	-3.6763	-1.5423	0.2111	H	-3.8031	0.6283	0.0673
H	-3.95	-1.4956	-1.5222	H	-3.9432	0.7594	-1.6794
H	-3.8503	0.7624	-0.3422	H	-2.5563	2.5687	-0.5419
H	-2.8565	0.5927	-1.7889	H	-1.7952	1.7705	-1.9174
H	2.2759	1.5576	-2.3279	H	3.0716	-1.3836	-2.3385
H	0.7038	1.2438	-1.4346	H	1.6369	-2.4489	-1.9256
H	4.2352	-0.8713	-0.7241	H	3.7325	-0.2869	1.0244
H	4.3152	0.6059	-1.6807	H	4.276	-0.2597	-0.6481

H	-1.3187	-3.5161	1.2278	H	-3.1384	-2.3572	1.1667
H	-0.4791	-2.3037	2.194	H	-1.7741	-1.9099	2.1846
H	-2.1783	-2.0862	1.8129	H	-2.9831	-0.7013	1.7673
H	4.2086	1.7393	0.3798	H	2.2493	1.1743	-0.8607
H	-1.8525	3.7832	-0.3446	H	1.3049	3.3748	-0.386
**1-9**	X axis(Å)	Y axis(Å)	Z axis(Å)	**1-10**	X axis(Å)	Y axis(Å)	Z axis(Å)
C	1.6314	-1.1012	0.6224	C	-1.9793	-0.4815	-0.9318
C	0.9696	-2.4938	0.536	C	-1.8844	-1.9812	-0.6058
C	-0.3348	-2.49	-0.2679	C	-0.775	-2.3239	0.3848
C	-1.3726	-1.4326	0.1932	C	0.6145	-1.7578	-0.0015
C	-0.7001	-0.0664	0.3949	C	0.5413	-0.2725	-0.3903
C	0.5924	-0.1087	1.1957	C	-0.6015	0.1013	-1.3212
C	-2.4861	-1.3796	-0.8926	C	1.5569	-1.9816	1.2132
C	-3.3577	-0.1363	-0.7761	C	2.8863	-1.254	1.0622
C	-2.5067	1.1208	-0.9158	C	2.67	0.2447	0.8867
C	-1.2386	1.0687	-0.11	C	1.5012	0.5986	0.0055
C	2.3411	-0.5917	-0.6466	C	-2.7782	0.29	0.1239
C	2.4906	-1.3015	-1.7811	C	-4.0928	0.5174	-0.0671
C	2.9594	0.7875	-0.5706	C	-2.1189	0.7779	1.3824
C	-2.0115	-1.88	1.5293	C	1.1647	-2.5516	-1.2132
O	3.868	0.8334	0.5195	O	-1.4594	2.0053	1.1033
C	-0.6264	2.3983	0.0843	C	1.5845	1.9836	-0.5194
O	-0.2827	3.158	-0.8022	O	2.4558	2.3474	-1.292
O	-0.5622	2.7346	1.3825	O	0.657	2.8046	-0.0017
H	2.4262	-1.1962	1.3766	H	-2.5749	-0.4212	-1.8563
H	1.6638	-3.2314	0.1164	H	-2.8416	-2.3524	-0.2179
H	0.7578	-2.8456	1.5539	H	-1.7124	-2.5329	-1.5386
H	-0.0944	-2.3253	-1.3246	H	-1.0687	-1.9723	1.3796
H	-0.7798	-3.4926	-0.2202	H	-0.7153	-3.4169	0.4755
H	1.0509	0.8758	1.311	H	-0.7006	1.1835	-1.4506
H	0.3458	-0.413	2.2216	H	-0.3422	-0.2839	-2.317
H	-2.0371	-1.3834	-1.8954	H	1.0735	-1.6251	2.1334
H	-3.1161	-2.2763	-0.8384	H	1.7427	-3.0532	1.3589
H	-3.8859	-0.1281	0.1843	H	3.4445	-1.6524	0.2074
H	-4.1277	-0.1457	-1.5562	H	3.5108	-1.4321	1.9456
H	-3.1136	1.984	-0.616	H	3.5981	0.6826	0.4997
H	-2.2436	1.2663	-1.9714	H	2.4965	0.6969	1.8719
H	3.0148	-0.8916	-2.6407	H	-4.6959	1.0518	0.6617
H	2.1116	-2.3088	-1.9059	H	-4.6092	0.171	-0.9585
H	3.5162	1.0438	-1.4787	H	-2.8463	0.9598	2.1816
H	2.2011	1.5619	-0.4348	H	-1.3882	0.0743	1.7794
H	-2.5526	-2.8256	1.4084	H	1.2981	-3.6099	-0.9611

H	-1.2677	-2.0369	2.3168	H	0.495	-2.5074	-2.0782
H	-2.7238	-1.1377	1.9063	H	2.134	-2.167	-1.5488
H	4.4936	0.1014	0.3835	H	-2.1616	2.6112	0.7918
H	-0.1597	3.6267	1.3751	H	-0.0108	2.3397	0.5608
**1-11**	X axis(Å)	Y axis(Å)	Z axis(Å)				
C	-1.9252	-0.6804	-0.9093				
C	-1.6901	-2.1245	-0.4318				
C	-0.4507	-2.313	0.4455				
C	0.8494	-1.682	-0.1177				
C	0.583	-0.2217	-0.5018				
C	-0.6252	-0.0122	-1.3978				
C	1.9325	-1.8065	0.9931				
C	3.1064	-0.8638	0.7681				
C	2.6251	0.5831	0.7536				
C	1.378	0.7827	-0.0628				
C	-2.7566	0.208	0.0206				
C	-3.7545	0.9567	-0.4899				
C	-2.5034	0.2274	1.5052				
C	1.3283	-2.4679	-1.3594				
O	-1.2425	0.7958	1.7981				
C	1.0178	2.1929	-0.3084				
O	-0.0237	2.7335	0.0216				
O	1.9945	2.8484	-0.9533				
H	-2.541	-0.7964	-1.8159				
H	-2.5741	-2.5026	0.0966				
H	-1.5819	-2.7656	-1.3165				
H	-0.66	-1.8959	1.437				
H	-0.299	-3.3885	0.6076				
H	-0.8176	1.0439	-1.6113				
H	-0.3741	-0.4397	-2.3783				
H	1.4976	-1.5758	1.9754				
H	2.2971	-2.8393	1.0585				
H	3.6123	-1.1014	-0.1749				
H	3.8489	-0.9947	1.5637				
H	3.4423	1.21	0.3782				
H	2.4213	0.9078	1.782				
H	-4.3712	1.5929	0.1388				
H	-3.9774	0.9672	-1.5527				
H	-3.2578	0.8212	2.0337				
H	-2.5538	-0.779	1.9291				
H	1.5678	-3.5053	-1.0985				
H	0.5721	-2.5058	-2.1498				

H	2.2272	-2.0207	-1.7977				
H	-1.1354	1.5763	1.2166				
H	1.6404	3.7549	-1.0583				
**2-1**	X axis(Å)	Y axis(Å)	Z axis(Å)	**2-2**	X axis(Å)	Y axis(Å)	Z axis(Å)
C	-3.8776	-0.7045	0.0523	C	-3.9174	-0.5331	-0.2083
C	-3.699	0.7758	-0.2565	C	-3.595	0.8762	-0.6873
C	-2.3052	1.3122	0.0044	C	-2.2171	1.3704	-0.2945
C	-1.2418	0.5013	0.2295	C	-1.2558	0.541	0.1808
C	-1.3523	-1.0342	0.1641	C	-1.4562	-0.9807	0.2827
C	-2.6914	-1.4961	-0.4724	C	-2.735	-1.453	-0.4625
C	0.1536	1.0082	0.5698	C	0.0995	1.0115	0.6822
C	1.2226	0.4443	-0.3844	C	1.2773	0.2975	-0.0145
C	1.181	-1.0995	-0.3465	C	1.1246	-1.2389	0.096
C	-0.213	-1.6114	-0.7156	C	-0.2575	-1.7023	-0.3767
C	2.5939	1.0594	-0.0844	C	2.6379	0.7833	0.5108
C	3.0542	2.0896	-0.819	C	2.9162	0.9564	1.8165
C	3.4115	0.4957	1.0424	C	3.6801	1.0743	-0.5348
O	3.9646	-0.7394	0.6084	O	4.0428	-0.1577	-1.1399
C	-2.2619	2.8206	-0.0227	C	-2.0649	2.8586	-0.4903
O	2.114	-1.6523	-1.2768	O	2.0704	-1.9327	-0.726
C	-1.2617	-1.6163	1.5946	C	-1.5721	-1.3967	1.7674
H	-3.9919	-0.852	1.1321	H	-4.1717	-0.5194	0.8577
H	-4.8033	-1.0661	-0.4103	H	-4.8028	-0.906	-0.7361
H	-4.4291	1.339	0.3381	H	-4.3591	1.5548	-0.2884
H	-3.942	0.9544	-1.3119	H	-3.6744	0.9138	-1.7814
H	-2.8533	-2.5661	-0.291	H	-2.995	-2.4776	-0.1684
H	-2.6554	-1.3742	-1.5638	H	-2.5552	-1.4847	-1.546
H	0.2119	2.0993	0.5636	H	0.2337	2.0908	0.5732
H	0.3832	0.7222	1.6043	H	0.125	0.8356	1.7641
H	0.9548	0.7443	-1.4095	H	1.2296	0.5635	-1.081
H	1.449	-1.4794	0.6455	H	1.297	-1.5669	1.1272
H	-0.395	-1.3703	-1.7731	H	-0.3	-1.5665	-1.4678
H	-0.2168	-2.7091	-0.6719	H	-0.3401	-2.7879	-0.2273
H	4.0268	2.5351	-0.6328	H	3.8932	1.2974	2.1477
H	2.4764	2.5049	-1.6399	H	2.1965	0.7385	2.5987
H	4.235	1.1629	1.3192	H	3.2857	1.7525	-1.2985
H	2.7985	0.3266	1.9329	H	4.5807	1.5264	-0.1063
H	4.5731	-1.0301	1.3099	H	4.7079	0.0539	-1.8186
H	-1.264	3.2332	-0.178	H	-1.0277	3.193	-0.5454
H	-2.8729	3.1995	-0.8501	H	-2.5271	3.1625	-1.4365
H	-2.6655	3.2242	0.9114	H	-2.5638	3.3973	0.3215
H	2.9972	-1.3905	-0.9369	H	2.9003	-1.4082	-0.7384

H	-1.3822	-2.7057	1.5823	H	-1.7764	-2.4698	1.8589
H	-0.3007	-1.4074	2.0745	H	-0.6555	-1.2005	2.3323
H	-2.0374	-1.2045	2.2493	H	-2.3814	-0.8605	2.275
**2-3**	X axis(Å)	Y axis(Å)	Z axis(Å)	**2-4**	X axis(Å)	Y axis(Å)	Z axis(Å)
C	-3.8915	-0.693	0.0342	C	-3.9195	-0.5837	-0.2007
C	-3.7014	0.7818	-0.2938	C	-3.621	0.8253	-0.696
C	-2.3079	1.3144	-0.0241	C	-2.2503	1.3464	-0.3128
C	-1.2518	0.5013	0.2259	C	-1.2752	0.5409	0.175
C	-1.3697	-1.0339	0.181	C	-1.4523	-0.9814	0.3021
C	-2.7034	-1.4982	-0.4649	C	-2.7203	-1.486	-0.4399
C	0.1415	1.0055	0.5772	C	0.0753	1.0439	0.6623
C	1.2208	0.4263	-0.3571	C	1.2545	0.3393	-0.0409
C	1.1697	-1.1178	-0.2989	C	1.1301	-1.1912	0.1292
C	-0.223	-1.627	-0.6776	C	-0.2386	-1.6975	-0.3388
C	2.5865	1.0479	-0.0416	C	2.6273	0.8598	0.4108
C	3.0164	2.1329	-0.7127	C	2.9125	1.2392	1.6706
C	3.4279	0.4292	1.036	C	3.6796	0.9803	-0.6686
O	4.0955	-0.6937	0.4742	O	4.2499	-0.2849	-0.9527
C	-2.2556	2.822	-0.0719	C	-2.1203	2.8335	-0.5351
O	2.1073	-1.689	-1.2128	O	2.1278	-1.8525	-0.663
C	-1.2984	-1.5971	1.62	C	-1.5682	-1.3721	1.7937
H	-4.0193	-0.8242	1.1146	H	-4.1775	-0.5613	0.8642
H	-4.8136	-1.0565	-0.4341	H	-4.7966	-0.9787	-0.7262
H	-4.4354	1.3573	0.2838	H	-4.395	1.4962	-0.3033
H	-3.9309	0.9463	-1.3546	H	-3.7034	0.8486	-1.7903
H	-2.8732	-2.5646	-0.2701	H	-2.963	-2.511	-0.1326
H	-2.6539	-1.3922	-1.5575	H	-2.5368	-1.5289	-1.5224
H	0.2029	2.0963	0.5604	H	0.1881	2.1241	0.5403
H	0.3591	0.7305	1.6175	H	0.1158	0.8766	1.7453
H	0.9714	0.7151	-1.39	H	1.1678	0.569	-1.1134
H	1.4255	-1.4871	0.7004	H	1.3077	-1.4728	1.1736
H	-0.3914	-1.3984	-1.7402	H	-0.2787	-1.5905	-1.433
H	-0.2328	-2.7241	-0.6204	H	-0.3009	-2.78	-0.1609
H	3.9778	2.5933	-0.5052	H	3.9012	1.6069	1.9339
H	2.4149	2.5904	-1.4938	H	2.1963	1.1742	2.4816
H	4.1807	1.1215	1.4288	H	3.2528	1.394	-1.5881
H	2.8169	0.099	1.8813	H	4.492	1.65	-0.3668
H	4.7805	-0.3263	-0.1144	H	3.5123	-0.9319	-0.9583
H	-1.2542	3.226	-0.228	H	-1.0881	3.1801	-0.6077
H	-2.8597	3.1932	-0.9079	H	-2.5965	3.1155	-1.4812
H	-2.6618	3.2411	0.8541	H	-2.6178	3.3791	0.2729
H	2.9843	-1.5403	-0.8016	H	2.0432	-2.8079	-0.4884

H	-1.4245	-2.686	1.621	H	-1.7564	-2.4464	1.9039
H	-0.3419	-1.3868	2.1081	H	-0.6573	-1.1512	2.3587
H	-2.0794	-1.1725	2.2601	H	-2.3878	-0.8394	2.2884
**2-5**	X axis(Å)	Y axis(Å)	Z axis(Å)	**2-6**	X axis(Å)	Y axis(Å)	Z axis(Å)
C	-3.9291	-0.6661	-0.0327	C	-3.897	-0.5156	-0.2752
C	-3.694	0.7212	-0.6159	C	-3.5606	0.9115	-0.6866
C	-2.3188	1.2924	-0.3338	C	-2.1889	1.3847	-0.2479
C	-1.2994	0.5333	0.1377	C	-1.2397	0.5356	0.217
C	-1.4284	-0.9825	0.3444	C	-1.4501	-0.9893	0.2608
C	-2.7237	-1.5529	-0.2966	C	-2.7098	-1.4279	-0.5352
C	0.0585	1.0862	0.5338	C	0.1116	0.9867	0.7533
C	1.2436	0.3958	-0.183	C	1.2803	0.2873	0.0326
C	1.1513	-1.1405	0.0214	C	1.1264	-1.2473	0.141
C	-0.2364	-1.6851	-0.3409	C	-0.2368	-1.7038	-0.3866
C	2.6139	0.9871	0.2207	C	2.6537	0.7585	0.5191
C	2.8288	1.7843	1.2855	C	2.984	0.8679	1.8189
C	3.7659	0.6847	-0.7026	C	3.6465	1.1061	-0.5613
O	4.3159	-0.5681	-0.3136	O	3.8829	-0.0341	-1.3734
C	-2.2378	2.7689	-0.6333	C	-2.0279	2.8786	-0.3891
O	2.0557	-1.8505	-0.8312	O	2.136	-1.9286	-0.6139
C	-1.4427	-1.3127	1.8546	C	-1.6122	-1.4493	1.7285
H	-4.1249	-0.5939	1.0433	H	-4.1806	-0.5452	0.783
H	-4.8252	-1.1068	-0.4847	H	-4.7685	-0.8643	-0.8413
H	-4.4631	1.3939	-0.2165	H	-4.3303	1.5757	-0.2744
H	-3.8346	0.6851	-1.7038	H	-3.6188	0.9953	-1.7794
H	-2.9226	-2.5661	0.0743	H	-2.979	-2.4626	-0.2884
H	-2.602	-1.6468	-1.3846	H	-2.5015	-1.417	-1.6139
H	0.1538	0.9627	1.62	H	0.2518	2.0682	0.6818
H	0.125	2.1641	0.3645	H	0.1346	0.7707	1.8283
H	1.1316	0.6005	-1.2583	H	1.2076	0.5607	-1.031
H	1.4002	-1.4045	1.0557	H	1.2481	-1.5705	1.1808
H	-0.347	-1.6144	-1.4337	H	-0.2477	-1.5445	-1.4749
H	-0.2613	-2.7632	-0.1285	H	-0.3241	-2.7917	-0.2611
H	3.8148	2.1899	1.4986	H	3.9696	1.2054	2.1283
H	2.0495	2.0644	1.9856	H	2.2934	0.6095	2.6153
H	3.4388	0.6237	-1.7452	H	3.2591	1.9112	-1.1937
H	4.5557	1.4424	-0.6525	H	4.6069	1.4359	-0.1517
H	4.7284	-0.4225	0.5571	H	4.009	-0.7843	-0.7589
H	-1.2201	3.1311	-0.7886	H	-0.9888	3.2107	-0.4148
H	-2.779	2.9957	-1.5591	H	-2.4724	3.2172	-1.332
H	-2.6956	3.343	0.1785	H	-2.5391	3.3907	0.4321
H	2.9677	-1.5785	-0.5956	H	2.2003	-1.4814	-1.4807

H	-1.5973	-2.3854	2.0197	H	-1.8188	-2.5245	1.7815
H	-0.5044	-1.0494	2.3525	H	-0.7146	-1.269	2.3279
H	-2.2436	-0.7779	2.3769	H	-2.4371	-0.9283	2.2266
**3-1**	X axis(Å)	Y axis(Å)	Z axis(Å)	**3-2**	X axis(Å)	Y axis(Å)	Z axis(Å)
C	3.6311	-0.5554	-0.3088	C	3.1718	-0.4694	-1.2252
C	3.0041	0.8216	-0.5124	C	2.8989	0.8853	-0.58
C	1.5812	0.9077	-0.0232	C	1.5412	0.9426	0.0663
C	0.844	-0.1763	0.3188	C	0.8709	-0.1609	0.4756
C	1.3396	-1.6111	0.0932	C	1.4276	-1.5794	0.3003
C	2.6579	-1.647	-0.7311	C	2.887	-1.5899	-0.2351
C	-0.5552	-0.0981	0.8982	C	-0.5539	-0.1366	0.9875
C	-1.5598	-0.7406	-0.0697	C	-1.465	-0.7412	-0.0968
C	-1.1771	-2.2285	-0.2228	C	-1.0104	-2.1949	-0.3724
C	0.2676	-2.3949	-0.7165	C	0.4901	-2.2951	-0.7111
C	-3.0299	-0.53	0.3939	C	-2.9692	-0.6305	0.2842
C	-3.3956	0.9575	0.5861	C	-3.4148	0.8212	0.5602
O	-3.0859	1.7326	-0.5638	O	-3.0938	1.6888	-0.5182
C	1.5893	-2.3055	1.4503	C	1.4533	-2.3603	1.6348
C	-4.0229	-1.1555	-0.5979	C	-3.8694	-1.2231	-0.8106
C	0.9824	2.2665	-0.011	C	0.9082	2.2845	0.0815
O	-0.1517	2.5626	-0.3485	O	-0.245	2.5539	-0.2074
O	1.8515	3.1954	0.4162	O	1.7846	3.2312	0.4543
H	4.5528	-0.6278	-0.8975	H	2.5561	-0.59	-2.1245
H	3.9181	-0.686	0.7412	H	4.2177	-0.5168	-1.5496
H	3.0193	1.0725	-1.5807	H	2.9895	1.6537	-1.3574
H	3.6392	1.5544	-0.0019	H	3.663	1.0999	0.1773
H	2.444	-1.5125	-1.8002	H	3.1141	-2.5571	-0.7011
H	3.1412	-2.6279	-0.641	H	3.5919	-1.483	0.6014
H	-0.5831	-0.6145	1.8656	H	-0.6403	-0.7014	1.9222
H	-0.828	0.9266	1.1451	H	-0.8609	0.8713	1.2649
H	-1.4482	-0.2681	-1.0564	H	-1.3153	-0.1782	-1.0298
H	-1.3089	-2.7516	0.7319	H	-1.5666	-2.6143	-1.2178
H	-1.8321	-2.7237	-0.947	H	-1.2376	-2.8254	0.4954
H	0.5184	-3.4633	-0.7355	H	0.7628	-3.3551	-0.7922
H	0.2871	-2.0606	-1.7634	H	0.6326	-1.8672	-1.7124
H	-3.1725	-1.0305	1.3608	H	-3.1449	-1.2078	1.2015
H	-4.4695	1.0619	0.776	H	-4.5001	0.8619	0.7051
H	-2.8901	1.3932	1.4536	H	-2.9699	1.2175	1.4781
H	-2.1324	1.9577	-0.5324	H	-2.1492	1.9399	-0.4376
H	0.67	-2.4386	2.029	H	0.4566	-2.5578	2.0392
H	2.0175	-3.3043	1.3068	H	1.9364	-3.3361	1.504
H	2.2852	-1.733	2.0735	H	2.0146	-1.8114	2.4002

H	-5.0558	-0.9236	-0.3157	H	-4.9274	-1.0588	-0.578
H	-3.9404	-2.2462	-0.6122	H	-3.7323	-2.305	-0.8976
H	-3.8592	-0.7842	-1.6151	H	-3.6636	-0.7702	-1.7861
H	1.3443	4.032	0.3727	H	1.265	4.0608	0.4258
**3-3**	X axis(Å)	Y axis(Å)	Z axis(Å)	**3-4**	X axis(Å)	Y axis(Å)	Z axis(Å)
C	3.6501	-0.5565	-0.2827	C	3.5561	-1.2164	0.2204
C	3.0367	0.827	-0.4762	C	3.342	0.2583	-0.0987
C	1.6037	0.9198	-0.0176	C	1.9129	0.7062	0.0654
C	0.8509	-0.1593	0.3045	C	0.8567	-0.1394	0.1425
C	1.3462	-1.5957	0.0843	C	1.0425	-1.664	0.0673
C	2.6713	-1.6342	-0.7284	C	2.4638	-2.0578	-0.4246
C	-0.5491	-0.0755	0.8791	C	-0.5856	0.3208	0.3104
C	-1.5561	-0.743	-0.0733	C	-1.5251	-0.3044	-0.7387
C	-1.1609	-2.2275	-0.2249	C	-1.4152	-1.8372	-0.7058
C	0.2807	-2.3814	-0.7283	C	0.0291	-2.2844	-0.9289
C	-3.0265	-0.5538	0.4028	C	-3.0043	0.1609	-0.6176
C	-3.4099	0.9247	0.6233	C	-3.6213	-0.0695	0.7698
O	-3.1298	1.7271	-0.5165	O	-5.0191	0.1964	0.7207
C	1.5861	-2.2865	1.4458	C	0.8265	-2.2647	1.4768
C	-4.0194	-1.1747	-0.5922	C	-3.1576	1.6344	-1.0262
C	1.1107	2.3165	0.0579	C	1.7503	2.1718	0.2049
O	1.7298	3.2656	0.5025	O	1.1263	2.7619	1.0665
O	-0.0911	2.4792	-0.5291	O	2.4135	2.8266	-0.7626
H	4.5771	-0.6299	-0.863	H	4.5383	-1.5323	-0.1499
H	3.926	-0.7017	0.7682	H	3.5682	-1.3688	1.3057
H	3.076	1.0928	-1.5404	H	3.6514	0.4485	-1.1345
H	3.6605	1.5539	0.0577	H	4.0028	0.85	0.5468
H	2.4674	-1.4855	-1.7978	H	2.5356	-1.9307	-1.5136
H	3.1448	-2.6204	-0.645	H	2.6554	-3.1204	-0.2291
H	-0.5758	-0.5702	1.8578	H	-0.9112	0.0587	1.3234
H	-0.8237	0.9561	1.0974	H	-0.6718	1.4062	0.2324
H	-1.4601	-0.2779	-1.065	H	-1.171	0.0153	-1.7304
H	-1.2826	-2.7494	0.7317	H	-1.783	-2.2394	0.2434
H	-1.8161	-2.7303	-0.9436	H	-2.044	-2.2708	-1.493
H	0.5386	-3.4479	-0.7538	H	0.0753	-3.3799	-0.8792
H	0.2952	-2.0404	-1.7731	H	0.3083	-2.013	-1.9564
H	-3.1563	-1.0713	1.3625	H	-3.5957	-0.4123	-1.3455
H	-4.4811	1.0129	0.8354	H	-3.4961	-1.1049	1.0968
H	-2.8919	1.3523	1.4873	H	-3.1773	0.5927	1.5199
H	-2.1606	1.7668	-0.6266	H	-5.3648	0.0716	1.6211
H	0.6635	-2.4151	2.0202	H	-0.1847	-2.0891	1.8573
H	2.0129	-3.2868	1.3081	H	0.9826	-3.3497	1.4687

H	2.279	-1.7138	2.0722	H	1.5187	-1.8365	2.2101
H	-5.0528	-0.959	-0.2991	H	-4.2147	1.9094	-1.1059
H	-3.9249	-2.2639	-0.6252	H	-2.6986	1.8185	-2.0032
H	-3.8672	-0.785	-1.6042	H	-2.6947	2.3082	-0.2984
H	-0.2338	3.4484	-0.4676	H	2.2279	3.7689	-0.5713
**3-5**	X axis(Å)	Y axis(Å)	Z axis(Å)	**3-6**	X axis(Å)	Y axis(Å)	Z axis(Å)
C	3.5907	-0.9467	-0.181	C	3.375	-1.4711	-0.3348
C	3.1681	0.4942	-0.4399	C	3.2547	0.0334	-0.5463
C	1.7501	0.7875	-0.0225	C	1.9545	0.6085	-0.0462
C	0.82	-0.1675	0.2244	C	0.8584	-0.1359	0.2388
C	1.1506	-1.6598	0.0921	C	0.8571	-1.6642	0.0671
C	2.4998	-1.9029	-0.6411	C	2.0839	-2.1638	-0.7468
C	-0.6093	0.1344	0.6532	C	-0.4532	0.4438	0.7495
C	-1.6842	-0.6	-0.1794	C	-1.6651	-0.0176	-0.0866
C	-1.3695	-2.1054	-0.243	C	-1.718	-1.5513	-0.1431
C	0.0523	-2.3701	-0.7333	C	-0.4118	-2.1213	-0.6978
C	-3.1227	-0.3413	0.3779	C	-2.9899	0.6399	0.4071
C	-3.466	1.1532	0.5152	C	-4.1707	0.3872	-0.546
O	-3.2992	1.8205	-0.7275	O	-3.8622	0.802	-1.8696
C	1.2398	-2.2845	1.5043	C	0.8928	-2.3268	1.4649
C	-4.1949	-1.0166	-0.492	C	-3.3976	0.2366	1.8275
C	1.4612	2.2291	0.1639	C	1.9853	2.0733	0.1755
O	0.9254	2.7548	1.1217	O	1.6063	2.677	1.1614
O	1.8966	2.949	-0.8839	O	2.5232	2.7117	-0.8773
H	4.5229	-1.1573	-0.7179	H	4.2119	-1.8583	-0.9273
H	3.8043	-1.091	0.8842	H	3.609	-1.6864	0.714
H	3.2692	0.7096	-1.5114	H	3.3446	0.2518	-1.6182
H	3.8649	1.1572	0.0877	H	4.1024	0.5196	-0.0478
H	2.3704	-1.7745	-1.7247	H	1.9269	-1.98	-1.8187
H	2.8333	-2.9381	-0.4957	H	2.1988	-3.2496	-0.6382
H	-0.7196	-0.1267	1.713	H	-0.5686	0.1475	1.7977
H	-0.8094	1.2043	0.5777	H	-0.4495	1.5368	0.7395
H	-1.6363	-0.2089	-1.2058	H	-1.4904	0.3408	-1.1117
H	-1.5246	-2.5672	0.7388	H	-1.9132	-1.9725	0.8485
H	-2.0535	-2.6078	-0.9356	H	-2.5367	-1.8816	-0.7915
H	0.2296	-3.4534	-0.7397	H	-0.4742	-3.2171	-0.7009
H	0.1098	-2.0449	-1.7815	H	-0.3365	-1.8169	-1.7511
H	-3.1909	-0.7813	1.3819	H	-2.8143	1.725	0.411
H	-4.5052	1.2818	0.8376	H	-4.4459	-0.6714	-0.5763
H	-2.834	1.6387	1.2649	H	-5.056	0.944	-0.2193
H	-3.4779	2.7632	-0.5691	H	-3.6345	1.7468	-1.8343
H	0.2985	-2.2081	2.0577	H	0.0128	-2.0818	2.0679

H	1.4931	-3.3494	1.4465	H	0.9295	-3.4191	1.3802
H	2.0057	-1.7949	2.1157	H	1.7692	-2.0101	2.041
H	-5.201	-0.7443	-0.1542	H	-4.3004	0.7776	2.133
H	-4.1301	-2.107	-0.432	H	-2.6196	0.4781	2.5567
H	-4.1003	-0.7236	-1.5428	H	-3.6174	-0.833	1.8987
H	1.6425	3.8659	-0.6537	H	2.489	3.6547	-0.616
**3-7**	X axis(Å)	Y axis(Å)	Z axis(Å)	**3-8**	X axis(Å)	Y axis(Å)	Z axis(Å)
C	3.3776	-1.4709	-0.342	C	3.5555	-1.3215	-0.1976
C	3.2575	0.0333	-0.5564	C	3.3696	0.1683	-0.4592
C	1.9595	0.6083	-0.0529	C	2.015	0.6856	-0.0482
C	0.8638	-0.1339	0.2378	C	0.9446	-0.1083	0.1981
C	0.8605	-1.6619	0.0706	C	1.0321	-1.6384	0.0696
C	2.0842	-2.1643	-0.7465	C	2.3275	-2.0926	-0.6603
C	-0.4453	0.4514	0.7493	C	-0.4209	0.4144	0.6218
C	-1.6607	-0.0121	-0.0804	C	-1.5576	-0.1305	-0.2672
C	-1.7152	-1.5461	-0.1306	C	-1.5287	-1.6659	-0.2867
C	-0.4117	-2.1199	-0.6881	C	-0.1659	-2.1795	-0.7528
C	-2.9834	0.6486	0.4158	C	-2.9369	0.4681	0.1324
C	-4.1695	0.3916	-0.5286	C	-4.0012	0.178	-0.9398
O	-3.8542	0.8212	-1.8454	O	-5.1949	0.9069	-0.6778
C	0.9009	-2.3202	1.4702	C	1.0216	-2.2634	1.4849
C	-3.3834	0.2539	1.8409	C	-3.4383	0.0175	1.5108
C	1.9893	2.0726	0.1657	C	1.9576	2.155	0.1325
O	1.649	2.6728	1.1677	O	1.5022	2.7625	1.0828
O	2.4794	2.7123	-0.9093	O	2.5062	2.7931	-0.915
H	4.2118	-1.8601	-0.9372	H	4.4437	-1.6789	-0.7313
H	3.6161	-1.6839	0.7063	H	3.7402	-1.4965	0.8684
H	3.3436	0.2496	-1.629	H	3.5083	0.3632	-1.5304
H	4.1068	0.5208	-0.062	H	4.1612	0.7122	0.0708
H	1.923	-1.9839	-1.8183	H	2.2224	-1.9454	-1.7441
H	2.1991	-3.2499	-0.6349	H	2.4922	-3.1675	-0.5142
H	-0.5576	0.16	1.7992	H	-0.581	0.1396	1.67
H	-0.4381	1.5447	0.7346	H	-0.4716	1.5056	0.5816
H	-1.4893	0.3417	-1.1075	H	-1.3498	0.2141	-1.2915
H	-1.9071	-1.9632	0.8634	H	-1.7566	-2.0737	0.7036
H	-2.5365	-1.8783	-0.7746	H	-2.2931	-2.0533	-0.9691
H	-0.4756	-3.2157	-0.6871	H	-0.1718	-3.2769	-0.7303
H	-0.3399	-1.8193	-1.7427	H	-0.0463	-1.8964	-1.8079
H	-2.8074	1.7336	0.4109	H	-2.8232	1.5598	0.1869
H	-4.4398	-0.6684	-0.5598	H	-3.6461	0.4646	-1.9351
**4-1**	X axis(Å)	Y axis(Å)	Z axis(Å)	**4-2**	X axis(Å)	Y axis(Å)	Z axis(Å)

C	-2.8328	2.3204	-0.3816	C	3.1734	-1.9746	-0.4265
C	-2.97	0.8263	-0.3176	C	3.1453	-0.5095	-0.0984
C	-1.9228	-0.0239	-0.3179	C	2.0138	0.2202	-0.0501
C	-0.4874	0.4622	-0.4042	C	0.6457	-0.3733	-0.3128
C	-0.346	1.937	0.1035	C	0.634	-1.9205	-0.0727
C	-1.3985	2.7979	-0.6374	C	1.8139	-2.5335	-0.8679
C	0.5763	-0.4306	0.2715	C	-0.5338	0.2855	0.4337
C	2.0064	0.0687	-0.0513	C	-1.8856	-0.2777	-0.0693
C	2.1857	1.5518	0.3207	C	-1.932	-1.8143	0.0226
C	1.0734	2.4405	-0.245	C	-0.7039	-2.4834	-0.6046
C	3.1322	-0.7775	0.5482	C	-3.1207	0.3377	0.5914
C	2.9993	-1.5561	1.6378	C	-3.1334	0.8726	1.8261
C	4.4602	-0.7089	-0.1695	C	-4.3835	0.321	-0.237
O	4.322	-1.2588	-1.4704	O	-4.2159	1.1744	-1.3587
C	-0.5703	2.0826	1.6325	C	0.7858	-2.3116	1.4218
H	-0.2546	0.4553	-1.4811	H	0.4666	-0.2013	-1.3863
C	-2.1967	-1.4832	-0.3657	C	2.1802	1.668	0.2126
O	-1.5888	-2.2639	-1.0813	O	2.8502	2.1308	1.1221
O	-3.1802	-1.8064	0.513	O	1.5137	2.3706	-0.737
C	-3.4894	-3.1993	0.5442	C	1.6355	3.7854	-0.5959
H	-3.4831	2.7094	-1.1737	H	3.9067	-2.1535	-1.2214
H	-3.2018	2.7425	0.5607	H	3.529	-2.5201	0.4556
H	-3.9896	0.4464	-0.2905	H	4.1087	-0.0403	0.0967
H	-1.2042	2.7592	-1.7182	H	1.6794	-2.3229	-1.9379
H	-1.3113	3.8533	-0.3502	H	1.8229	-3.6264	-0.7715
H	0.4974	-1.4629	-0.0866	H	-0.5394	1.3689	0.2686
H	0.3963	-0.4667	1.3514	H	-0.4139	0.1433	1.5131
H	2.1009	-0.0055	-1.1444	H	-1.9313	-0.0154	-1.1364
H	3.1438	1.924	-0.0622	H	-2.0325	-2.1259	1.0692
H	2.236	1.6635	1.4104	H	-2.8246	-2.1954	-0.4887
H	1.1889	2.485	-1.3363	H	-0.7533	-2.3445	-1.6929
H	1.2132	3.4659	0.1196	H	-0.757	-3.5656	-0.4313
H	3.8309	-2.1417	2.0208	H	-4.039	1.2982	2.2504
H	2.0705	-1.6378	2.1914	H	-2.2563	0.8988	2.4634
H	4.8096	0.3234	-0.2601	H	-4.6107	-0.6876	-0.5938
H	5.2409	-1.2727	0.3524	H	-5.2538	0.6719	0.3277
H	3.8324	-2.0935	-1.3696	H	-3.8786	2.0192	-1.0147
H	-0.5371	3.1388	1.9248	H	0.8511	-3.4011	1.5268
H	-1.5367	1.6855	1.9567	H	1.6834	-1.8861	1.8801
H	0.1898	1.5659	2.2241	H	-0.0581	-1.9844	2.0346
H	-3.8444	-3.536	-0.4349	H	1.2204	4.1127	0.3625
H	-2.6169	-3.779	0.8614	H	2.682	4.092	-0.6909
H	-4.2899	-3.3524	1.2734	H	1.0627	4.2565	-1.3994

**4-3**	X axis(Å)	Y axis(Å)	Z axis(Å)	**4-4**	X axis(Å)	Y axis(Å)	Z axis(Å)
C	-2.7987	2.3179	0.1526	C	-2.7428	2.4212	-0.2863
C	-2.8931	0.8244	0.0284	C	-2.9565	0.9351	-0.2579
C	-1.8446	0.0291	-0.2667	C	-1.9558	0.0325	-0.3128
C	-0.4559	0.5839	-0.5228	C	-0.4998	0.4449	-0.4286
C	-0.2575	1.9833	0.1535	C	-0.2654	1.8971	0.1102
C	-1.4399	2.8883	-0.2716	C	-1.2937	2.8307	-0.5744
C	0.7243	-0.3469	-0.1685	C	0.5314	-0.523	0.1913
C	2.063	0.2603	-0.6396	C	1.9745	-0.078	-0.1389
C	2.281	1.6866	-0.1054	C	2.2468	1.3851	0.2492
C	1.0706	2.5919	-0.3567	C	1.1673	2.3375	-0.2727
C	3.2649	-0.6365	-0.3565	C	3.024	-1.0129	0.4534
C	4.0822	-1.0198	-1.3552	C	3.0875	-1.308	1.7646
C	3.5408	-1.0668	1.0619	C	3.9959	-1.6158	-0.5268
O	2.6614	-2.1193	1.4242	O	4.802	-0.5839	-1.0741
C	-0.2168	1.9116	1.7035	C	-0.4348	2.011	1.6488
H	-0.412	0.732	-1.6138	H	-0.2974	0.4546	-1.5116
C	-2.0911	-1.4213	-0.4711	C	-2.3072	-1.4086	-0.3922
O	-1.6097	-2.0665	-1.3897	O	-1.7676	-2.1964	-1.1532
O	-2.8896	-1.9059	0.514	O	-3.2736	-1.7074	0.5136
C	-3.1455	-3.3059	0.4113	C	-3.6523	-3.0834	0.5178
H	-3.5837	2.7814	-0.4562	H	-3.0609	2.8352	0.6778
H	-3.0078	2.5926	1.1933	H	-3.3952	2.8654	-1.0471
H	-3.8843	0.3989	0.174	H	-3.9933	0.6073	-0.2103
H	-1.4345	3.0038	-1.3643	H	-1.1343	2.8119	-1.6615
H	-1.326	3.8983	0.1418	H	-1.1429	3.8719	-0.2628
H	0.61	-1.3212	-0.6562	H	0.3987	-1.5349	-0.2079
H	0.731	-0.5438	0.9087	H	0.3708	-0.6042	1.2722
H	1.9763	0.3588	-1.7328	H	2.0618	-0.133	-1.2342
H	3.1589	2.1315	-0.5915	H	3.217	1.7003	-0.1558
H	2.5085	1.6725	0.9658	H	2.3318	1.4872	1.3374
H	0.9971	2.7808	-1.436	H	1.2542	2.3942	-1.3659
H	1.2477	3.5672	0.1138	H	1.3679	3.3484	0.1038
H	4.9477	-1.651	-1.1748	H	3.8392	-1.9846	2.1609
H	3.9091	-0.7154	-2.3836	H	2.3933	-0.8801	2.4813
H	4.5664	-1.4334	1.1794	H	4.6567	-2.3519	-0.057
H	3.4129	-0.2422	1.768	H	3.4644	-2.1189	-1.3408
H	2.6078	-2.7163	0.6578	H	5.1544	-0.0812	-0.3201
H	-0.1476	2.9189	2.1309	H	-0.3265	3.0539	1.9691
H	-1.1076	1.4388	2.1274	H	-1.4144	1.6654	1.992
H	0.6402	1.3471	2.0801	H	0.3076	1.4296	2.2016
H	-3.6687	-3.5329	-0.523	H	-4.0604	-3.3725	-0.4558

H	-2.212	-3.8732	0.4812	H	-2.7993	-3.7153	0.7846
H	-3.789	-3.5952	1.2468	H	-4.4317	-3.218	1.273
**4-5**	X axis(Å)	Y axis(Å)	Z axis(Å)	**4-6**	X axis(Å)	Y axis(Å)	Z axis(Å)
C	3.3342	-1.6803	-0.3604	C	3.0164	-2.1923	-0.3565
C	3.1267	-0.2398	0.0092	C	3.1147	-0.7237	-0.0594
C	1.9207	0.3605	0.0134	C	2.0534	0.1061	-0.0491
C	0.6423	-0.3677	-0.3448	C	0.642	-0.3673	-0.3266
C	0.7881	-1.9156	-0.1563	C	0.4854	-1.9022	-0.0576
C	2.0713	-2.3664	-0.8983	C	1.6206	-2.6361	-0.8147
C	-0.6395	0.1347	0.3521	C	-0.4853	0.4136	0.3815
C	-1.8881	-0.5694	-0.2235	C	-1.8717	-0.0512	-0.1197
C	-1.7719	-2.1041	-0.1989	C	-2.0611	-1.5744	-0.0108
C	-0.4473	-2.6006	-0.7873	C	-0.8869	-2.3556	-0.6093
C	-3.1714	-0.0895	0.4453	C	-3.0173	0.6996	0.5511
C	-3.4189	-0.2572	1.7571	C	-3.1737	0.757	1.8861
C	-4.172	0.5847	-0.4553	C	-3.9765	1.4017	-0.374
O	-3.6171	1.7999	-0.9345	O	-4.6581	0.4347	-1.1573
C	0.898	-2.3425	1.3319	C	0.5714	-2.2712	1.4476
H	0.5023	-0.1764	-1.4208	H	0.4982	-0.2033	-1.4067
C	1.9115	1.8082	0.3255	C	2.3462	1.5385	0.1872
O	2.4731	2.3097	1.2864	O	3.0441	1.9581	1.0967
O	1.2275	2.4653	-0.6435	O	1.7585	2.2779	-0.786
C	1.1812	3.8794	-0.4578	C	2.0033	3.6791	-0.6694
H	4.1258	-1.7497	-1.1155	H	3.7454	-2.4543	-1.132
H	3.6983	-2.2159	0.5243	H	3.3044	-2.7478	0.5439
H	4.0204	0.3244	0.2728	H	4.1129	-0.339	0.1454
H	1.9739	-2.1321	-1.9674	H	1.5263	-2.4382	-1.8914
H	2.1957	-3.4547	-0.8352	H	1.5291	-3.723	-0.6957
H	-0.7628	1.2134	0.2006	H	-0.399	1.4871	0.1774
H	-0.5638	-0.0062	1.4365	H	-0.3925	0.3089	1.4684
H	-1.9383	-0.285	-1.285	H	-1.8993	0.1921	-1.1923
H	-2.5986	-2.5427	-0.7727	H	-2.9801	-1.8704	-0.5327
H	-1.8829	-2.4874	0.8219	H	-2.2045	-1.8718	1.0345
H	-0.4572	-2.4156	-1.8697	H	-0.9049	-2.2271	-1.6998
H	-0.3843	-3.6889	-0.6626	H	-1.0365	-3.4267	-0.4242
H	-4.3425	0.0883	2.2128	H	-3.9936	1.305	2.3417
H	-2.7065	-0.7467	2.4146	H	-2.4908	0.2544	2.5645
H	-4.4155	-0.054	-1.3103	H	-4.7238	1.9892	0.1696
H	-5.1077	0.8178	0.0635	H	-3.4404	2.0831	-1.0423
H	-3.2855	2.2778	-0.1551	H	-5.0212	-0.2178	-0.5344
H	1.0647	-3.4235	1.408	H	0.5254	-3.3589	1.5776
H	1.7234	-1.8484	1.8526	H	1.499	-1.9267	1.914

H	-0.0051	-2.119	1.9057	H	-0.2455	-1.846	2.0364
H	0.6778	4.1267	0.4821	H	1.6069	4.0605	0.2769
H	2.1903	4.3027	-0.4809	H	3.0738	3.8901	-0.7553
H	0.606	4.3107	-1.2817	H	1.4844	4.183	-1.4895
**4-7**	X axis(Å)	Y axis(Å)	Z axis(Å)	**4-8**	X axis(Å)	Y axis(Å)	Z axis(Å)
C	3.2764	-1.7939	-0.4479	C	-2.6381	2.5024	0.0302
C	3.1472	-0.3479	-0.0637	C	-2.9021	1.0241	0.0493
C	1.97	0.3051	-0.0093	C	-1.9609	0.091	-0.2008
C	0.6485	-0.3644	-0.3233	C	-0.5304	0.4589	-0.5491
C	0.7323	-1.9168	-0.1407	C	-0.1516	1.8808	-0.0122
C	1.9634	-2.4231	-0.9335	C	-1.242	2.8738	-0.484
C	-0.5894	0.1965	0.4092	C	0.5538	-0.5672	-0.1508
C	-1.8871	-0.4468	-0.1289	C	1.9339	-0.1551	-0.71
C	-1.831	-1.9869	-0.0779	C	2.3249	1.2772	-0.3108
C	-0.5569	-2.5445	-0.722	C	1.214	2.2867	-0.6171
C	-3.1106	0.1692	0.5486	C	3.0072	-1.1876	-0.3769
C	-3.7058	-0.3387	1.643	C	3.5128	-1.983	-1.3381
C	-3.6355	1.436	-0.083	C	3.4648	-1.3327	1.0517
O	-4.1809	1.128	-1.3566	O	4.4069	-0.3159	1.3546
C	0.8836	-2.3495	1.3423	C	-0.0582	1.9414	1.536
H	0.4835	-0.1662	-1.3947	H	-0.5156	0.5049	-1.65
C	2.0384	1.7491	0.3118	C	-2.3771	-1.3333	-0.2637
O	2.6584	2.217	1.2535	O	-2.007	-2.1089	-1.1312
O	1.3496	2.4447	-0.6267	O	-3.1856	-1.6337	0.7848
C	1.3745	3.8578	-0.4288	C	-3.6113	-2.9955	0.8118
H	4.0315	-1.8928	-1.2363	H	-3.39	2.9955	-0.5968
H	3.6546	-2.3496	0.4183	H	-2.7751	2.8917	1.046
H	4.0745	0.1741	0.1684	H	-3.9282	0.7298	0.2619
H	1.8334	-2.1837	-1.998	H	-1.2669	2.8912	-1.5824
H	2.0421	-3.5159	-0.8747	H	-1.0004	3.8967	-0.1689
H	-0.656	1.2801	0.263	H	0.3124	-1.5565	-0.5555
H	-0.5016	0.0478	1.4922	H	0.5818	-0.6874	0.9378
H	-1.9517	-0.1956	-1.1978	H	1.8226	-0.1375	-1.8052
H	-1.8943	-2.3532	0.9515	H	3.2338	1.575	-0.8489
H	-2.7024	-2.4015	-0.6008	H	2.5711	1.3331	0.7543
H	-0.6027	-2.3592	-1.8034	H	1.1205	2.3817	-1.7071
H	-0.5386	-3.6345	-0.5974	H	1.5148	3.275	-0.2473
H	-4.572	0.1406	2.0915	H	4.2652	-2.7356	-1.1199
H	-3.3482	-1.2332	2.1411	H	3.1838	-1.9103	-2.3709
H	-4.4215	1.9045	0.5187	H	2.6308	-1.2615	1.7548
H	-2.8367	2.1725	-0.2114	H	3.9486	-2.3003	1.2242
H	-4.7801	0.3734	-1.2251	H	5.0239	-0.277	0.604

H	0.9925	-3.4382	1.4132	H	0.1523	2.9654	1.8664
H	1.7579	-1.9035	1.8253	H	-0.9831	1.6251	2.0273
H	0.0217	-2.0747	1.9556	H	0.7353	1.3068	1.939
H	0.9186	4.1188	0.5314	H	-4.1911	-3.2346	-0.0853
H	2.4002	4.2354	-0.4872	H	-2.7505	-3.6648	0.9076
H	0.7888	4.322	-1.227	H	-4.2544	-3.1323	1.6856
**5-1**	X axis(Å)	Y axis(Å)	Z axis(Å)	**5-2**	X axis(Å)	Y axis(Å)	Z axis(Å)
C	3.7578	0.1774	-0.2218	C	3.7389	-0.3445	-0.1466
C	2.949	1.4674	-0.3158	C	3.15	1.0583	-0.2113
C	1.5209	1.3324	0.1618	C	1.7041	1.1508	0.2361
C	0.9482	0.125	0.3897	C	0.9078	0.0575	0.3594
C	1.6311	-1.2063	0.055	C	1.4065	-1.3602	0.038
C	2.9438	-0.9953	-0.7494	C	2.7596	-1.3512	-0.7241
C	-0.455	-0.0224	0.9455	C	-0.5451	0.1249	0.8107
C	-1.357	-0.7259	-0.0805	C	-1.5234	-0.59	-0.1455
C	-0.775	-2.1321	-0.3368	C	-1.0545	-2.0373	-0.3772
C	0.6782	-2.0605	-0.8274	C	0.3886	-2.0885	-0.8736
C	-2.8447	-0.7502	0.3745	C	-2.9946	-0.54	0.3804
C	-3.408	0.6566	0.6688	C	-3.4826	0.8855	0.6988
O	-3.1825	1.5519	-0.4121	O	-3.2847	1.742	-0.4168
C	1.9668	-1.9669	1.357	C	1.5871	-2.1438	1.3588
C	-3.7386	-1.4269	-0.6763	C	-3.9702	-1.1811	-0.62
C	0.8006	2.6508	0.3316	C	1.2788	2.5715	0.522
O	-0.3925	2.655	-0.4509	O	0.9213	3.1961	-0.7005
H	4.6822	0.2823	-0.8013	H	3.9866	-0.6007	0.8899
H	4.0577	-0.0031	0.8171	H	4.6799	-0.3731	-0.708
H	2.9339	1.8126	-1.3573	H	3.2159	1.4344	-1.2402
H	3.4652	2.2368	0.2713	H	3.7783	1.7112	0.4068
H	2.7172	-0.8003	-1.8066	H	2.5987	-1.0996	-1.7815
H	3.555	-1.9063	-0.7342	H	3.2113	-2.3511	-0.7153
H	-0.4253	-0.5961	1.8798	H	-0.6231	-0.3103	1.8147
H	-0.8638	0.9449	1.237	H	-0.8662	1.1619	0.9069
H	-1.3063	-0.1721	-1.0292	H	-1.4868	-0.0701	-1.1137
H	-0.8341	-2.737	0.5758	H	-1.1611	-2.6229	0.5431
H	-1.3555	-2.6576	-1.102	H	-1.6785	-2.5258	-1.1332
H	1.0724	-3.0798	-0.9283	H	0.6834	-3.1382	-1.001
H	0.6525	-1.6435	-1.8441	H	0.4086	-1.6424	-1.8779
H	-2.9245	-1.3364	1.2995	H	-3.0542	-1.1186	1.3119
H	-4.4894	0.6055	0.8369	H	-4.5493	0.8828	0.9492
H	-2.978	1.0848	1.5798	H	-2.9503	1.3021	1.5595
H	-2.2829	1.9278	-0.3086	H	-3.6412	2.6145	-0.1756
H	1.0714	-2.2605	1.9136	H	0.6481	-2.2594	1.9092

H	2.5202	-2.8882	1.1413	H	1.9728	-3.1518	1.1673
H	2.5824	-1.3607	2.0308	H	2.2903	-1.6431	2.0334
H	-4.7949	-1.3581	-0.3937	H	-5.0075	-1.0539	-0.2908
H	-3.5093	-2.4924	-0.7702	H	-3.8017	-2.2581	-0.7098
H	-3.6213	-0.9611	-1.6604	H	-3.8742	-0.7332	-1.6146
H	0.5448	2.8283	1.3809	H	0.4428	2.6526	1.2196
H	1.4144	3.4921	-0.0062	H	2.0969	3.1423	0.9741
H	-0.1327	2.3401	-1.3358	H	0.2929	2.5952	-1.1393
**5-3**	X axis(Å)	Y axis(Å)	Z axis(Å)	**5-4**	X axis(Å)	Y axis(Å)	Z axis(Å)
C	3.6187	-0.9022	-0.3709	C	3.6812	-0.9864	-0.27
C	3.3557	0.5919	-0.2416	C	3.4045	0.4482	-0.7002
C	1.9897	0.944	0.3179	C	2.0232	0.9586	-0.3371
C	0.9787	0.0406	0.3905	C	1.0274	0.1337	0.0747
C	1.1328	-1.4072	-0.1132	C	1.1988	-1.3952	0.1382
C	2.4106	-1.5962	-0.9749	C	2.4863	-1.8689	-0.5894
C	-0.3975	0.3542	0.963	C	-0.3431	0.6186	0.5252
C	-1.5372	-0.0098	-0.0095	C	-1.4782	-0.0608	-0.2634
C	-1.4387	-1.4907	-0.4023	C	-1.3731	-1.5875	-0.1171
C	-0.0708	-1.8008	-1.0102	C	0.0014	-2.0902	-0.5644
C	-2.9313	0.399	0.5574	C	-2.8716	0.5135	0.1197
C	-4.0524	0.2907	-0.4893	C	-3.9578	0.0412	-0.8604
O	-3.7345	1.0748	-1.6307	O	-5.1558	0.7764	-0.6403
C	1.2124	-2.3591	1.1036	C	1.2752	-1.8449	1.6162
C	-3.3433	-0.3686	1.8171	C	-3.2951	0.215	1.5642
C	1.9138	2.3818	0.7742	C	1.9059	2.4553	-0.4962
O	1.8301	3.2188	-0.3693	O	2.5094	3.0858	0.6228
H	4.4992	-1.0657	-1.0032	H	4.5715	-1.3592	-0.7895
H	3.8568	-1.3284	0.6103	H	3.9091	-1.02	0.8015
H	3.4552	1.0635	-1.2276	H	3.5221	0.5266	-1.7887
H	4.1427	1.0157	0.3941	H	4.1716	1.0905	-0.2507
H	2.2528	-1.1882	-1.9829	H	2.3347	-1.857	-1.6775
H	2.6281	-2.6632	-1.1094	H	2.7154	-2.9091	-0.3259
H	-0.5038	-0.1934	1.906	H	-0.4408	0.4094	1.5964
H	-0.5074	1.4102	1.2176	H	-0.4521	1.7017	0.4362
H	-1.3713	0.5835	-0.9204	H	-1.3188	0.1804	-1.3252
H	-1.6118	-2.1377	0.464	H	-1.5544	-1.8929	0.9188
H	-2.2067	-1.7424	-1.1414	H	-2.1355	-2.0827	-0.7284
H	-0.0235	-2.8702	-1.2536	H	0.0525	-3.1754	-0.4071
H	-0.0055	-1.267	-1.9686	H	0.0725	-1.9355	-1.6501
H	-2.8562	1.4618	0.8278	H	-2.8161	1.608	0.0346
H	-4.2123	-0.7443	-0.8067	H	-3.6453	0.2115	-1.8958
H	-4.9942	0.6676	-0.0755	H	-4.1827	-1.0219	-0.73

H	-4.4749	0.9847	-2.2549	H	-5.8112	0.4535	-1.2821
H	0.2992	-2.346	1.7067	H	0.3519	-1.6397	2.1667
H	1.3681	-3.3953	0.7815	H	1.4554	-2.9237	1.6887
H	2.038	-2.093	1.7727	H	2.0843	-1.3369	2.1523
H	-4.2942	0.0154	2.2033	H	-4.2356	0.7223	1.8056
H	-2.6064	-0.2598	2.6174	H	-2.5514	0.5678	2.2839
H	-3.4794	-1.4357	1.6161	H	-3.4504	-0.856	1.7272
H	1.0702	2.605	1.4282	H	2.4263	2.7865	-1.4016
H	2.8118	2.6563	1.3382	H	0.8796	2.8141	-0.5902
H	1.1186	2.8523	-0.923	H	2.173	2.6268	1.4114
**5-5**	X axis(Å)	Y axis(Å)	Z axis(Å)	**5-6**	X axis(Å)	Y axis(Å)	Z axis(Å)
C	3.5254	-1.1044	-0.3933	C	3.7423	-0.8765	-0.1987
C	3.2871	0.349	-0.7815	C	3.4612	0.6198	-0.1856
C	1.9516	0.9088	-0.3315	C	2.061	0.9945	0.2655
C	0.9465	0.1182	0.1223	C	1.0535	0.087	0.339
C	1.0542	-1.4171	0.1474	C	1.2496	-1.3907	-0.0518
C	2.2775	-1.9295	-0.6604	C	2.5768	-1.6227	-0.8236
C	-0.3752	0.6511	0.657	C	-0.3556	0.4208	0.8112
C	-1.583	0.0371	-0.0764	C	-1.4347	-0.0271	-0.1947
C	-1.5343	-1.4954	0.0345	C	-1.2994	-1.5298	-0.4782
C	-0.2095	-2.0455	-0.4993	C	0.1035	-1.8616	-0.9861
C	-2.9286	0.6605	0.4054	C	-2.8596	0.4011	0.259
C	-4.1238	0.2421	-0.4667	C	-3.884	0.2171	-0.8726
O	-3.8817	0.5786	-1.8254	O	-5.1083	0.8498	-0.5193
C	1.193	-1.9009	1.61	C	1.2672	-2.2545	1.2316
C	-3.2567	0.3809	1.8752	C	-3.347	-0.3016	1.5334
C	1.8865	2.4115	-0.461	C	1.9522	2.4584	0.623
O	2.5663	2.9976	0.6388	O	1.9736	3.2228	-0.5734
H	4.3685	-1.5036	-0.969	H	4.6594	-1.0719	-0.7664
H	3.811	-1.1694	0.6628	H	3.9258	-1.2336	0.8211
H	3.3471	0.4444	-1.8733	H	3.6175	1.0254	-1.1933
H	4.1047	0.9496	-0.3646	H	4.2052	1.0938	0.4664
H	2.0661	-1.889	-1.7378	H	2.475	-1.2849	-1.8642
H	2.4761	-2.9835	-0.4291	H	2.8095	-2.6939	-0.8729
H	-0.4209	0.4237	1.728	H	-0.5095	-0.06	1.7835
H	-0.4417	1.7395	0.5947	H	-0.4905	1.4905	0.9832
H	-1.4694	0.2937	-1.1397	H	-1.231	0.5052	-1.1361
H	-2.3487	-1.9459	-0.5431	H	-1.5146	-2.1182	0.4198
H	-1.6712	-1.8155	1.0727	H	-2.023	-1.8418	-1.2391
H	-0.1967	-3.135	-0.3662	H	0.1754	-2.9443	-1.1519
H	-0.1925	-1.8704	-1.5841	H	0.2181	-1.3935	-1.9738
H	-2.8226	1.7488	0.2923	H	-2.8276	1.4748	0.4917

H	-4.318	-0.8328	-0.3992	H	-3.5241	0.6769	-1.7988
H	-5.0288	0.7703	-0.1468	H	-4.0901	-0.8411	-1.061
H	-4.6646	0.3036	-2.3324	H	-5.7239	0.7151	-1.26
H	0.3117	-1.6682	2.2156	H	0.3204	-2.2117	1.7791
H	1.3296	-2.9877	1.652	H	1.4512	-3.3084	0.9926
H	2.0522	-1.4394	2.109	H	2.0498	-1.9313	1.9268
H	-4.1764	0.9009	2.166	H	-4.3106	0.1066	1.8572
H	-2.4658	0.7355	2.5416	H	-2.6491	-0.1622	2.3634
H	-3.4119	-0.6863	2.0609	H	-3.4835	-1.376	1.3759
H	2.3766	2.739	-1.3845	H	1.055	2.7229	1.1833
H	0.8717	2.811	-0.5003	H	2.7988	2.7673	1.2455
H	2.2568	2.5288	1.4326	H	1.3223	2.8153	-1.1695
**5-7**	X axis(Å)	Y axis(Å)	Z axis(Å)	**5-8**	X axis(Å)	Y axis(Å)	Z axis(Å)
C	3.6923	-0.8095	0.1324	C	-3.6926	-0.8724	-0.2518
C	3.3777	0.6034	-0.341	C	-3.3937	0.6185	-0.1767
C	1.9191	0.9996	-0.2135	C	-1.9307	0.9789	-0.3622
C	0.9372	0.095	0.0304	C	-0.9377	0.0561	-0.2791
C	1.217	-1.4166	0.1231	C	-1.2207	-1.4266	0.0331
C	2.6407	-1.7795	-0.3795	C	-2.6668	-1.6529	0.5507
C	-0.5235	0.4681	0.2438	C	0.5377	0.3794	-0.4811
C	-1.453	-0.2904	-0.7197	C	1.4132	-0.1144	0.6859
C	-1.2514	-1.8066	-0.5518	C	1.2076	-1.6224	0.8972
C	0.2119	-2.1953	-0.7681	C	-0.2661	-1.9397	1.1439
C	-2.9521	0.1021	-0.5917	C	2.9209	0.2381	0.5383
C	-3.5231	-0.0578	0.8252	C	3.5606	-0.2762	-0.76
O	-4.9296	0.1588	0.8012	O	4.9665	-0.0576	-0.7192
C	1.0828	-1.8759	1.5941	C	-1.0212	-2.2595	-1.2553
C	-3.1961	1.53	-1.1049	C	3.1543	1.7502	0.6828
C	1.7119	2.4839	-0.3981	C	-1.7417	2.4495	-0.6535
O	2.0593	3.1511	0.8053	O	-1.9525	3.1832	0.5435
H	4.6832	-1.1054	-0.2312	H	-4.6986	-1.0644	0.1391
H	3.7413	-0.8371	1.2269	H	-3.6969	-1.2029	-1.2967
H	3.6676	0.7027	-1.395	H	-3.7215	1.0039	0.7973
H	4.006	1.2993	0.2283	H	-4.0035	1.1201	-0.9381
H	2.6712	-1.7672	-1.4777	H	-2.7474	-1.3409	1.6012
H	2.9048	-2.8014	-0.0796	H	-2.9187	-2.7207	0.5304
H	-0.785	0.238	1.2833	H	0.8607	-0.0797	-1.4225
H	-0.6905	1.5408	0.1409	H	0.7039	1.4501	-0.5991
H	-1.1445	-0.0406	-1.7459	H	1.0579	0.3852	1.5997
H	-1.5737	-2.1392	0.44	H	1.5673	-2.1951	0.0366
H	-1.8714	-2.3455	-1.2786	H	1.7913	-1.9589	1.7626
H	0.3224	-3.275	-0.604	H	-0.3816	-3.0243	1.2673

H	0.448	-2.0216	-1.8272	H	-0.5465	-1.4913	2.1072
H	-3.5274	-0.5557	-1.2584	H	3.4555	-0.2263	1.3789
H	-3.3529	-1.0654	1.2132	H	3.3991	-1.3497	-0.8871
H	-3.0818	0.6655	1.5181	H	3.1632	0.2459	-1.636
H	-5.2525	0.0484	1.7119	H	5.3336	-0.3956	-1.5539
H	0.0685	-1.7472	1.9843	H	0.0074	-2.2176	-1.627
H	1.3309	-2.9386	1.697	H	-1.2555	-3.3159	-1.0796
H	1.7511	-1.3141	2.2559	H	-1.6658	-1.9084	-2.0686
H	-4.2685	1.7391	-1.1822	H	4.2234	1.9742	0.7624
H	-2.7655	1.6654	-2.1026	H	2.6729	2.1351	1.588
H	-2.7601	2.2813	-0.4392	H	2.7635	2.3057	-0.1754
H	2.3527	2.864	-1.2013	H	-0.7594	2.7107	-1.0482
H	0.6918	2.7598	-0.671	H	-2.4664	2.7913	-1.4003
H	1.6526	2.645	1.529	H	-1.4286	2.7403	1.2329

Functional	Solvent?	Basis Set	Type of Data
mPW1PW91	PCM	6-311+G(d,p)	Unscaled Shifts

	Isomer 1	Isomer 2	Isomer 3	Isomer 4	Isomer 5	Isomer 6
sDP4+ (H data)	0.06%	99.94%	-	-	-	-
sDP4+ (C data)	0.92%	99.08%	-	-	-	-
sDP4+ (all data)	0.00%	100.00%	-	-	-	-
uDP4+ (H data)	0.53%	99.47%	-	-	-	-
uDP4+ (C data)	62.69%	37.31%	-	-	-	-
uDP4+ (all data)	0.88%	99.12%	-	-	-	-
DP4+ (H data)	0.00%	100.00%	-	-	-	-
DP4+ (C data)	1.53%	98.47%	-	-	-	-
DP4+ (all data)	0.00%	100.00%	-	-	-	-

Figure S6: DP4+ results of candidate(7*R*,10*R*)-**1** (Isomer 1) and (7*R*,11*S*)-**1** (Isomer 2).

Functional	Solvent?	Basis Set	Type of Data
mPW1PW91	PCM	6-311+G(d,p)	Unscaled Shifts

	Isomer 1	Isomer 2	Isomer 3	Isomer 4	Isomer 5	Isomer 6
sDP4+ (H data)	0.00%	100.00%	-	-	-	-
sDP4+ (C data)	2.67%	97.33%	-	-	-	-
sDP4+ (all data)	0.00%	100.00%	-	-	-	-
uDP4+ (H data)	0.00%	100.00%	-	-	-	-
uDP4+ (C data)	0.02%	99.98%	-	-	-	-
uDP4+ (all data)	0.00%	100.00%	-	-	-	-
DP4+ (H data)	0.00%	100.00%	-	-	-	-
DP4+ (C data)	0.00%	100.00%	-	-	-	-
DP4+ (all data)	0.00%	100.00%	-	-	-	-

Figure S7: DP4+ results of candidate(7*R*,10*R*,11*R*)-**3** (Isomer 1) and (7*R*,10*R*,11*S*)-**3** (Isomer 2).

Functional		Solvent?		Basis Set		Type of Data	
mPW1PW91		PCM		6-311+G(d,p)		Unscaled Shifts	
	Isomer 1	Isomer 2	Isomer 3	Isomer 4	Isomer 5	Isomer 6	
sDP4+ (H data)	0.00%	100.00%	-	-	-	-	
sDP4+ (C data)	62.34%	37.66%	-	-	-	-	
sDP4+ (all data)	0.00%	100.00%	-	-	-	-	
uDP4+ (H data)	0.00%	100.00%	-	-	-	-	
uDP4+ (C data)	0.20%	99.80%	-	-	-	-	
uDP4+ (all data)	0.00%	100.00%	-	-	-	-	
DP4+ (H data)	0.00%	100.00%	-	-	-	-	
DP4+ (C data)	0.33%	99.67%	-	-	-	-	
DP4+ (all data)	0.00%	100.00%	-	-	-	-	

Figure S8: DP4$^+$ results of candidate(7*S*,10*S*,11*S*)-**5** (Isomer 1) and (7*R*,10*R*,11*S*)-**5** (Isomer 2).

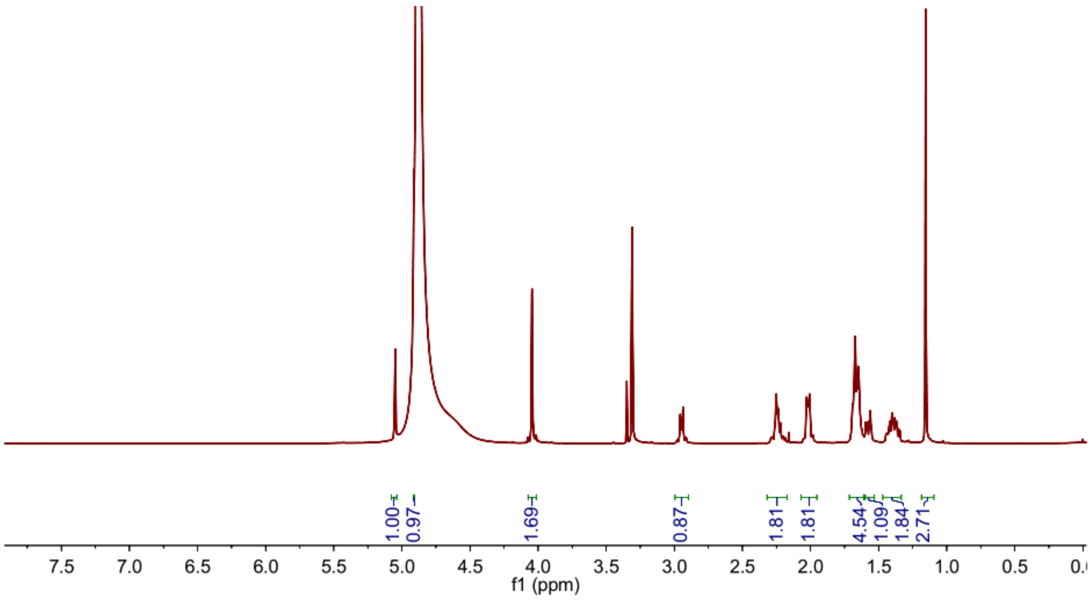

Figure S9: ^{1}H NMR (500 MHz) spectrum of **1** in CD_3OD.

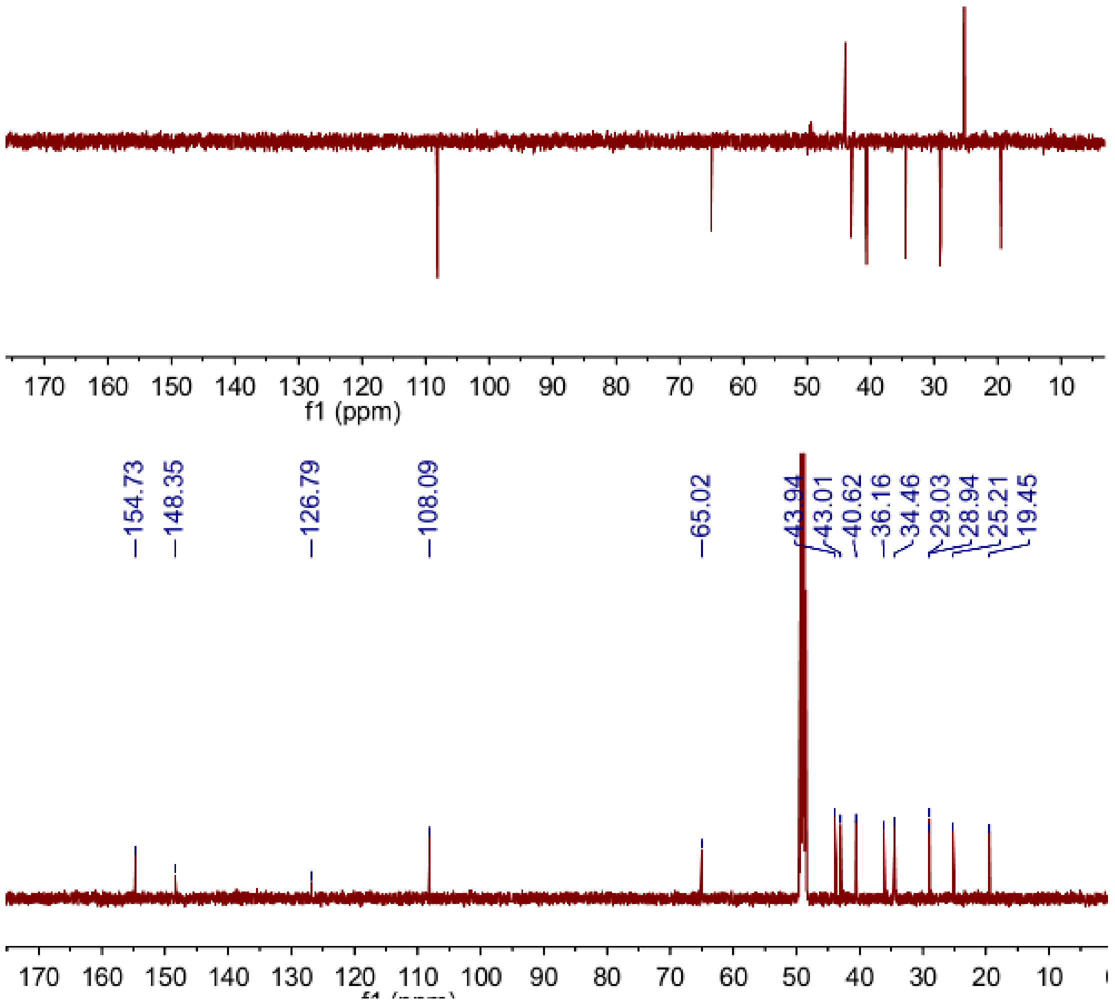

Figure S10: ^{13}C NMR and DEPT (125 MHz) spectra of **1** in CD_3OD.

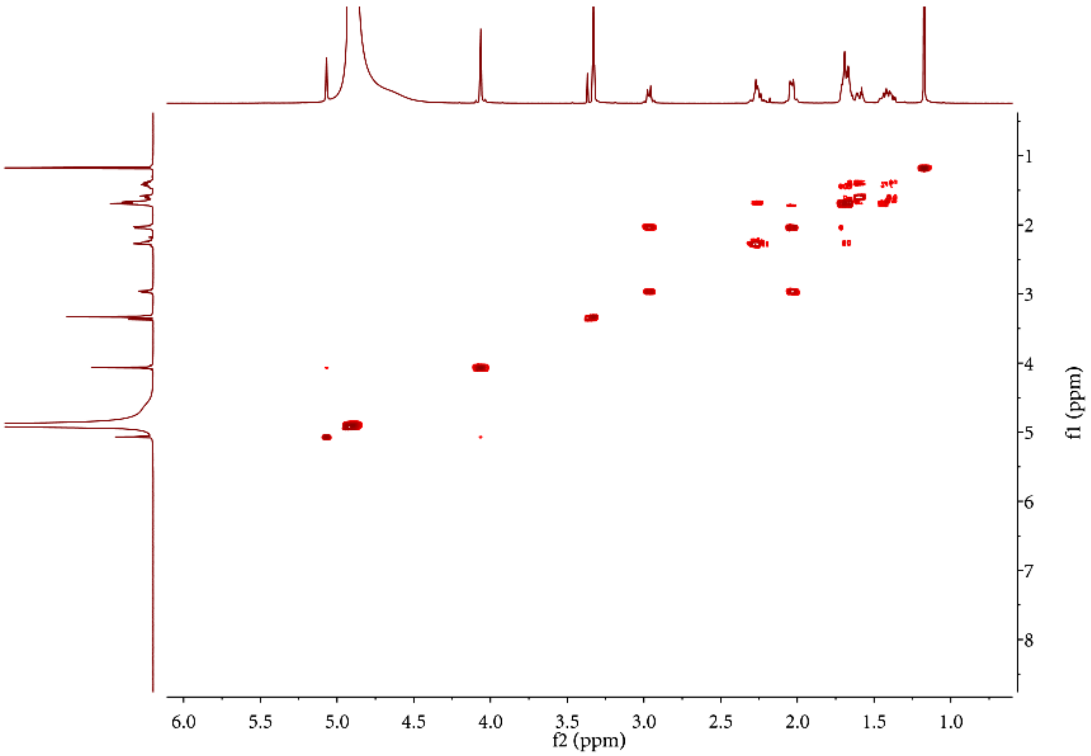

Figure S11: ^{1}H-^{1}H COSY (500 MHz) spectrum of **1** in CD_3OD.

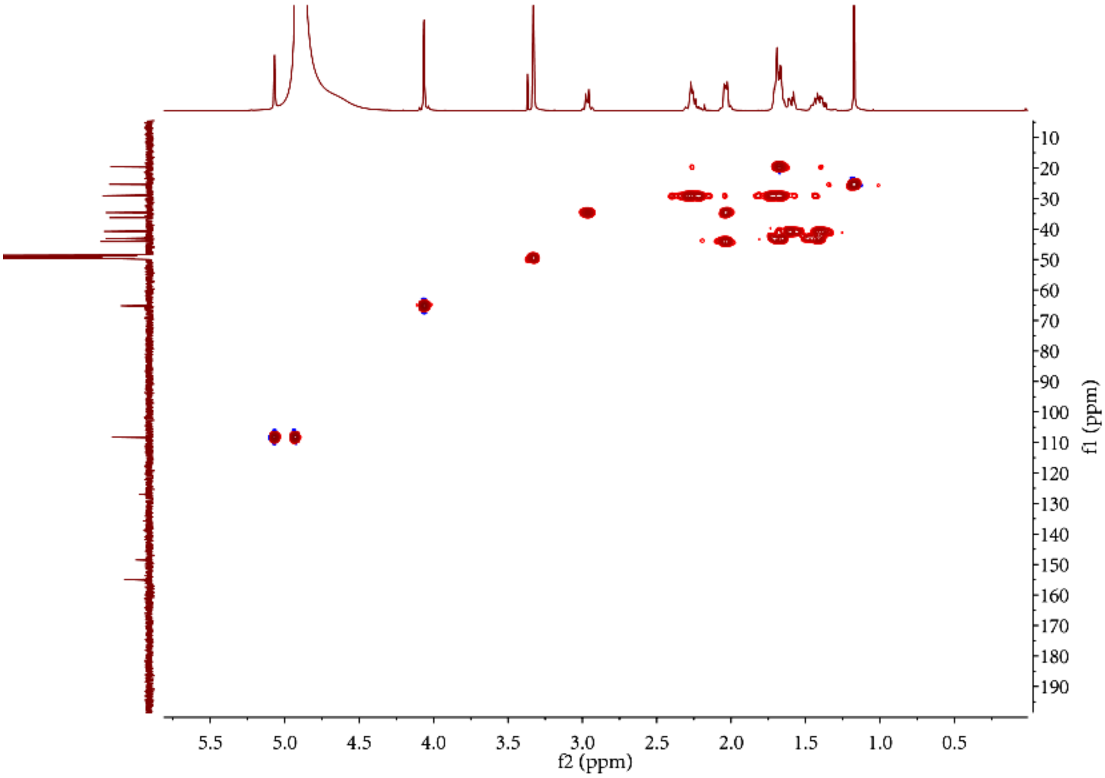

Figure S12: HSQC (500 MHz) spectrum of **1** in CD_3OD.

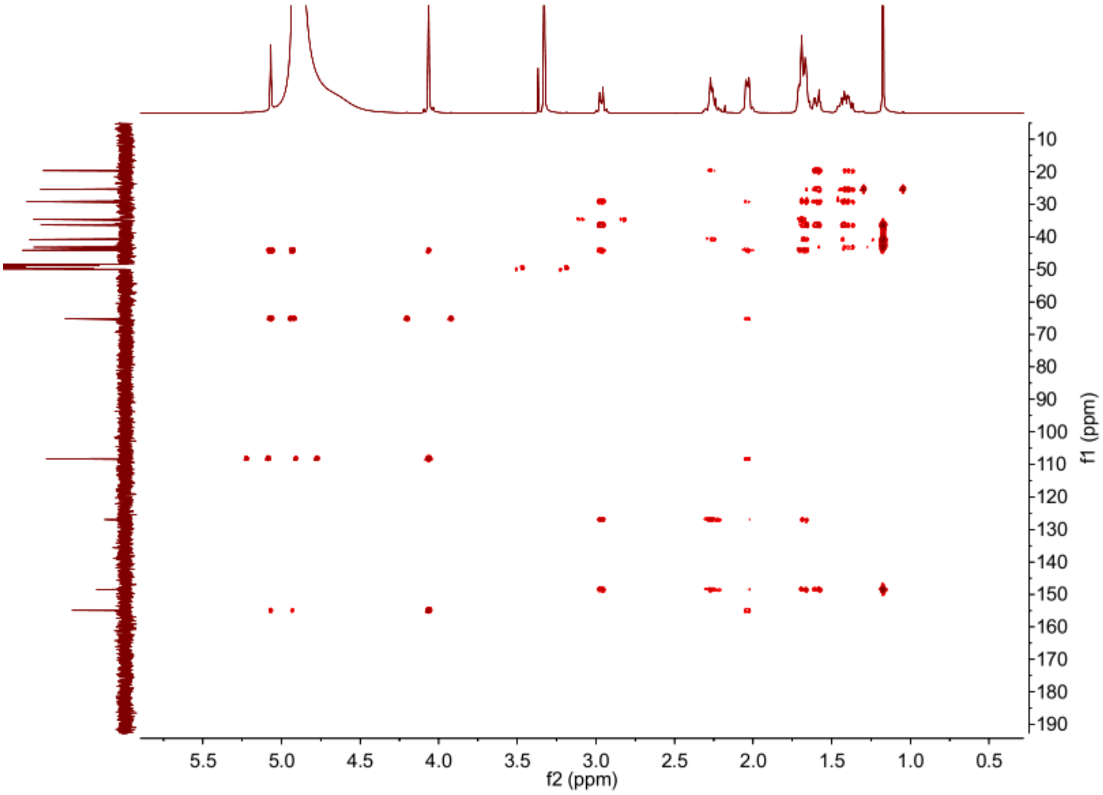

Figure S13: HSBC (500 MHz) spectrum of **1** in CD_3OD.

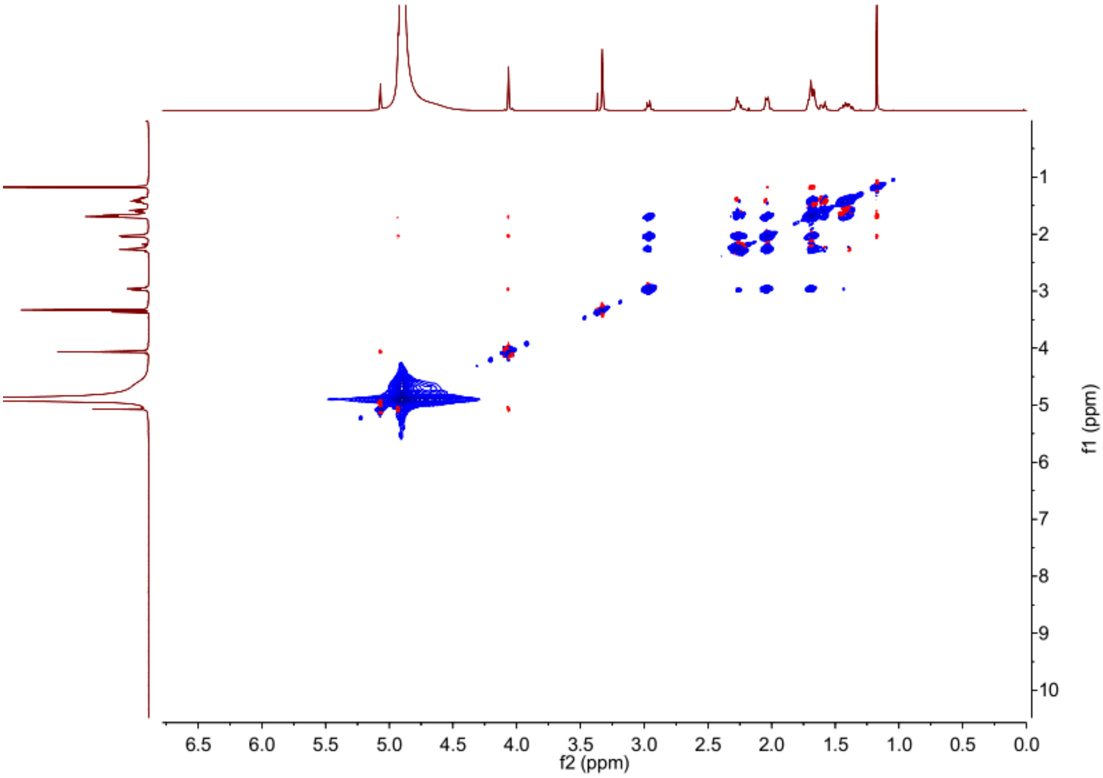

Figure S14: ROSEY (500 MHz) spectrum of **1** in CD_3OD.

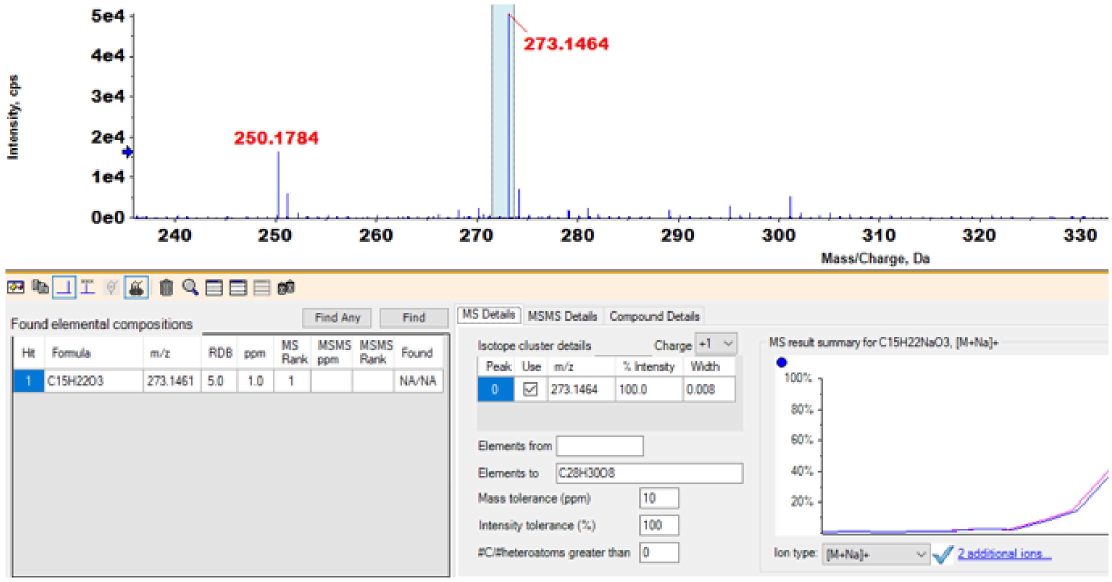

Figure S15: HRESIMS of **1**.

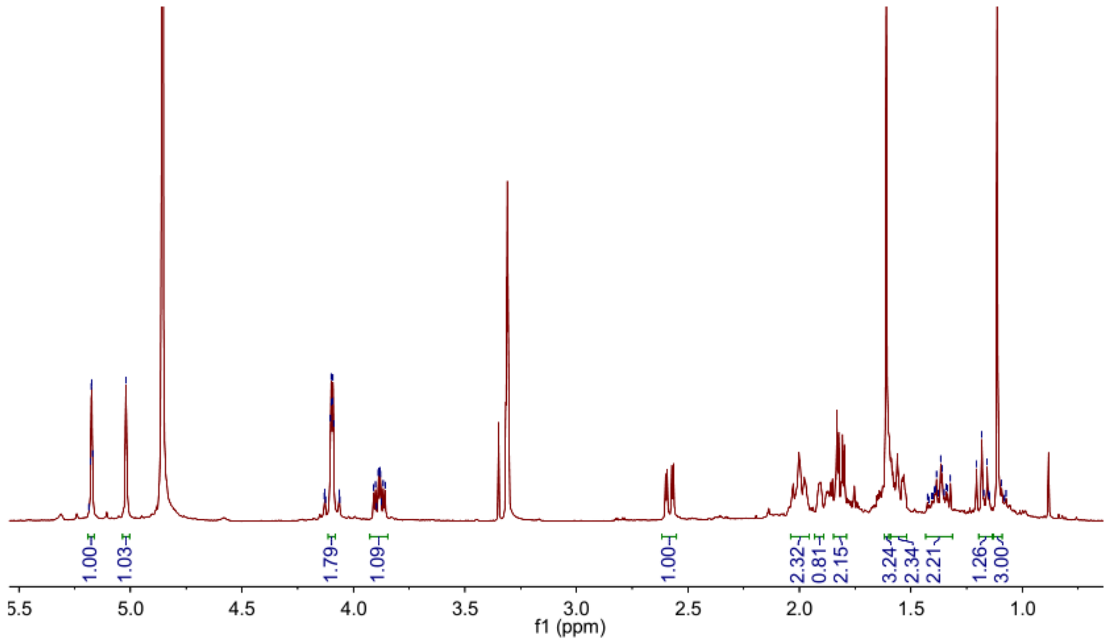

Figure S16: ^{1}H NMR (500 MHz) spectrum of **2** in CD_3OD.

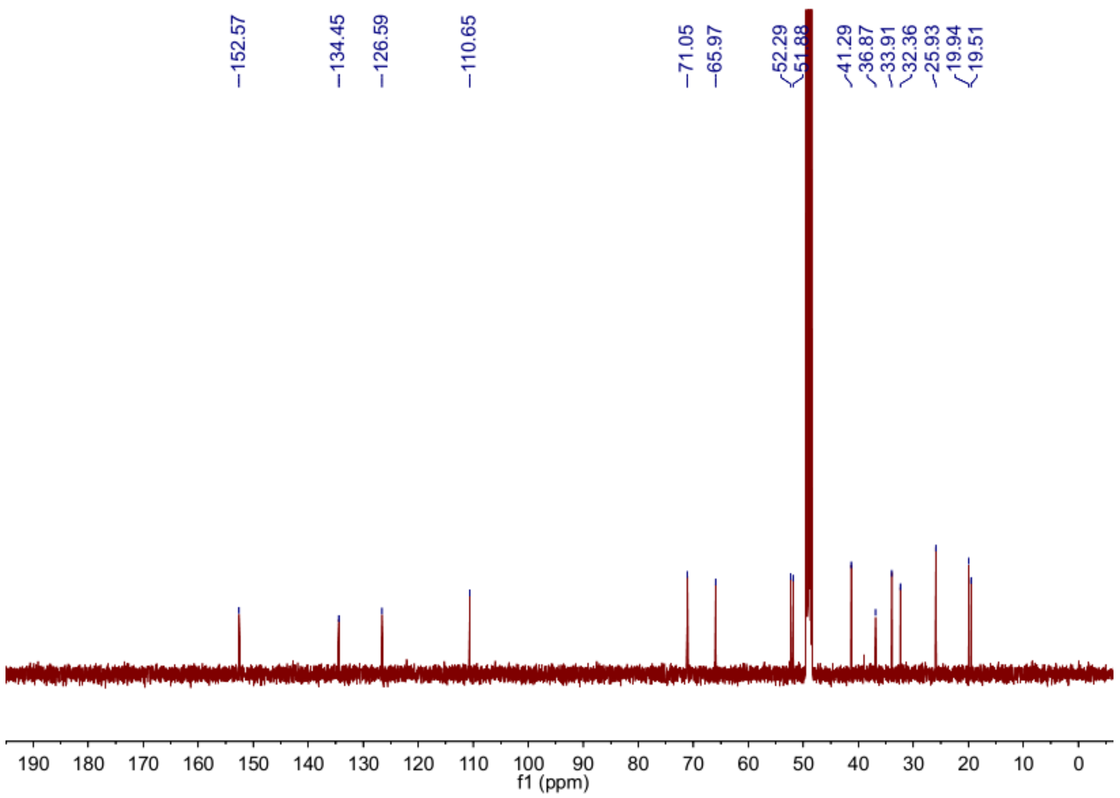

Figure S17: ^{13}C NMR (125MHz) spectrum of **2** in CD_3OD.

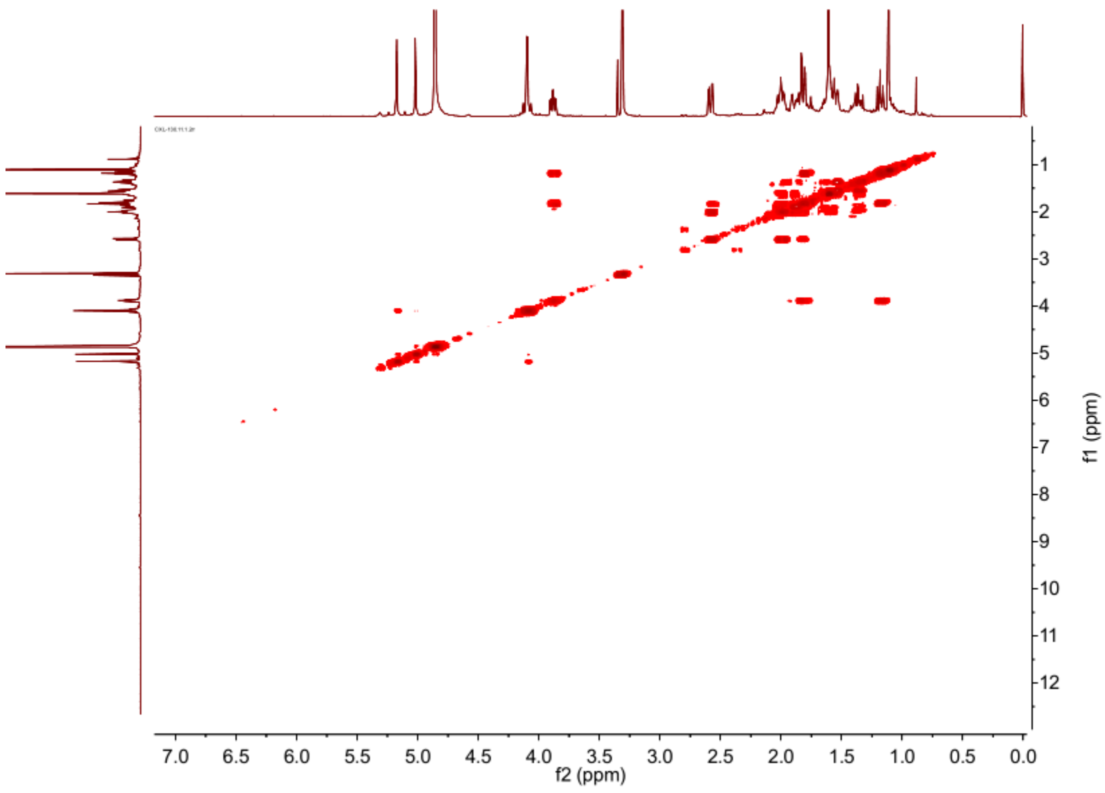

Figure S18: ^{1}H-^{1}H COSY (500 MHz) spectrum of **2** in CD_3OD.

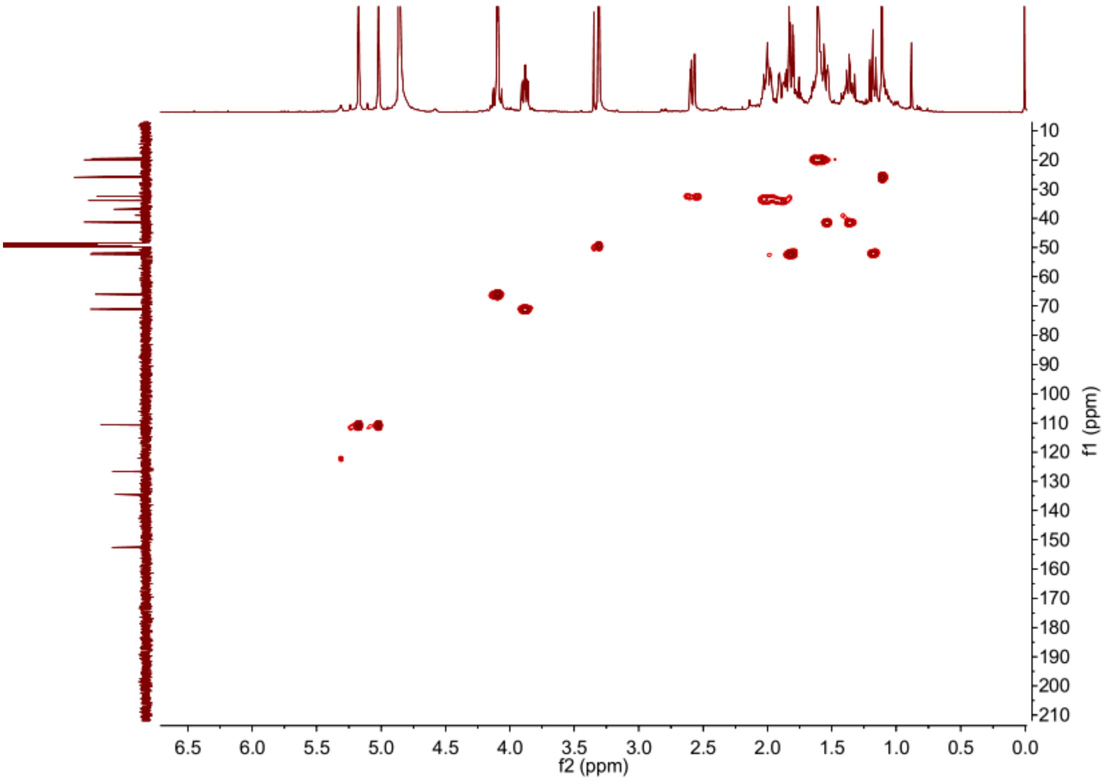

Figure S19: HSQC (500 MHz) spectrum of **2** in CD_3OD.

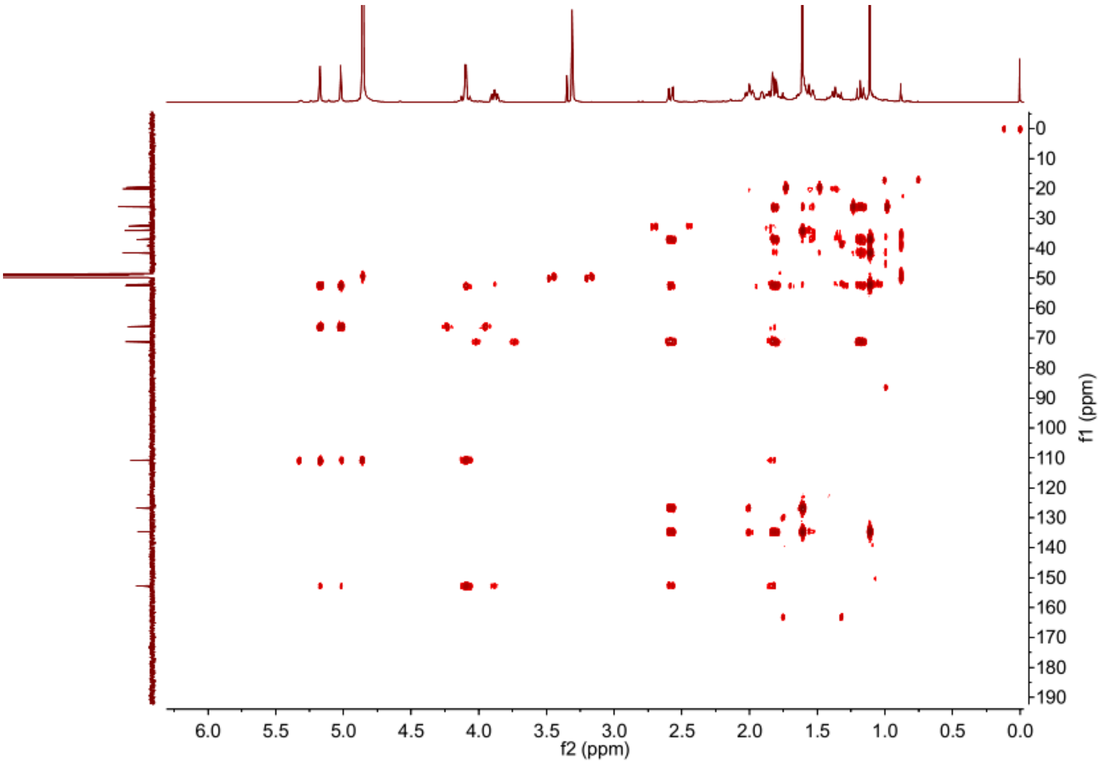

Figure S20: HSBC (500 MHz) spectrum of **2** in CD_3OD.

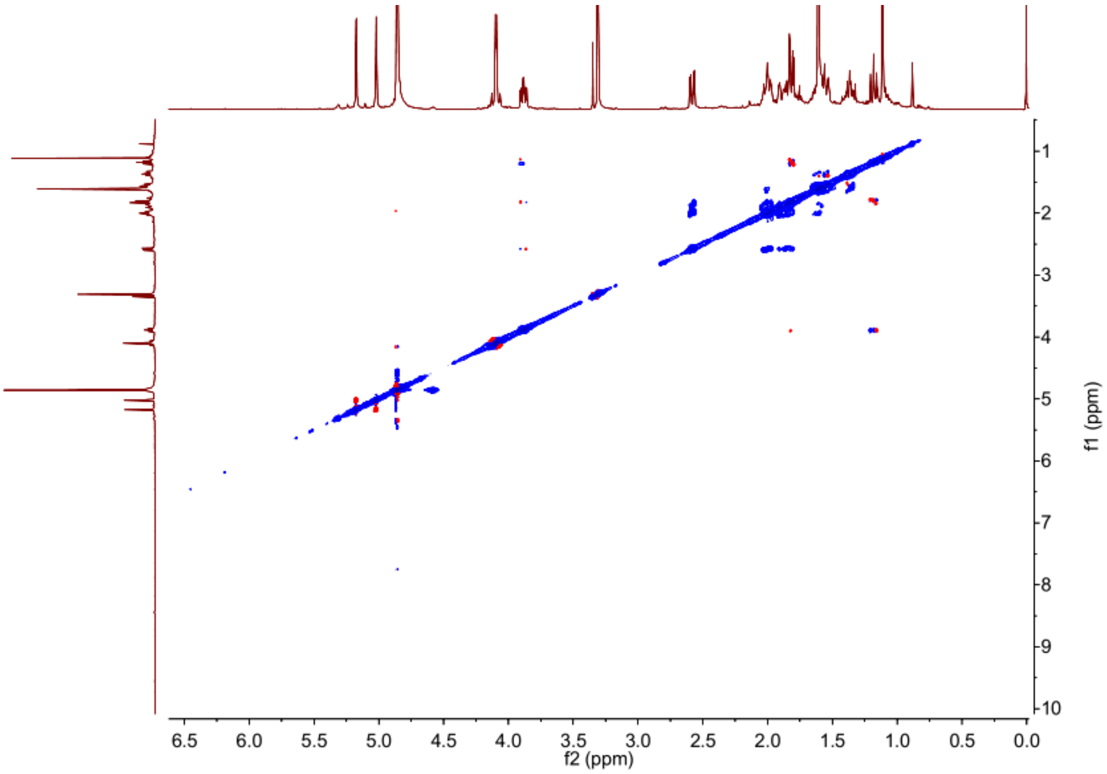

Figure S21: ROSEY (500 MHz) spectrum of **2** in CD_3OD.

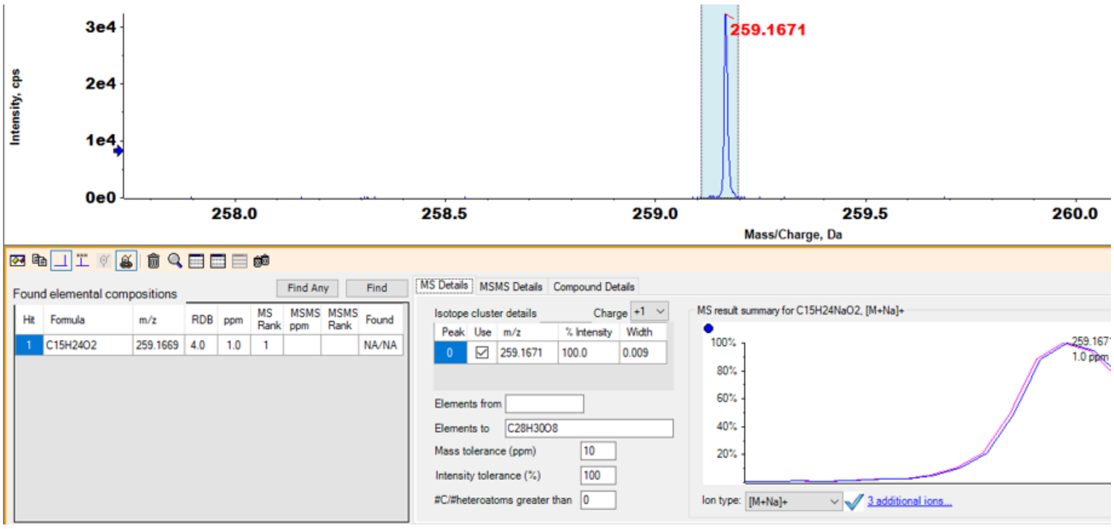

Figure S22: HRESIMS of **2**.

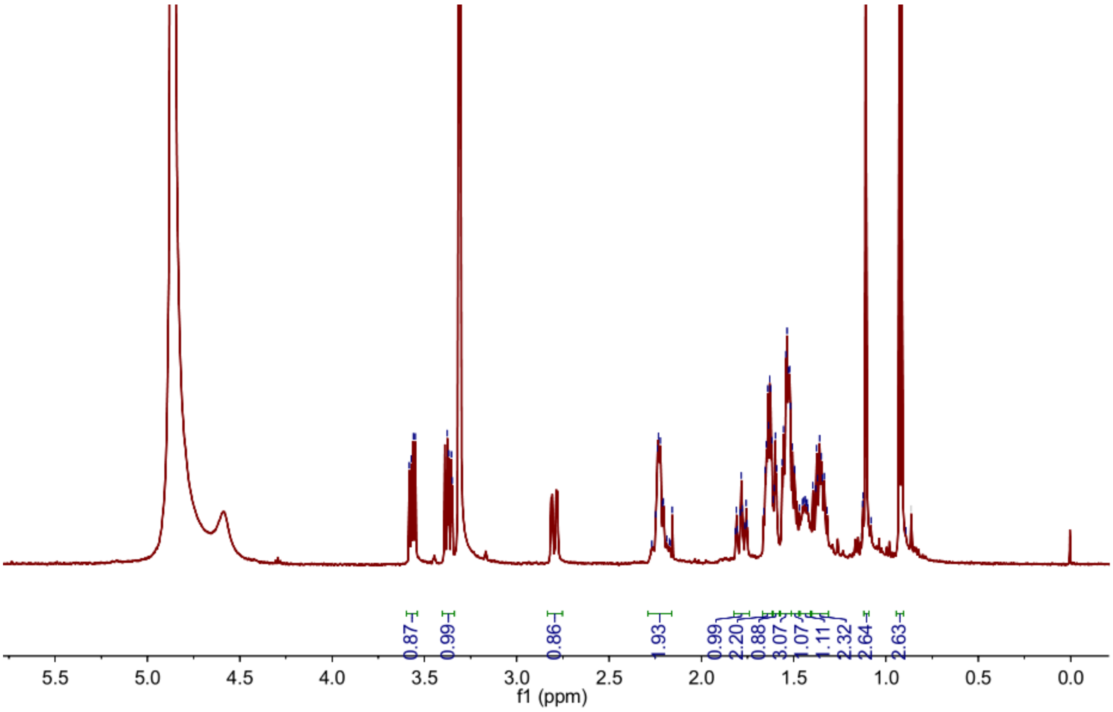

Figure S23: ^{1}H NMR (500 MHz) spectrum of **3** in CD_3OD.

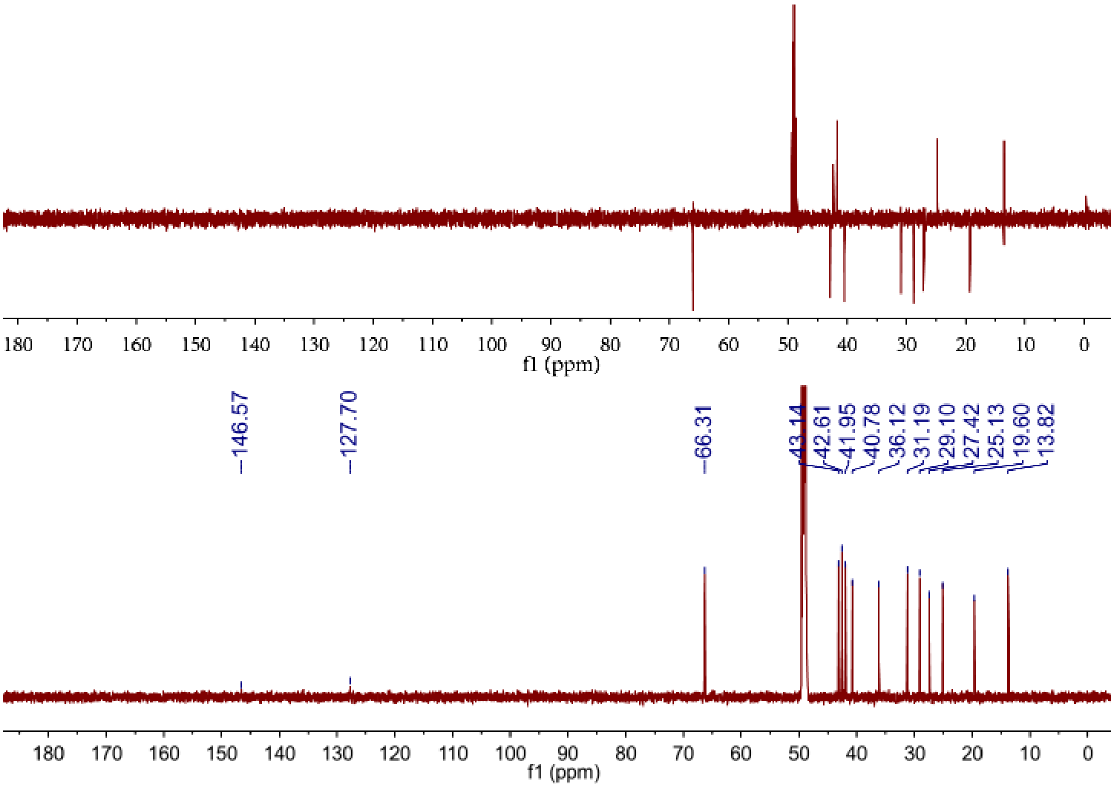

Figure S24: ^{13}C NMR and DEPT (150 MHz) spectra of **3** in CD_3OD.

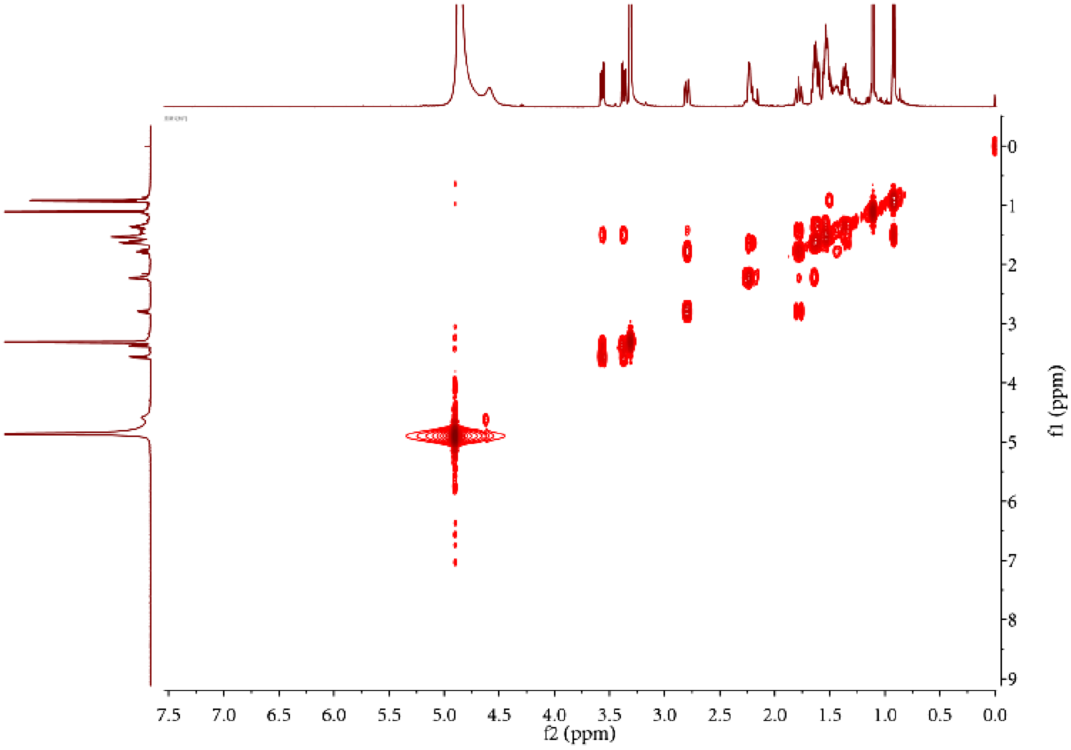

Figure S25: ^1H-^1H COSY (600 MHz) spectrum of **3** in CD_3OD.

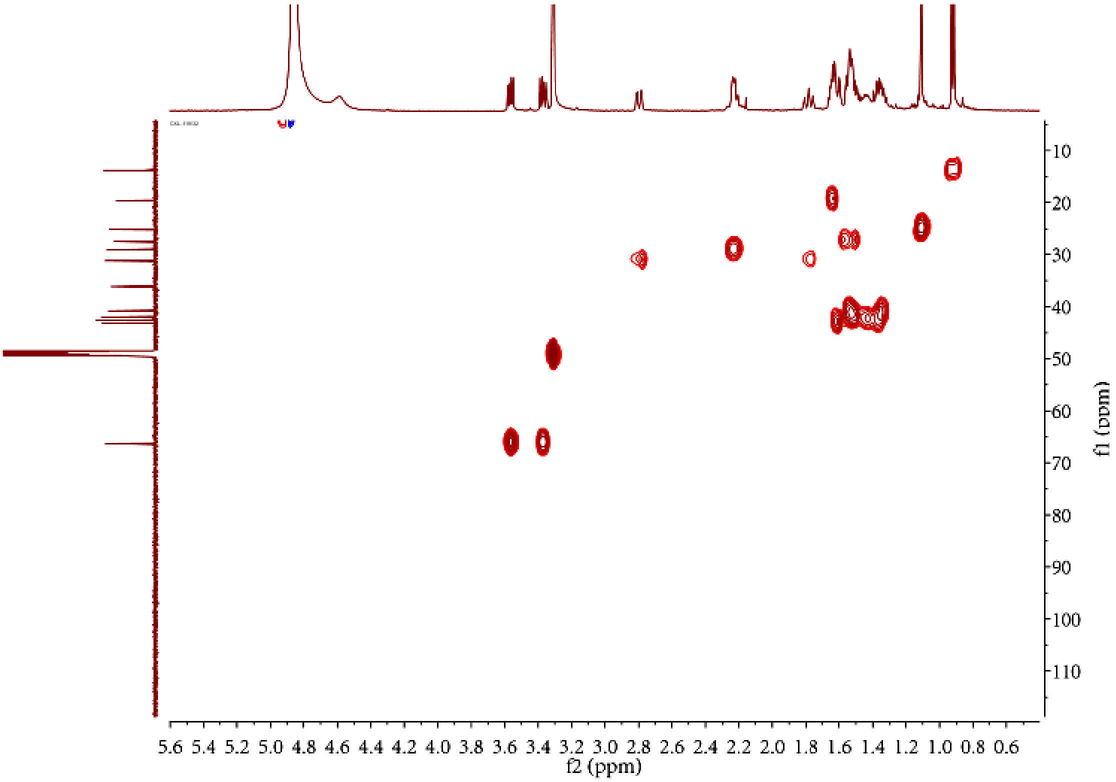

Figure S26: HSQC (600 MHz) spectrum of **3** in CD_3OD.

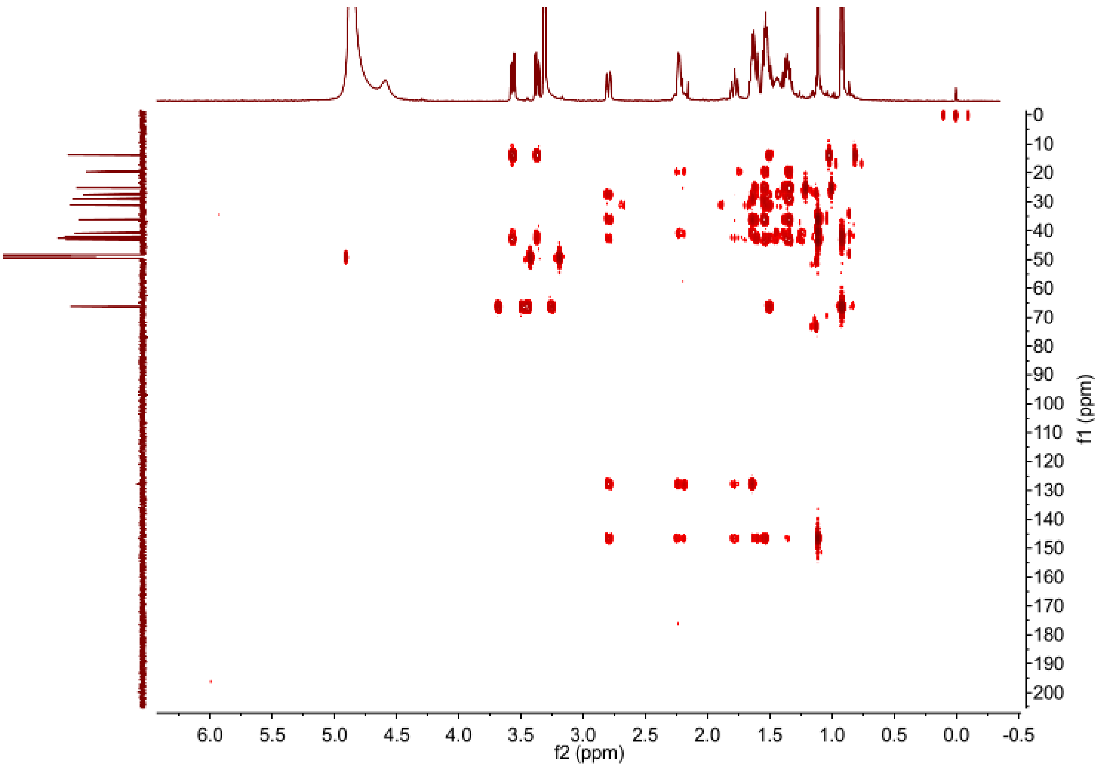

Figure S27: HSBC (600 MHz) spectrum of **3** in CD_3OD.

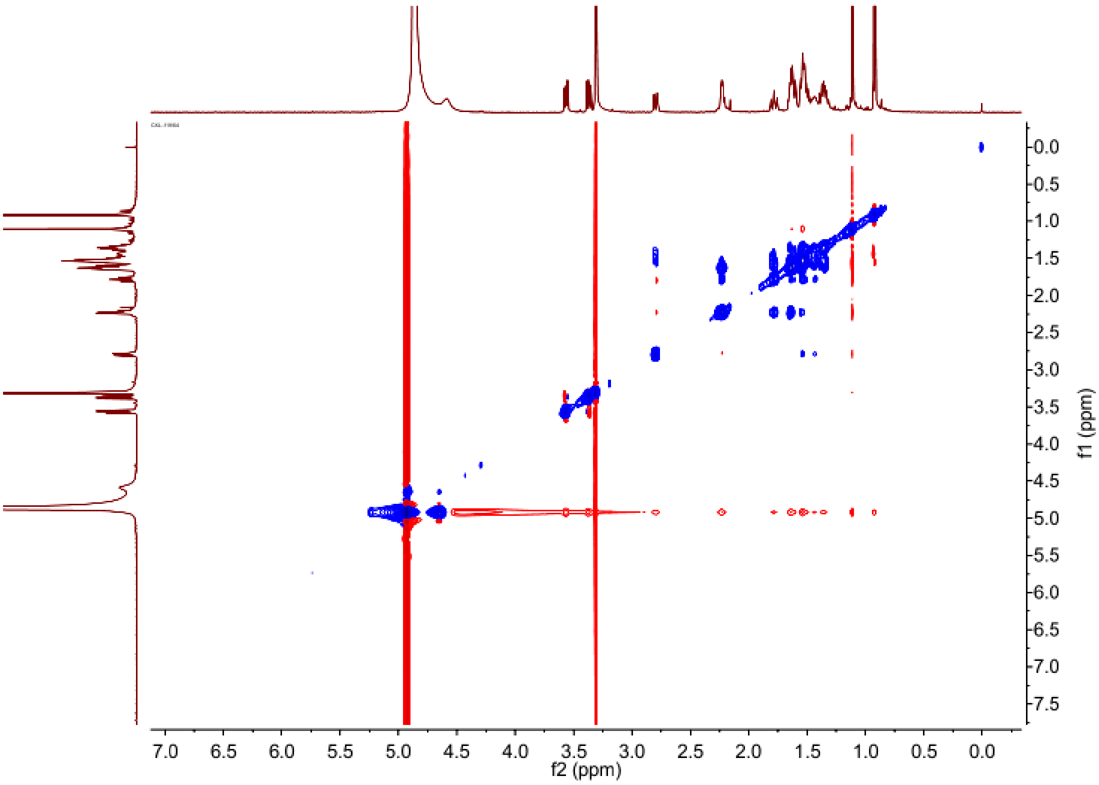

Figure S28: ROSEY (600 MHz) spectrum of **3** in CD_3OD.

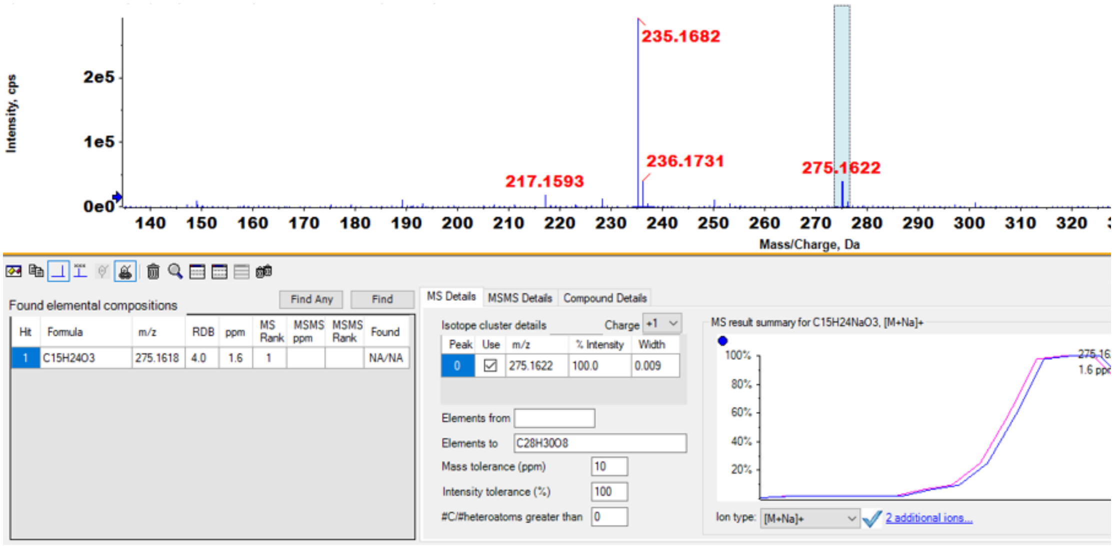

Figure S29: HRESIMS of **3**.

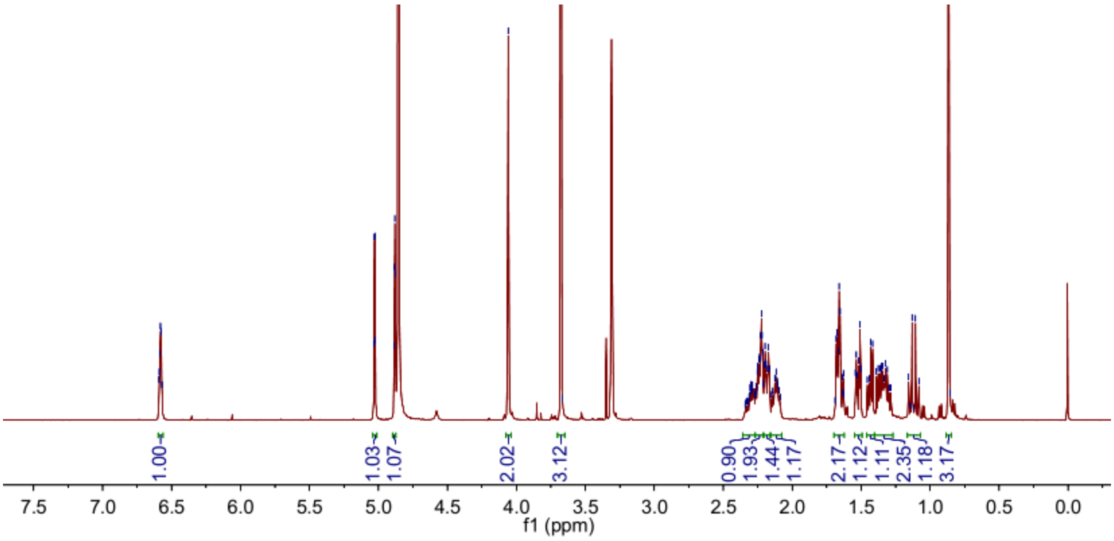

Figure S30: ^{1}H NMR (500 MHz) spectrum of **4** in CD_3OD.

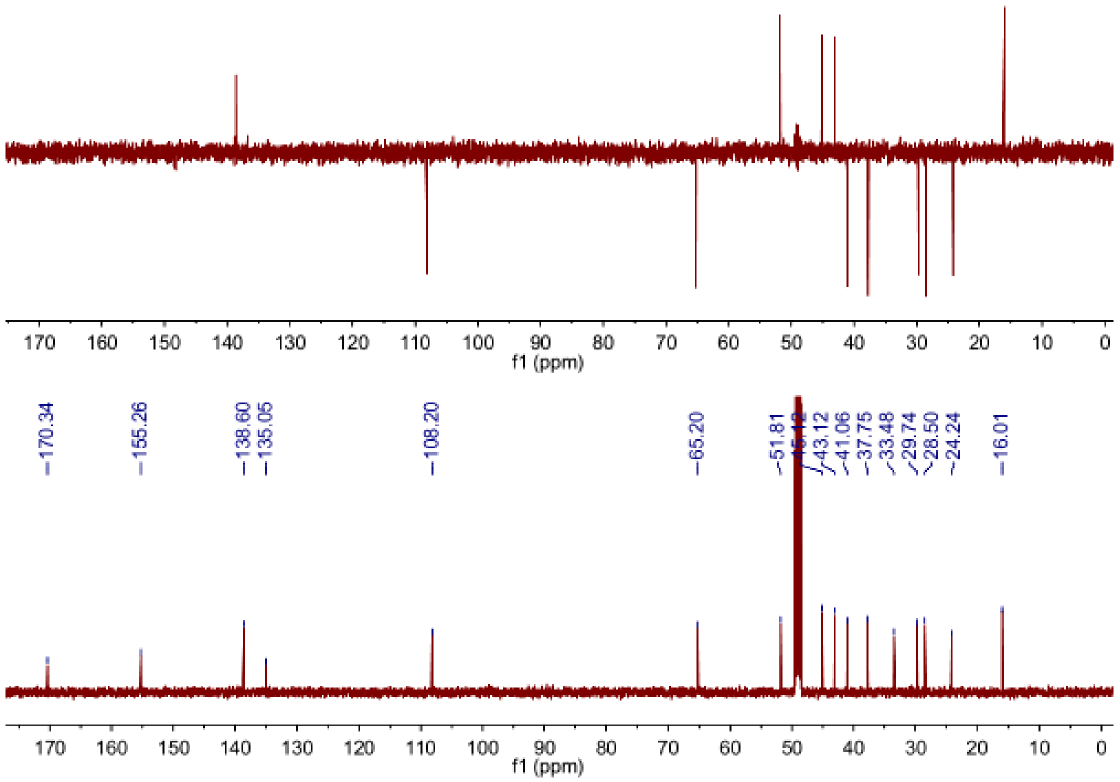

Figure S31: ^{13}C NMR and DEPT (150 MHz) spectra of **4** in CD_3OD.

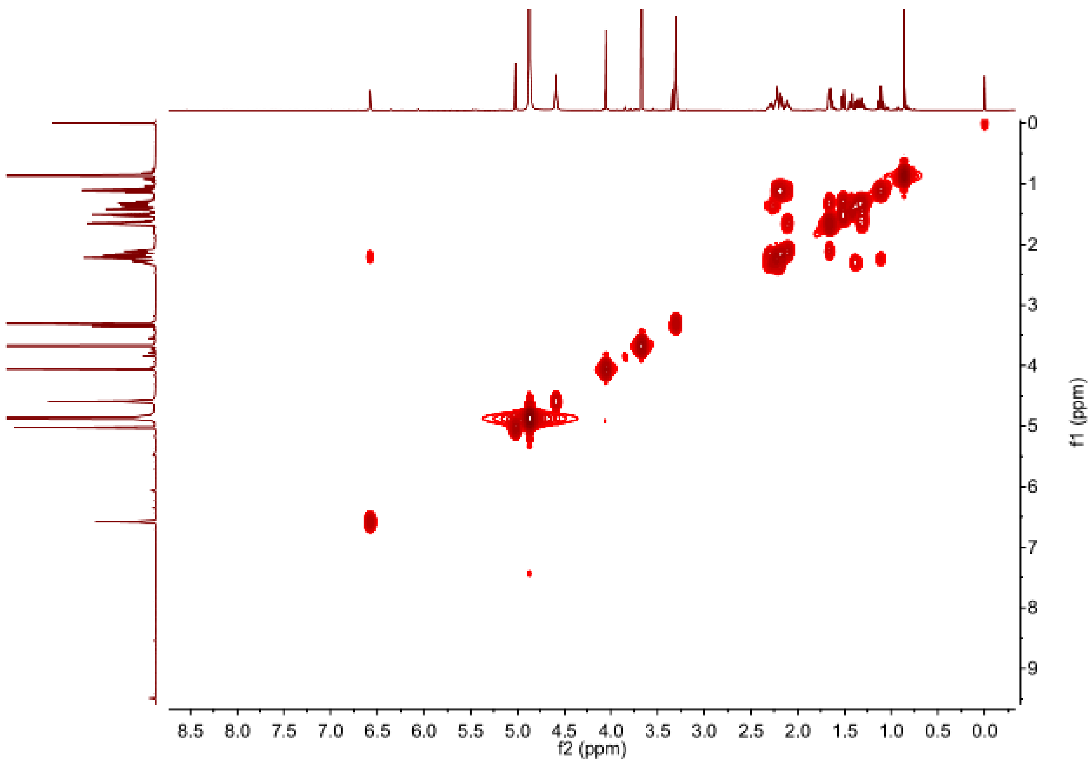

Figure S32: ^{1}H-^{1}H COSY (600 MHz) spectrum of **4** in CD_3OD.

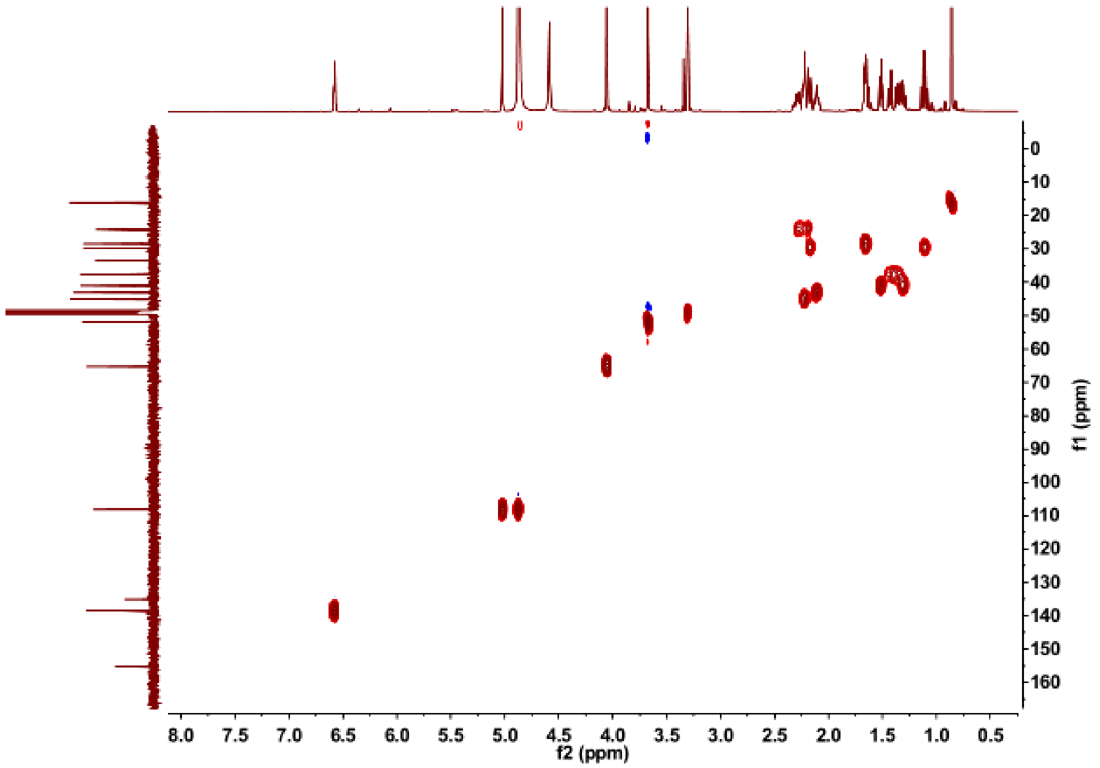

Figure S33: HSQC (600 MHz) spectrum of **4** in CD_3OD.

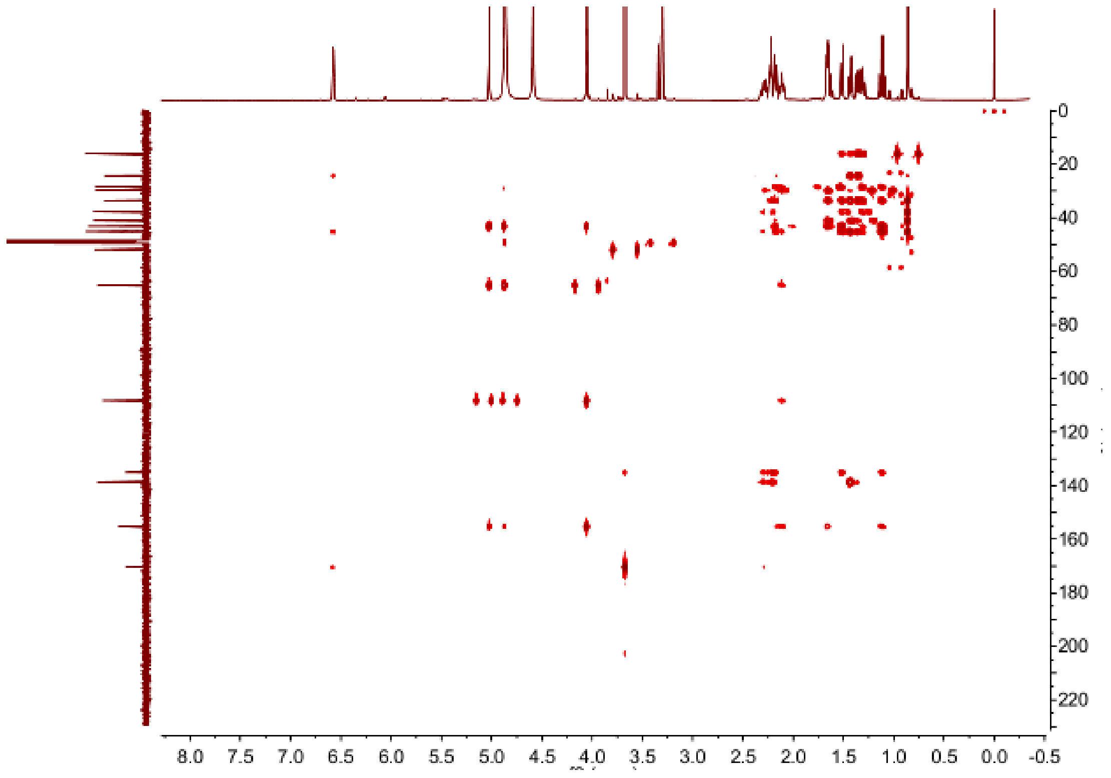

Figure S34: HSBC (600 MHz) spectrum of **4** in CD_3OD.

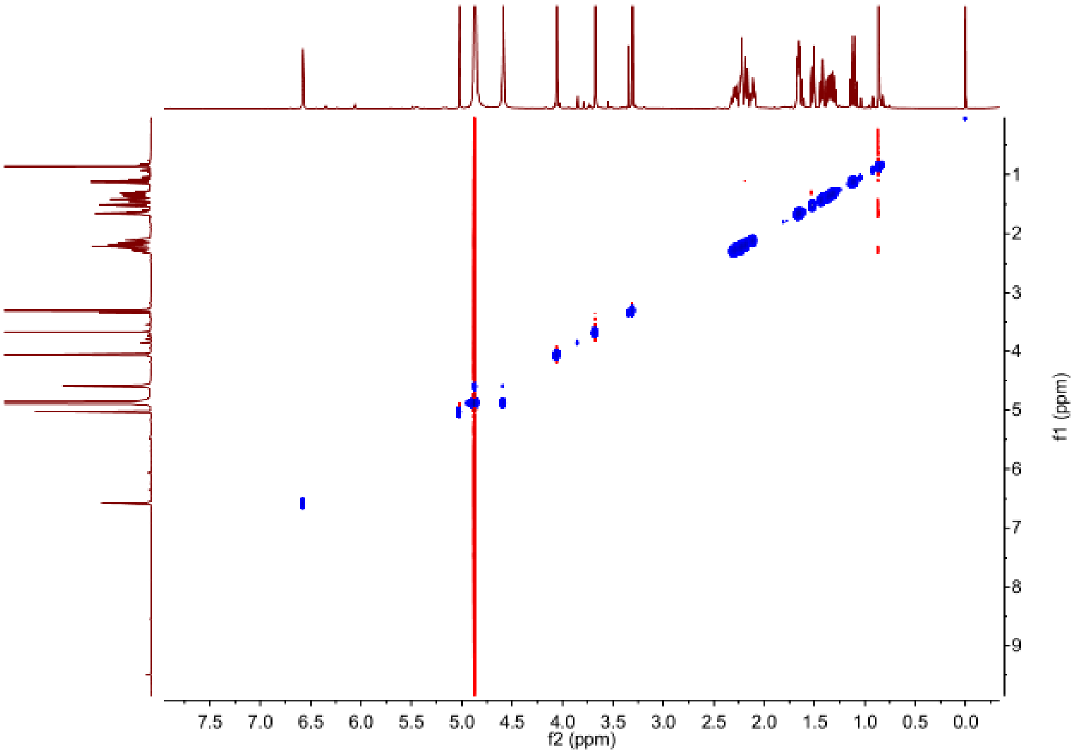

Figure S35: ROSEY (600 MHz) spectrum of **4** in CD_3OD.

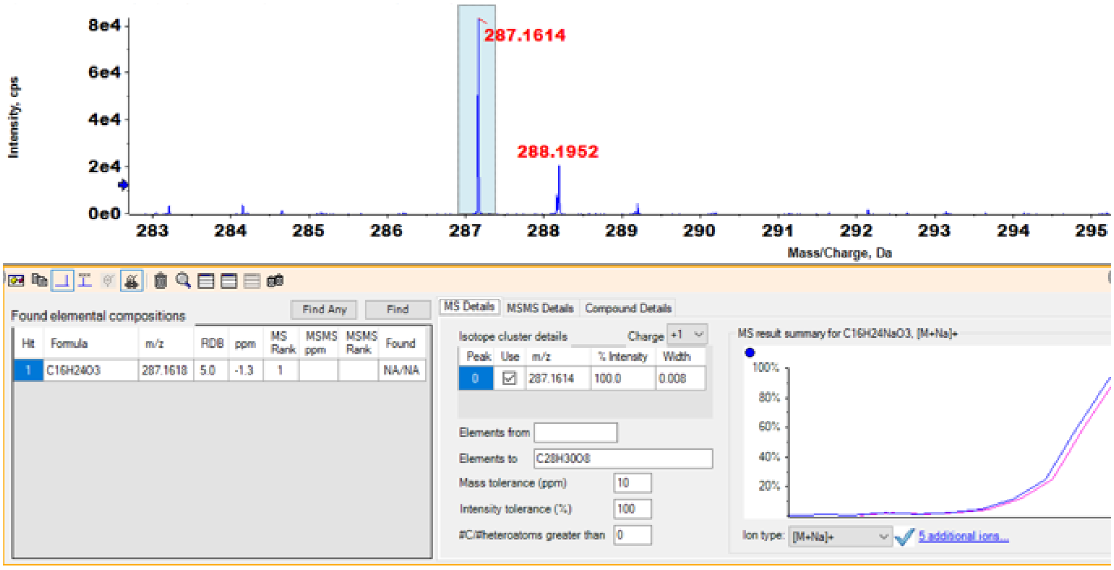

Figure S36: HRESIMS of **4**.

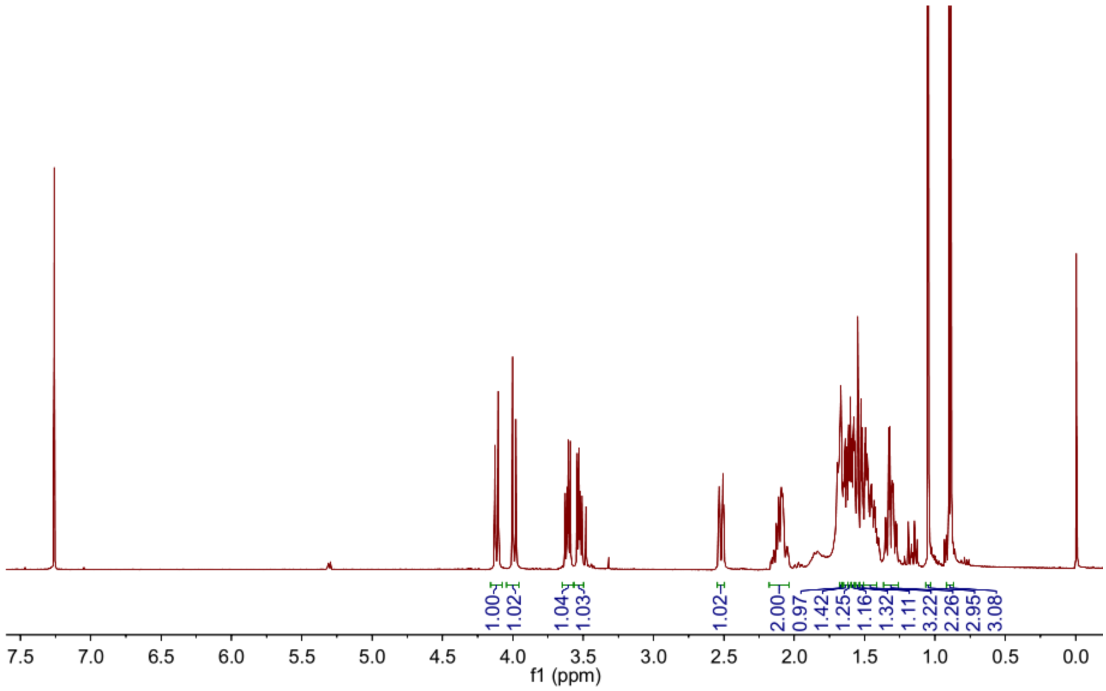

Figure S37: ^{1}H NMR (500 MHz) spectrum of **5** in $CDCl_3$.

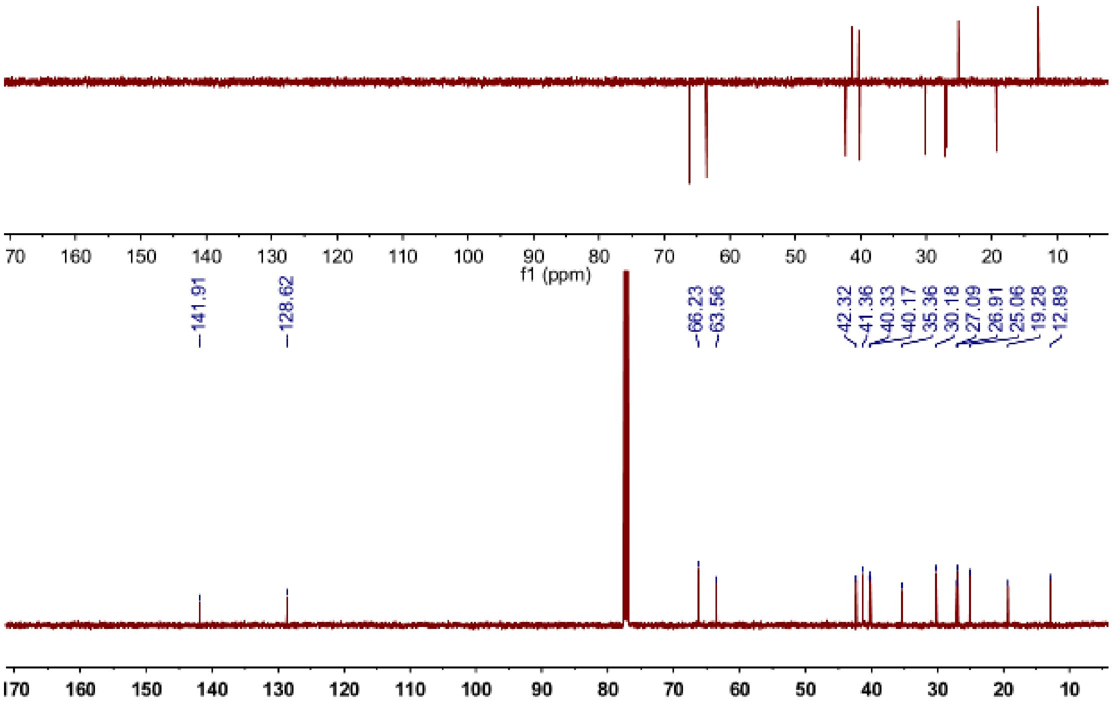

Figure S38: ^{13}C NMR and DEPT (150 MHz) spectra of **5** in $CDCl_3$.

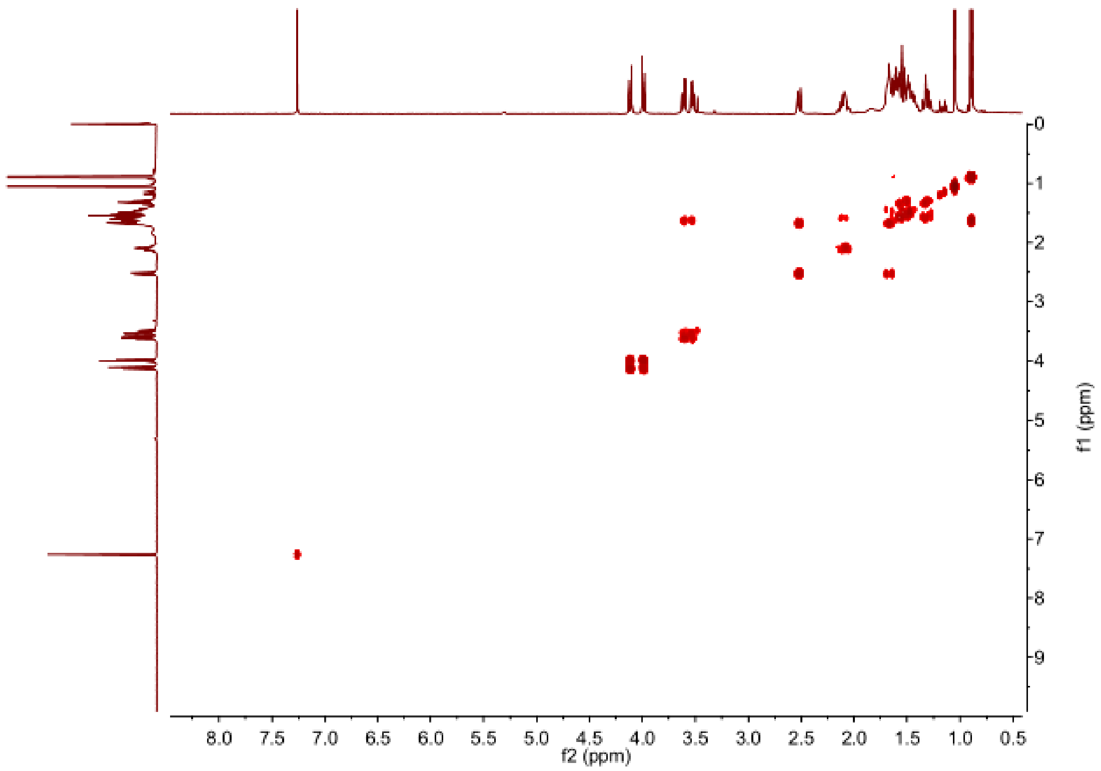

Figure S39: ^1H-^1H COSY (600 MHz) spectrum of **5** in $CDCl_3$.

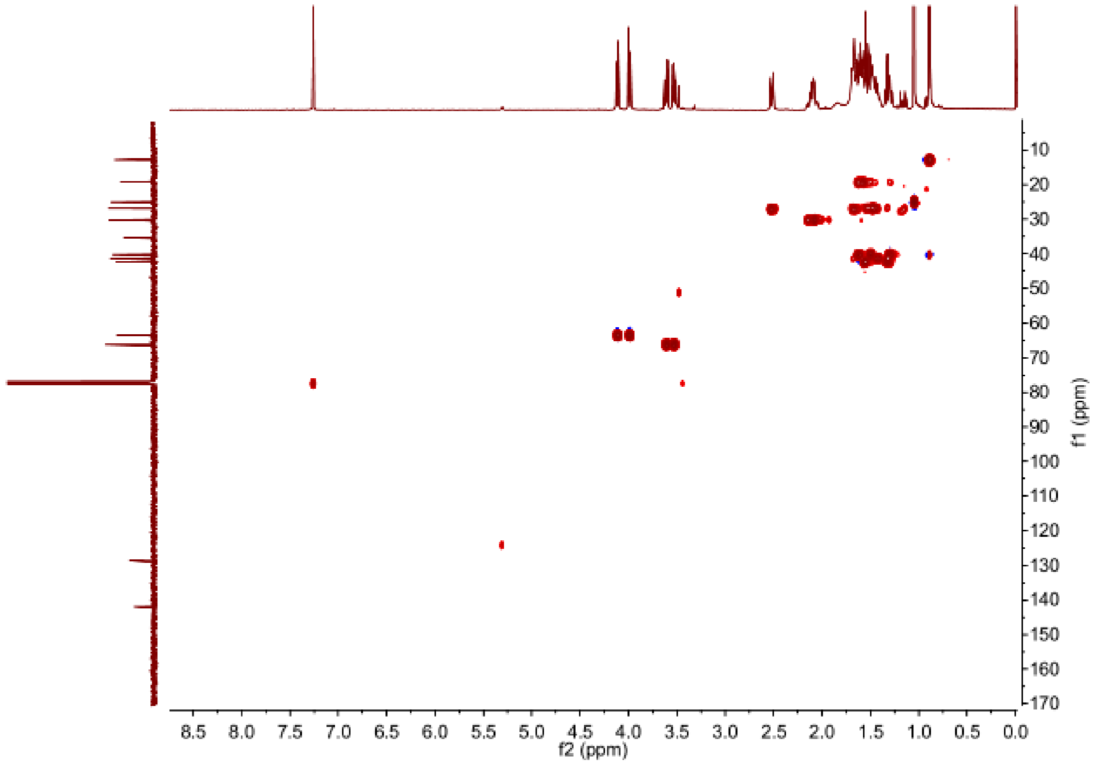

Figure S40: HSQC (600 MHz) spectrum of **5** in $CDCl_3$.

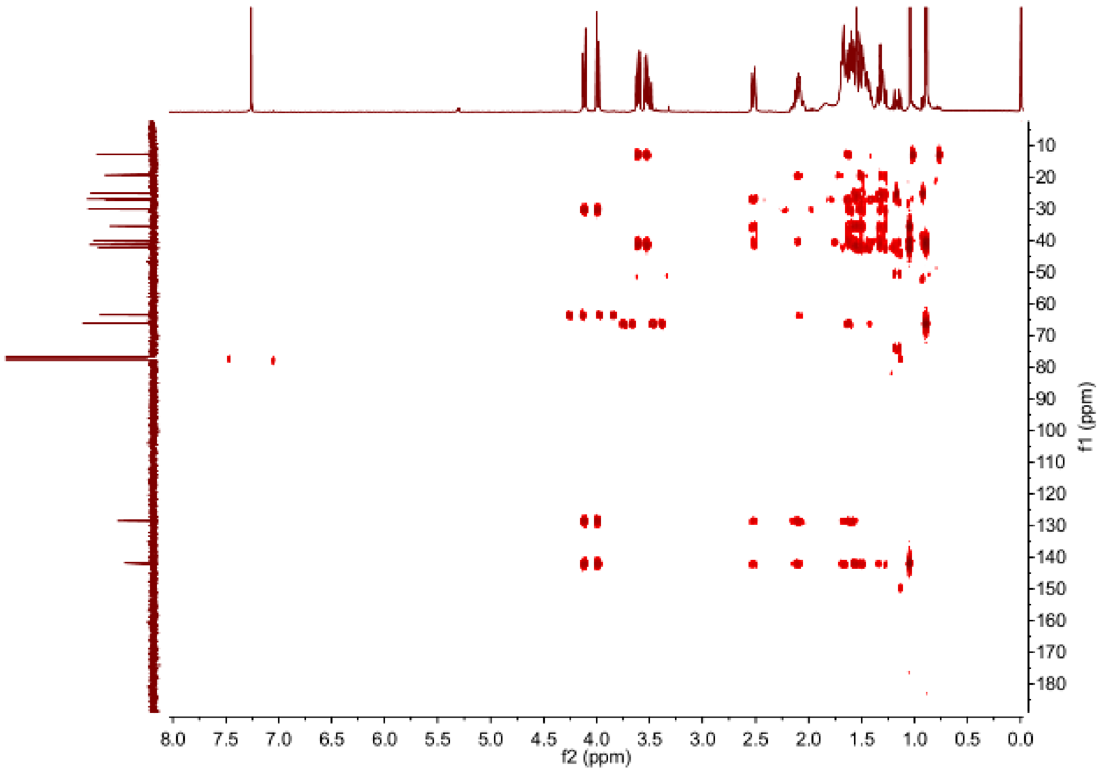

Figure S41: HSBC (600 MHz) spectrum of **5** in $CDCl_3$.

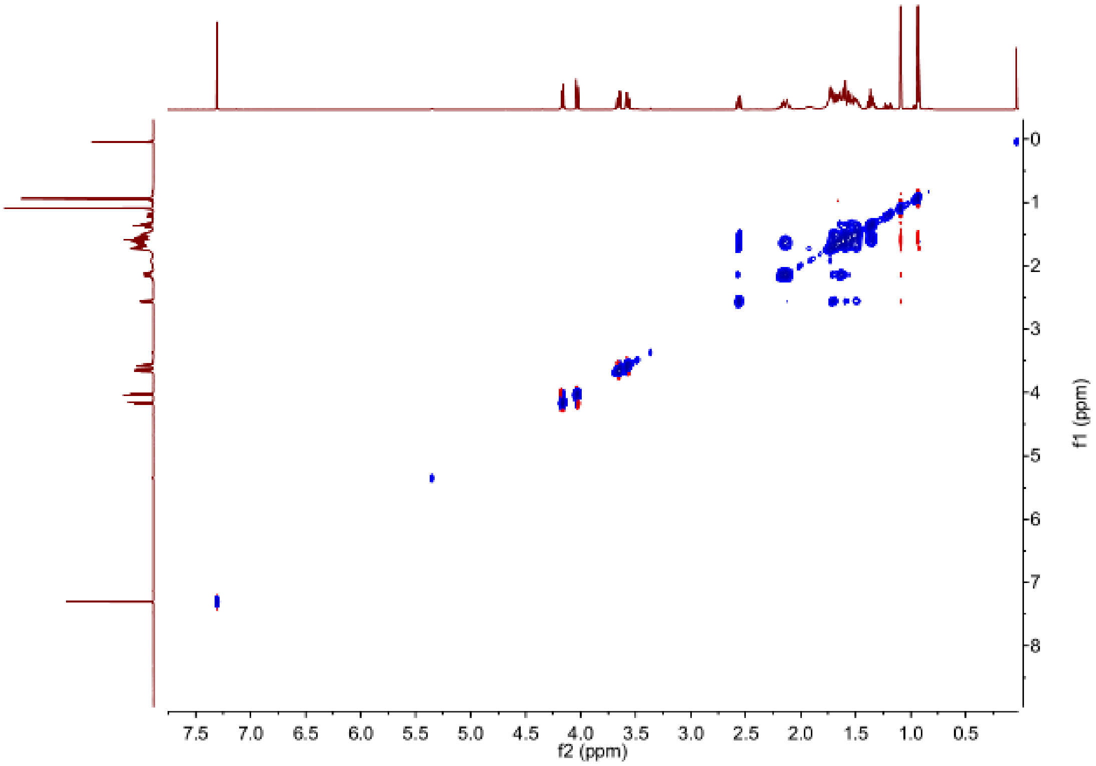

Figure S42: ROSEY (600 MHz) spectrum of **5** in $CDCl_3$.

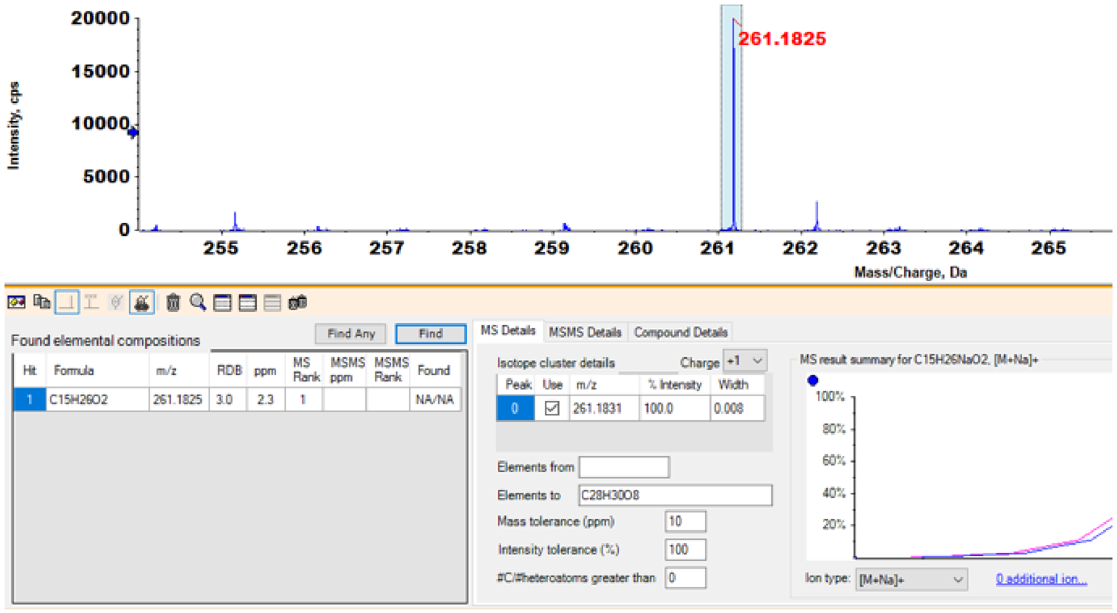

Figure S43: HRESIMS of **5**.

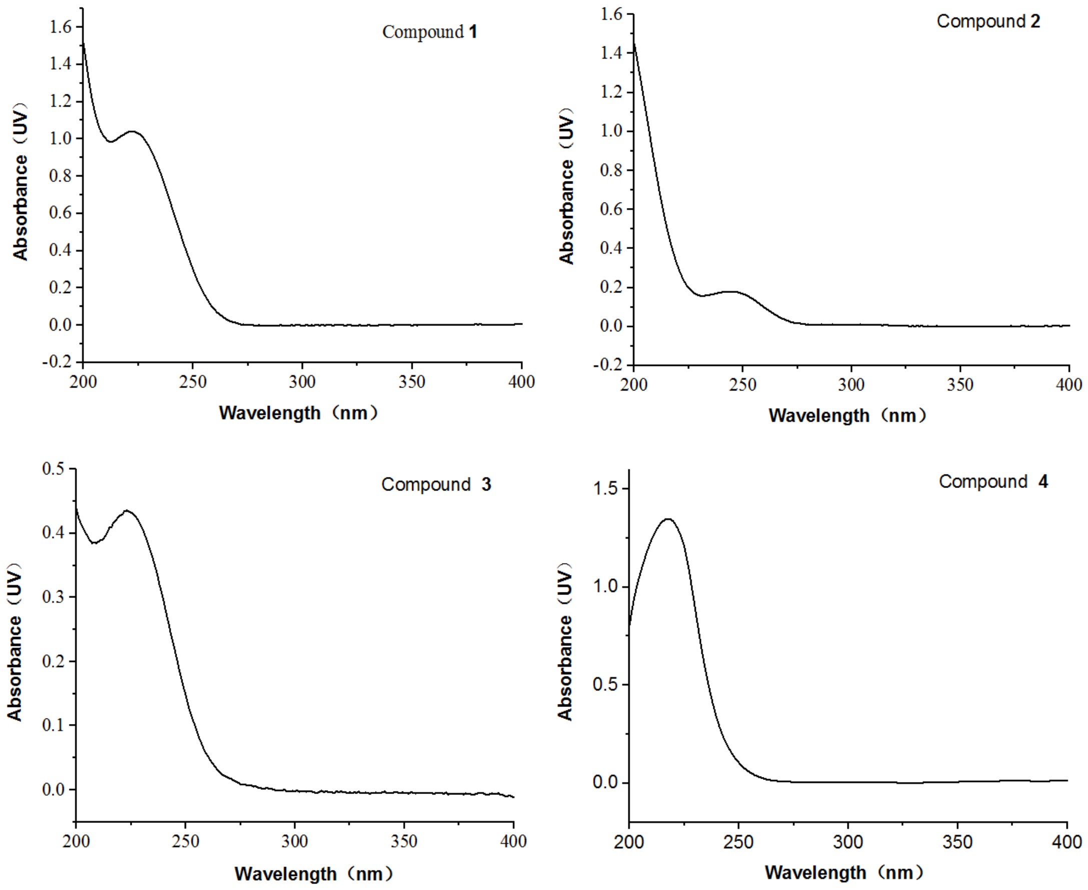

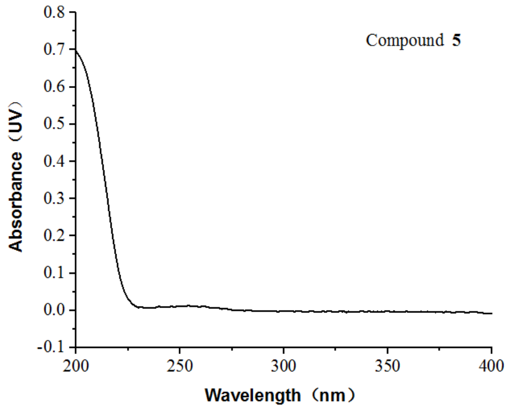

Figure S44: UV–vis spectra of **1–5**

Bioactivity assay data

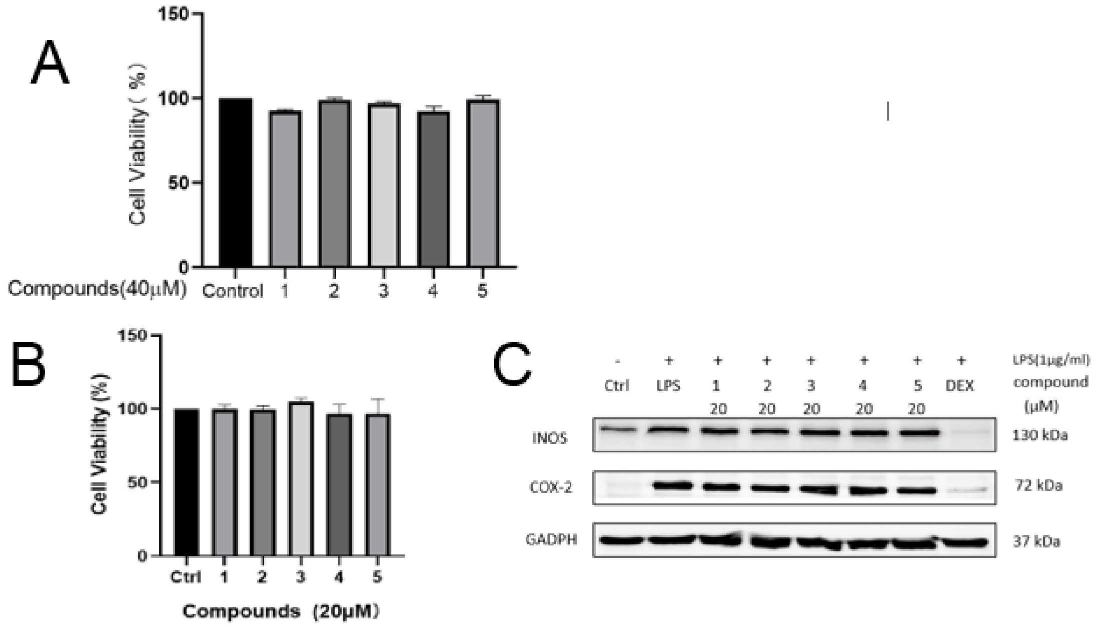

Fig A: human breast cancer cell line (MCF-7) were treated with 40 μM with conpounds **1–5** for 24 h, and cell viability was determined by CCK-8 assay.

Fig B: RAW 264.7 were treated with 20 μM with conpounds **1–5** for 24 h, and cell viability was determined by CCK-8 assay.

Fig C: Compounds suppress LPS-induced INOS and COX-2 expression in RAW 264.7 cells. Data represent mean ±SEM values of three experiments.
